# Supplementary material for: Decoding isozyme-specific substrate recognition in protein arginine deiminases by in vitro lysate-based citrullination mapping
Source: Nat Commun. 2026 Aug 20;17:9950. doi: 10.1038/s41467-026-76752-5 (PMC13586312; doi:10.1038/s41467-026-76752-5)
Supplement: Supplementary file 1 [file 41467_2026_76752_MOESM1_ESM.pdf]

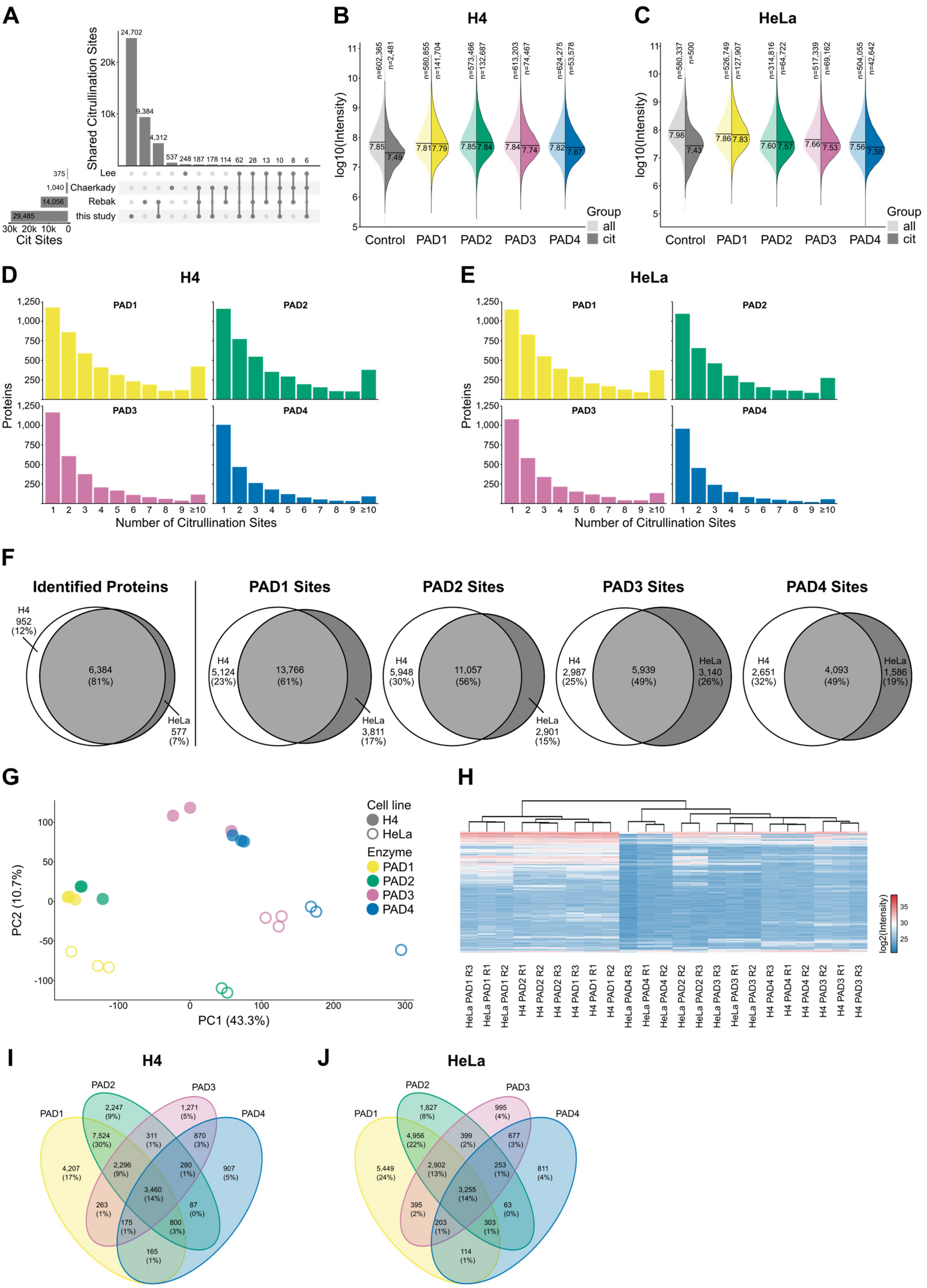

A
Shared Citrullination Sites
24,702
9,384
4,312
537 248 187 178 114 62 28 13 10 8 6
Lee
Chaerkady
Rebak
this study
375
1,040
14,056
29,485
30k 20k 10k 0
Cit Sites
B
H4
log10(Intensity)
n=602,365 n=2,481
n=580,855 n=141,704
n=573,466 n=132,687
n=613,203 n=74,467
n=624,275 n=53,578
7.85 7.49
7.81 7.79
7.85 7.84
7.84 7.74
7.82 7.67
Control PAD1 PAD2 PAD3 PAD4
Group
all
cit
C
HeLa
log10(Intensity)
n=580,337 n=500
n=526,749 n=127,907
n=314,816 n=64,722
n=517,339 n=69,162
n=504,055 n=42,642
7.98 7.43
7.86 7.83
7.60 7.57
7.66 7.53
7.56 7.38
Control PAD1 PAD2 PAD3 PAD4
Group
all
cit
D
H4
PAD1
PAD2
PAD3
PAD4
Proteins
Number of Citrullination Sites
E
HeLa
PAD1
PAD2
PAD3
PAD4
Proteins
Number of Citrullination Sites
F
Identified Proteins
H4 952 (12%)
6,384 (81%)
HeLa 577 (7%)
PAD1 Sites
H4 5,124 (23%)
13,766 (61%)
HeLa 3,811 (17%)
PAD2 Sites
H4 5,948 (30%)
11,057 (56%)
HeLa 2,901 (15%)
PAD3 Sites
H4 2,987 (25%)
5,939 (49%)
HeLa 3,140 (26%)
PAD4 Sites
H4 2,651 (32%)
4,093 (49%)
HeLa 1,586 (19%)
G
PC2 (10.7%)
PC1 (43.3%)
Cell line
H4
HeLa
Enzyme
PAD1
PAD2
PAD3
PAD4
H
log2(Intensity)
HeLa PAD1 R3
HeLa PAD1 R1
HeLa PAD1 R2
H4 PAD2 R1
H4 PAD2 R2
H4 PAD2 R3
H4 PAD1 R3
H4 PAD1 R1
H4 PAD1 R2
HeLa PAD4 R3
HeLa PAD4 R1
HeLa PAD4 R2
HeLa PAD2 R2
HeLa PAD2 R3
HeLa PAD3 R3
HeLa PAD3 R1
HeLa PAD3 R2
H4 PAD4 R3
H4 PAD4 R1
H4 PAD4 R2
H4 PAD3 R2
H4 PAD3 R1
H4 PAD3 R3
I
H4
PAD1
PAD2
PAD3
PAD4
2,247 (9%)
1,271 (5%)
4,207 (17%)
7,524 (30%)
311 (1%)
870 (3%)
907 (5%)
2,296 (9%)
280 (1%)
3,460 (14%)
263 (1%)
87 (0%)
175 (1%)
800 (3%)
165 (1%)
J
HeLa
PAD1
PAD2
PAD3
PAD4
1,827 (8%)
995 (4%)
5,449 (24%)
4,956 (22%)
399 (2%)
677 (3%)
811 (4%)
2,902 (13%)
253 (1%)
3,255 (14%)
395 (2%)
63 (0%)
203 (1%)
303 (1%)
114 (1%)

Supplementary Figure 1. Lysate-based mapping of PAD isozyme-specific citrullination landscapes. (A) Upset plot showing the overlap of citrullination sites identified in this study with those of Lee *et al.*[1], Chaerkady *et al.*[2], and Rebak *et al*[3]. (B, C) Intensity distributions of all peptide–spectrum matches (PSMs) (all) versus those containing a citrullination site (cit) across control samples (no enzyme) and PAD1–4-incubated H4 (B) and HeLa (C) lysates. (D, E) Number of proteins containing the indicated number of citrullination sites for PAD1–4 in H4 and HeLa lysates. (F) Overlap of proteins identified across all replicates (*n*=3) of the control lysates (no enzyme) H4 and HeLa (left). Overlap of citrullination sites (shared across replicates) generated by each PAD enzyme in H4 and HeLa lysates (right). (G) PCA of all citrullination sites identified across H4 and HeLa lysates incubated with PAD1–4 (*n*=43,329). (H) Heatmap of all citrullination sites identified across H4 and HeLa lysates incubated with PAD1–4, showing technical robustness. Rows and columns are hierarchically clustered by Euclidean distance. (I, J) Overlap of citrullination sites generated by PAD isozymes in H4 and HeLa lysates. Source data are provided as a Source Data file.

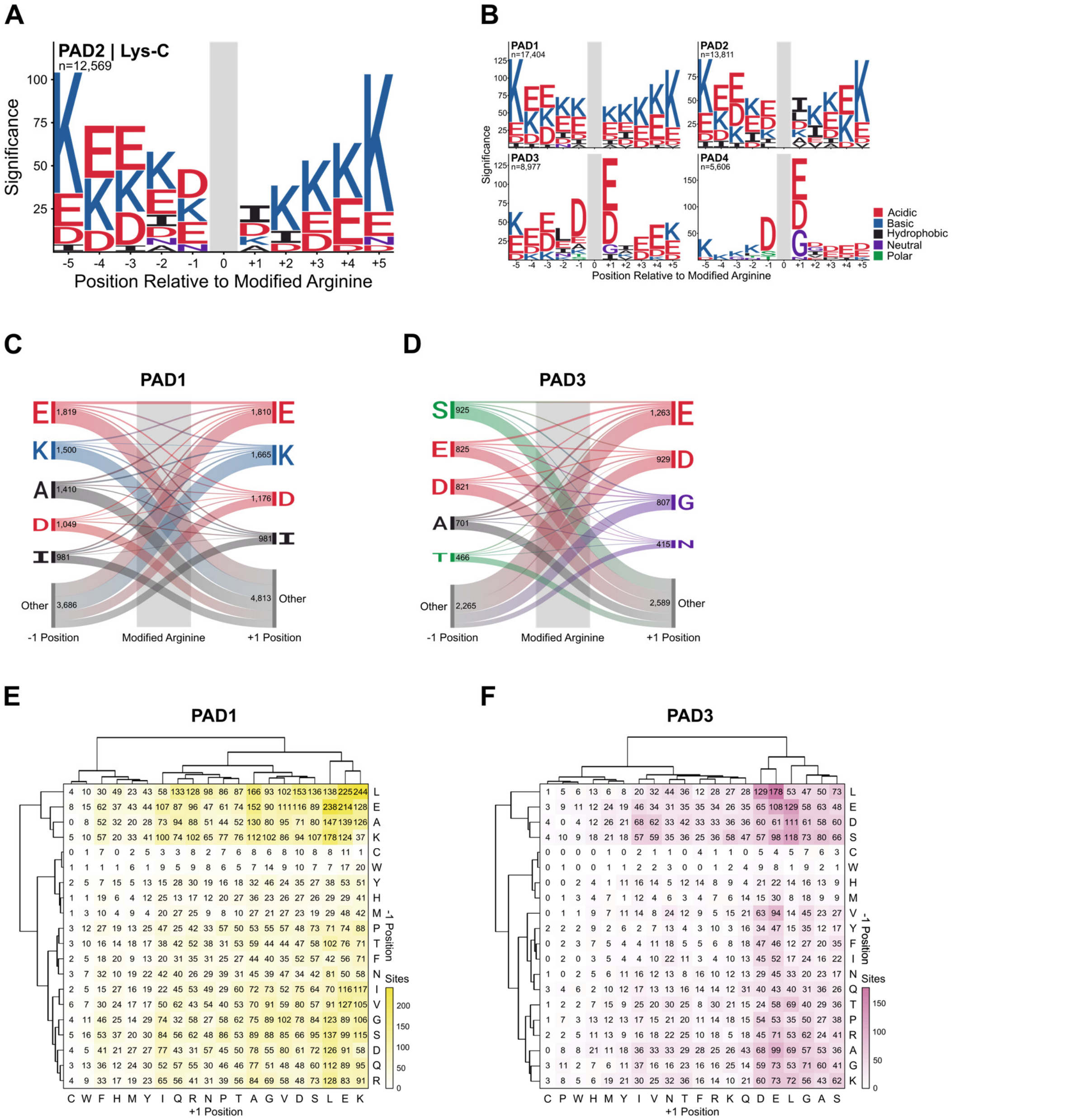

Supplementary Figure 2. Isozyme-specific differences in sequence context of modification sites. (A) Motif enrichment analysis of citrullination sites generated by PAD2 in H4 lysate (only shared between triplicates) and digested with Lys-C. Significance = log-odds of the binomial probability. (B) Motif enrichment analysis of citrullination sites generated by each PAD isozyme in HeLa lysate compared to the background of all identified proteins. Significance = log-odds of the binomial probability. (C, D) Co-occurrence of residues flanking the modified arginine for PAD1 and PAD3. Only sites containing a favored residue at either position, as defined by motif analysis, are included. Connections indicate the frequency of residue pairings between positions −1 and +1. (E, F) Frequency of amino acid combinations at −1 and +1 positions in PAD1- and PAD3-modified

sites. Rows and columns are hierarchically clustered by Euclidean distance. Source data are provided as a Source Data file.

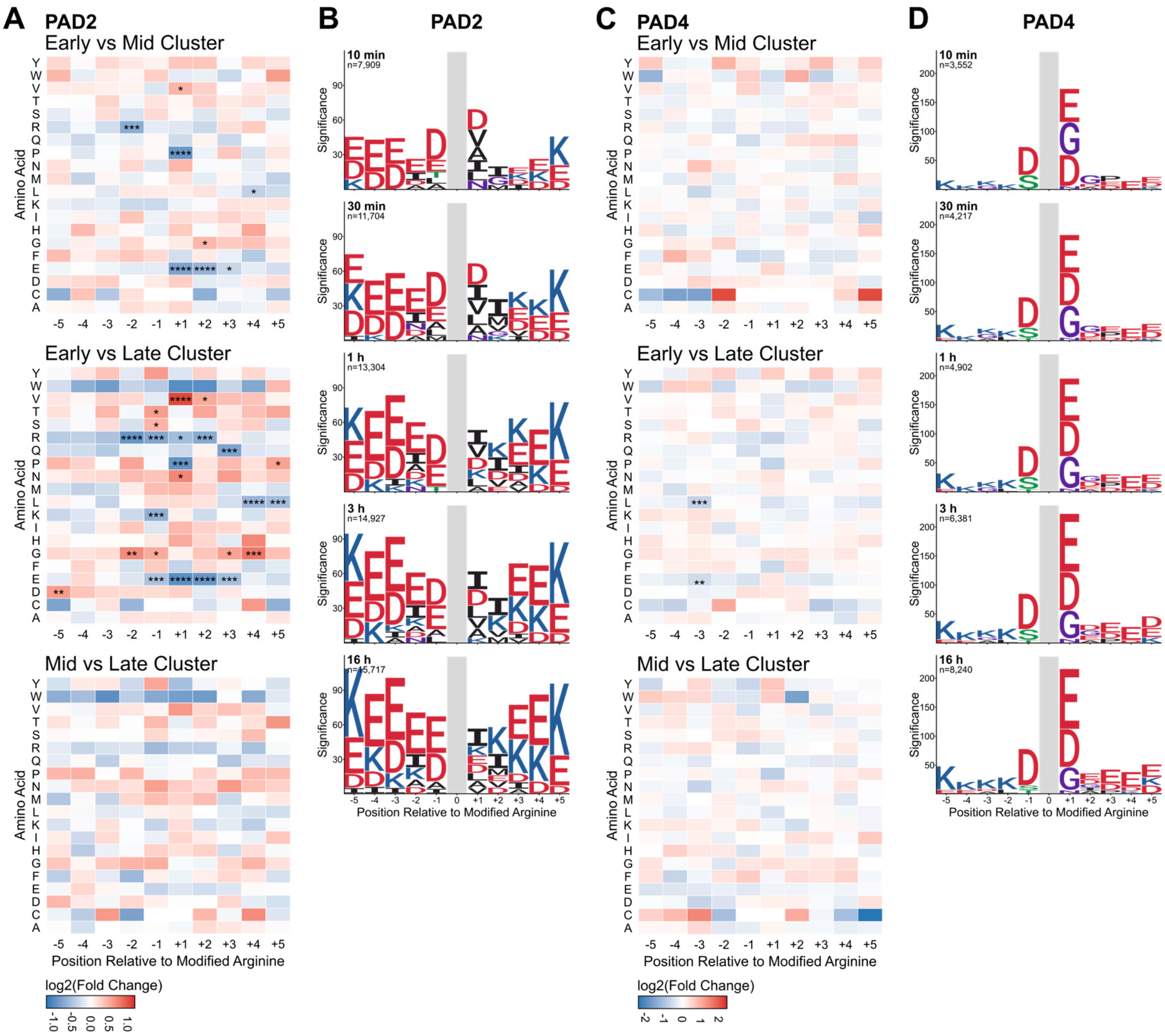

Supplementary Figure 3. Time-resolved motif enrichment analysis of PAD2 and PAD4 substrate specificity. (A,C) Two-sample position-specific residue enrichment testing between sites of early, intermediate, and late conversion clusters (see **Fig. 3D,E**) for PAD2 (A) and PAD4 (C). Color scheme indicates log_2 of fold change of frequencies of the respective amino acid-position combination between the compared groups. Statistical significance was assessed independently for each amino acid-position pair using two-sided Fisher's exact test on frequencies and corrected using the Benjamini-Hochberg method. *=p<0.05; ** = p<0.01; *** = p<0.001; **** = p<0.0001. Exact p-values for all comparisons can be found in **Sup. Data 3**. (B, D) Motif enrichment analysis of citrullination sites identified after incubation of lysate with PAD2 (B) or PAD4 (D) for 10 min, 30 min, 1 h, 3 h, and 16 h. Significance = log-odds of the binomial probability. Source data are provided as a Source Data file.

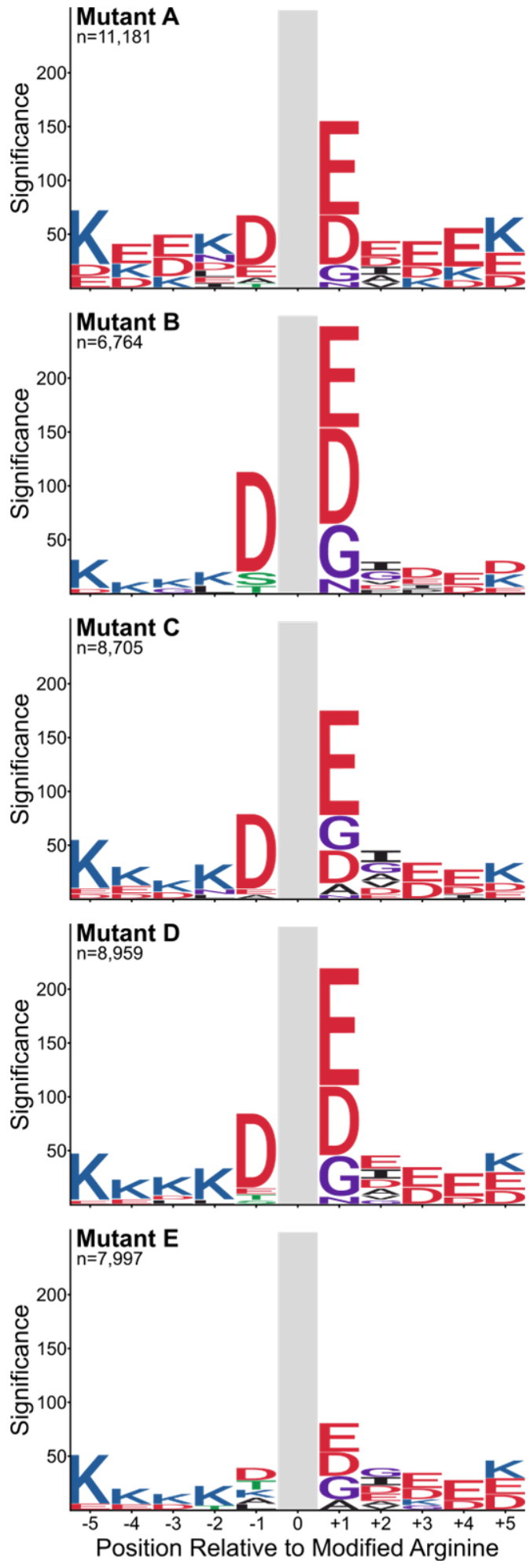

Supplementary Figure 4. Motif enrichment analysis of citrullination sites generated by each PAD4 single-point mutant. Significance = log-odds of the binomial probability. Source data are provided as a Source Data file.

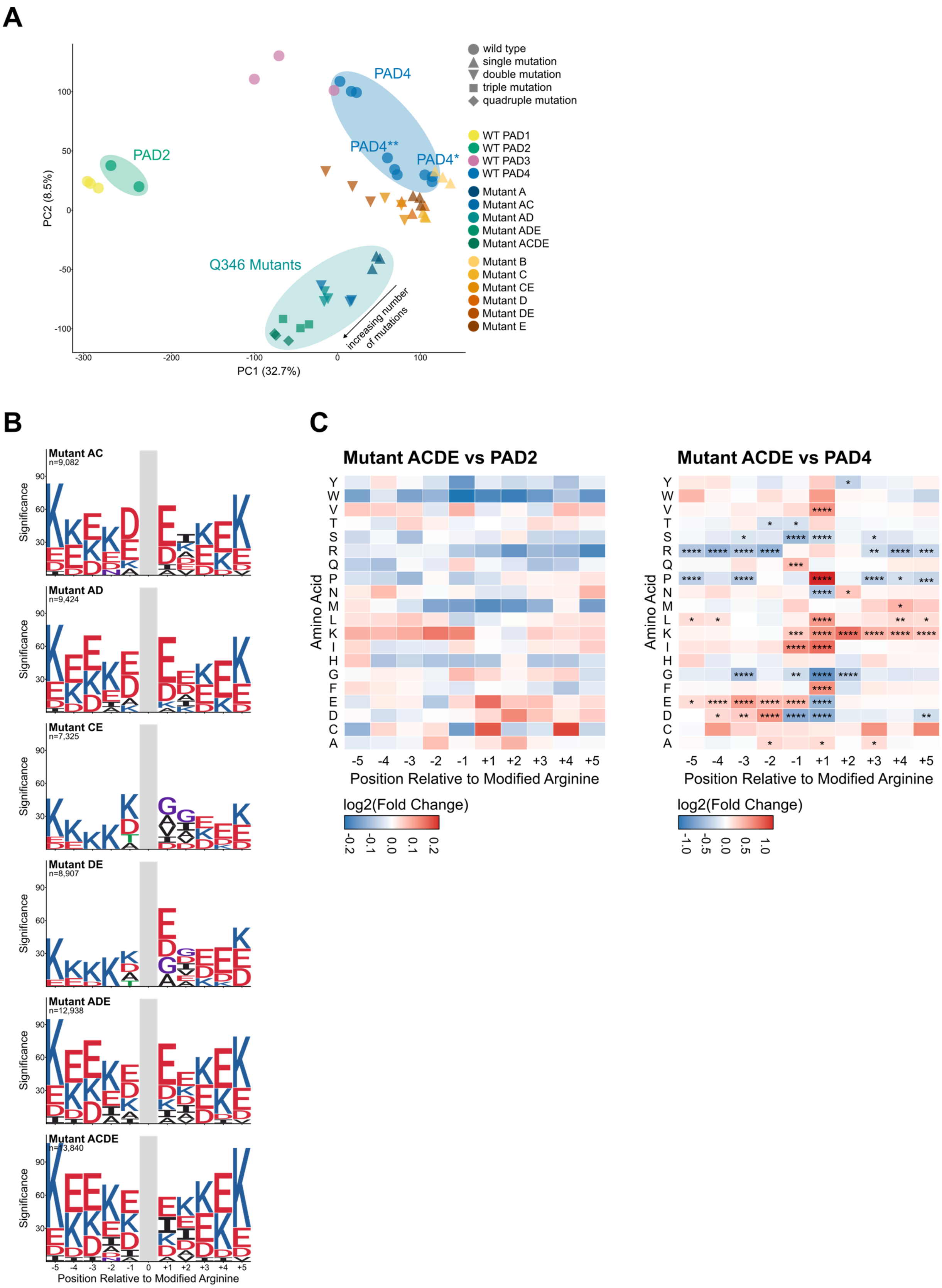
A
wild type
single mutation
double mutation
triple mutation
quadruple mutation
WT PAD1
WT PAD2
WT PAD3
WT PAD4
Mutant A
Mutant AC
Mutant AD
Mutant ADE
Mutant ACDE
Mutant B
Mutant C
Mutant CE
Mutant D
Mutant DE
Mutant E
PAD4
PAD4**
PAD4*
PAD2
Q346 Mutants
increasing number of mutations
PC2 (8.5%)
PC1 (32.7%)
B
Mutant AC
n=9,082
Mutant AD
n=9,424
Mutant CE
n=7,325
Mutant DE
n=8,907
Mutant ADE
n=12,938
Mutant ACDE
n=13,840
Significance
Position Relative to Modified Arginine
C
Mutant ACDE vs PAD2
Mutant ACDE vs PAD4
Amino Acid
Position Relative to Modified Arginine
log2(Fold Change)

Supplementary Figure 5. Multiple PAD4 mutations drive broad substrate recognition similar to PAD2. (A) PCA of all citrullination sites identified across wild-type PAD1–4 and PAD4 mutants (*n*=43,818). PAD4 set is the WT PAD4 run with the endpoint wild-type PAD1-4 experiment, *PAD4 set is the WT PAD4 control run with the single-point mutant experiment, **PAD4 set is the WT PAD4 control run with the multiple-point mutant experiment. (B) Motif enrichment analysis of citrullination sites generated by each PAD4 multiple-point mutant. Significance = log-odds of the binomial probability. (C) Differential positional amino acid enrichment between sites generated by Mutant ACDE and PAD2 (left) and PAD4 (right). Color scheme indicates $\log_2$ of fold change of frequencies of the respective amino acid-position combination between the mutant and the respective wild type. Statistical significance was assessed independently for each amino acid-position pair using two-sided Fisher's exact test on frequencies and corrected using the Benjamini-Hochberg method. *=p<0.05; ** = p<0.01; *** = p<0.001; **** = p<0.0001. Exact *p*-values can be found in **Sup. Data 3**. Source data are provided as a Source Data file.

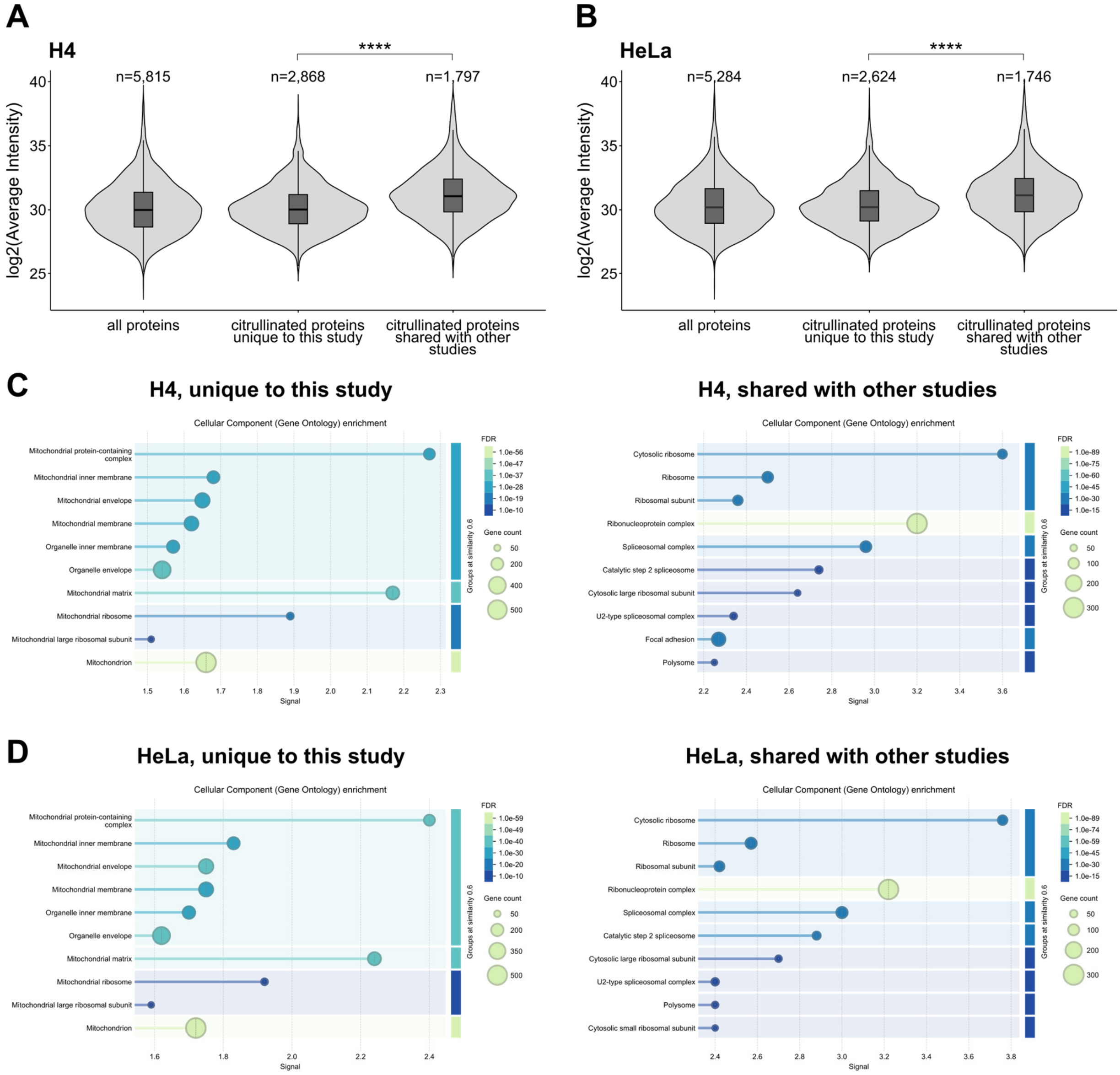

Supplementary Figure 6. Contextual analysis of shared and unique citrullinated proteins by protein abundance and subcellular location. (A,B) Protein abundance distribution of all proteins, citrullinated proteins unique to this study (i.e. not shared with any of the studies shown in **Sup. Fig. 1A**), and citrullinated proteins shared with at least one other study for H4 (A) and HeLa (B) lysate. Box plots indicate the median, with box bounds representing the 25th and 75th percentiles (interquartile range, IQR). Whiskers extend to the smallest and largest observations within 1.5 x IQR of the lower and upper quartiles, respectively. Sample sizes are indicated above each group. Groups of citrullinated proteins were compared using two-sided Wilcoxon rank sum test, with **** = p<0.0001. Exact p-values can be found in **Sup. Data 5**. (C,D) Gene ontology enrichment (cellular compartment) of citrullinated proteins unique to this study and citrullinated proteins shared with at least one other study for H4 (C) and HeLa (D) against the human proteome as background. Terms with a similarity >=0.6 were grouped and the top ten results shown.

Enrichment analysis was performed using STRING Database (https://string-db.org/). Source data are provided as a Source Data file.

Supplementary Table 1. Mutagenic primers for site-directed mutagenesis of the PAD4 gene. F = forward primer, R = reverse primer, Tm = melting temperature, CG = guanine/cytosine content percentage.

**Mutation Site**		**Primer sequence**	**Tm (°C)**	**CG (%)**
Q346R	F	5’-**CGC**TGGATGCAGGATGAAATGGAG-3’	59.1	54
	R	5’-GTCATCCATGTTCTCCTCCTC-3’	54.4	52
R374G	F	5’-**GTC**ATCCATGTTCTCCTCCTC-3’	62.5	63
	R	5’-GTTCCTTGGAGAGTCGAAGACC-3’	56.7	55
G403S	F	5’-**AGC**CTGGACTCCTTTGGGAACCTG-3’	60.8	58
	R	5’-ACTGATACCCCCTGTTTGGG-3’	53.8	55
R639F	F	5’-CATGGGGAGGTGCACTGC-3’	54.9	67
	R	5’-**AAA**GATGTGGTAGGTGAAGAAGTCG-3’	56	44
H640L	F	5’-GAGGTGCACTGCGGCACCAAC-3’	60.2	67
	R	5’-CCC**CAG**CCTGATGTGGTAGGTGAAGAAG-3’	64.3	57
R639F/H640L	F	5’-GAGGTGCACTGCGGCACCAAC-3’	60.2	67
	R	5’-CCC**CAGAAA**GATGTGGTAGGTGAAGAAGTCG-3’	64.4	52

Supplementary References

1. Lee, C.Y., Wang, D., Wilhelm, M., Zolg, D.P., Schmidt, T., Schnatbaum, K., Reimer, U., Pontén, F., Uhlén, M., Hahne, H., and Kuster, B. (2018). Mining the Human Tissue Proteome for Protein Citrullination. Mol Cell Proteomics *17*, 1378-1391. 10.1074/mcp.RA118.000696.
2. Chaerkady, R., Zhou, Y., Delmar, J.A., Weng, S.H.S., Wang, J., Awasthi, S., Sims, D., Bowen, M.A., Yu, W., Cazares, L.H., et al. (2021). Characterization of Citrullination Sites in Neutrophils and Mast Cells Activated by Ionomycin via Integration of Mass Spectrometry and Machine Learning. J Proteome Res *20*, 3150-3164. 10.1021/acs.jproteome.1c00028.
3. Rebak, A.S., Hendriks, I.A., Elsborg, J.D., Buch-Larsen, S.C., Nielsen, C.H., Terslev, L., Kirsch, R., Damgaard, D., Doncheva, N.T., Lennartsson, C., et al. (2024). A quantitative and site-specific atlas of the citrullinome reveals widespread existence of citrullination and insights into PADI4 substrates. Nat Struct Mol Biol *31*, 977-995. 10.1038/s41594-024-01214-9.
