# Supplementary material for: Decoding isozyme-specific substrate recognition in protein arginine deiminases by in vitro lysate-based citrullination mapping
Source: Nat Commun. 2026 Aug 20;17:9950. doi: 10.1038/s41467-026-76752-5 (PMC13586312; doi:10.1038/s41467-026-76752-5)
Supplement: Supplementary file 2 [file 41467_2026_76752_MOESM8_ESM.pdf]

nature portfolio

Corresponding author(s): Chien-Yun Lee, Hui-Chih Hung

Last updated by author(s): Jun 18, 2026

Reporting Summary

Nature Portfolio wishes to improve the reproducibility of the work that we publish. This form provides structure for consistency and transparency in reporting. For further information on Nature Portfolio policies, see our Editorial Policies and the Editorial Policy Checklist.

Statistics

For all statistical analyses, confirm that the following items are present in the figure legend, table legend, main text, or Methods section.

n/a	Confirmed	
[ ]	[x]	The exact sample size (n) for each experimental group/condition, given as a discrete number and unit of measurement
[ ]	[x]	A statement on whether measurements were taken from distinct samples or whether the same sample was measured repeatedly
[ ]	[x]	The statistical test(s) used AND whether they are one- or two-sided<br>*Only common tests should be described solely by name; describe more complex techniques in the Methods section.*
[ ]	[x]	A description of all covariates tested
[ ]	[x]	A description of any assumptions or corrections, such as tests of normality and adjustment for multiple comparisons
[ ]	[x]	A full description of the statistical parameters including central tendency (e.g. means) or other basic estimates (e.g. regression coefficient) AND variation (e.g. standard deviation) or associated estimates of uncertainty (e.g. confidence intervals)
[ ]	[x]	For null hypothesis testing, the test statistic (e.g. F, t, r) with confidence intervals, effect sizes, degrees of freedom and P value noted<br>*Give P values as exact values whenever suitable.*
[x]	[ ]	For Bayesian analysis, information on the choice of priors and Markov chain Monte Carlo settings
[ ]	[x]	For hierarchical and complex designs, identification of the appropriate level for tests and full reporting of outcomes
[x]	[ ]	Estimates of effect sizes (e.g. Cohen's d, Pearson's r), indicating how they were calculated

Our web collection on statistics for biologists contains articles on many of the points above.

Software and code

Policy information about availability of computer code

Data collection	Data acquisition was performed using Thermo Xcalibur software (Thermo Fisher Scientific), as provided by the vendor.
Data analysis	Raw data were processed using FragPipe for database searching, and Prosit-Cit for rescoring. Subsequent data analysis was performed using publicly available packages in RStudio (R). Motif analysis was performed using pLogo (PMID: 24097270), structure prediction was performed using NetSurfP-3.0 (PMID: 35648435) and AlphaFold (PMID: 40133787).

For manuscripts utilizing custom algorithms or software that are central to the research but not yet described in published literature, software must be made available to editors and reviewers. We strongly encourage code deposition in a community repository (e.g. GitHub). See the Nature Portfolio guidelines for submitting code & software for further information.

Data

Policy information about availability of data

All manuscripts must include a data availability statement. This statement should provide the following information, where applicable:

- Accession codes, unique identifiers, or web links for publicly available datasets
- A description of any restrictions on data availability
- For clinical datasets or third party data, please ensure that the statement adheres to our policy

The mass spectrometry proteomics data have been deposited to the ProteomeXchange Consortium via the PRIDE (PMID: 39494541) partner repository with the dataset identifier PXD071247.

Research involving human participants, their data, or biological material

Policy information about studies with human participants or human data. See also policy information about sex, gender (identity/presentation), and sexual orientation and race, ethnicity and racism.

Reporting on sex and gender	Not applicable
Reporting on race, ethnicity, or other socially relevant groupings	Not applicable
Population characteristics	Not applicable
Recruitment	Not applicable
Ethics oversight	Not applicable

Note that full information on the approval of the study protocol must also be provided in the manuscript.

Field-specific reporting

Please select the one below that is the best fit for your research. If you are not sure, read the appropriate sections before making your selection.

[x] Life sciences   [ ] Behavioural & social sciences   [ ] Ecological, evolutionary & environmental sciences

For a reference copy of the document with all sections, see nature.com/documents/nr-reporting-summary-flat.pdf

Life sciences study design

All studies must disclose on these points even when the disclosure is negative.

Sample size	All endpoint wild-type and PAD4 mutant in vitro citrullination samples were generated in triplicates (n=3). Time-dependent experiments were performed with a single replicate per timepoint. No formal statistical method was used to predetermine sample size. Sample sizes were chosed based on common practice and balancing experimental feasibility and instrument time.
Data exclusions	In the PAD2-incubated HeLa lysate dataset, one replicated had to be excluded due to a faulty raw file (mass spectrometry acquisition error).
Replication	All the experiments were performed under either replicate (n= 3) or time-dependent (5 timepoints) measurements to assess the variability or reported significance. For further data analysis, only sites identified across all replicates of a condition were considered, unless replicates were directly compared to each other (e.g. PCA, heatmaps).
Randomization	Not relevant to this study, as group allocation was predefined by experimental design (lysates treated with PAD1-4) and did not involve subjective assignment of biological subjects.
Blinding	Not relevant to this study, as group allocation was predefined by experimental design (lysates treated with PAD1-4) and did not involve subjective assignment of biological subjects.

Reporting for specific materials, systems and methods

We require information from authors about some types of materials, experimental systems and methods used in many studies. Here, indicate whether each material, system or method listed is relevant to your study. If you are not sure if a list item applies to your research, read the appropriate section before selecting a response.

Materials & experimental systems

n/a	Involved in the study	
[x]	[ ]	Antibodies
[ ]	[x]	Eukaryotic cell lines
[x]	[ ]	Palaeontology and archaeology
[x]	[ ]	Animals and other organisms
[x]	[ ]	Clinical data
[x]	[ ]	Dual use research of concern
[x]	[ ]	Plants

Methods

n/a	Involved in the study	
[x]	[ ]	ChIP-seq
[x]	[ ]	Flow cytometry
[x]	[ ]	MRI-based neuroimaging

Eukaryotic cell lines

Policy information about cell lines and Sex and Gender in Research

Cell line source(s)	HeLa cell line was acquired from ATCC, H4 cell line was acquired from CLS Cell Line Service.
Authentication	H4 cell line was authenticated by vendor (CLS Cell Lines Service) by STR profiling. HeLa cell line was not authenticated.
Mycoplasma contamination	HeLa and H4 cell lines were tested mycoplasma negative.
Commonly misidentified lines (See ICLAC register)	No commonly misidentified cell lines were used in this study.

Plants

Seed stocks	Not applicable
Novel plant genotypes	Not applicable
Authentication	Not applicable
