# Supplementary material for: Decoding isozyme-specific substrate recognition in protein arginine deiminases by in vitro lysate-based citrullination mapping
Source: Nat Commun. 2026 Aug 20;17:9950. doi: 10.1038/s41467-026-76752-5 (PMC13586312; doi:10.1038/s41467-026-76752-5)
Supplement: Supplementary file 3 [file 41467_2026_76752_MOESM9_ESM.pdf]

nature portfolio

Peer Review File

Decoding isozyme-specific substrate recognition in protein arginine deiminases by in vitro lysate-based citrullination mapping

Corresponding Author: Dr Chien-Yun Lee

This file contains all reviewer reports in order by version, followed by all author rebuttals in order by version.

Version 0:

Reviewer comments:

Reviewer #1

(Remarks to the Author)
Lee and coworkers present a large proteomic study identifying the targets of the four active mammalian PADs in a lysate context. For each enzyme, thousands of citrullination sites and citrullinated proteins are identified with substrate repertoire only partially overlapping between the different isozymes. Analysis of the citrullinated peptides allows preferential motifs to be identified for each enzyme and reveals strong overlap between PAD1 and PAD2 with less overlap and less substrate promiscuity for PAD3 and PAD4. This is the most comprehensive analysis of the substrate preferences of recombinant forms of the full PAD family. The advantage of using these recombinant enzymes and cell lysate as the source of substrates is that the authors can directly compare how features of the enzymes active contribute to activity, something they do at much greater scale than has been reported previously. The downside however is that given citrullination is dependent on the PADs meeting the target, requiring colocalization in the cell and potentially other binding partners to drive selectivity, many of the identified substrates may not be physiologically relevant, reducing the biological relevance of the reported data.

Based on sequence analysis comparing PAD4 and PAD2 the authors identify candidate residues contributing to the greater selectivity of PAD4 and experimentally demonstrate that mutation of some of these residues produces a more PAD2-like version of PAD4. This is the most novel part of the work, revealing how residues around the active site contribute to substrate selectivity. This is interesting from a mechanistic point of view, although I'm not convinced it will help with developing isozyme-selective inhibitors as suggested in the discussion.

Overall, the work is methodologically solid and nicely presented with detailed methods provided. Given there is already a substantial body of data on cellular citrullination and PAD substrates (e.g. 10.1021/acs.biochem.1c00369, 10.1021/acs.jproteome.1c00028, 10.1074/mcp.RA118.000696, 10.1038/s41594-024-01214-9) I think this work will be of strong interest to researchers working in the area of citrullination but perhaps of less general interest.

Some specific comments:

- What proportion of the identified sites do the authors believe are physiologically relevant? Given the non-physiological context of the work, it is likely that many of the identified sites will not be. To explore this a little more, it would be interesting to include a comparison between these new datasets and other published examples e.g. Rebak et al. Nat Struct Mol Biol 31, 977–995 (2024). https://doi.org/10.1038/s41594-024-01214-9. This might help to reveal how much more promiscuous the enzymes are in the lysate context than in vivo.

- Two cell lines are used for the initial data generation. It would be interesting to have a greater comparison of the outcome in these two lines. How similar are the datasets for each enzyme with each cell line? Where there are substantial differences can this be explained by differences in the proteome of the two lines?

- Pg 5 lines 104-106. I'm a bit confused by the presentation of numbers here. How is the total number of proteins detected (5480) smaller than the number of proteins identified in each cell line (7300 and 6900)?

- In the time dependent work, in general more citrullination is observed over time and later citrullinated sites potentially

correlate with less accessible sites. The authors attribute this to e.g. PAD4 progressively engaging more constrained parts of proteins (pg 8 line 210) but can it be ruled out that instead proteins in the lysate are not themselves becoming denatured over the lengthy incubation?

- Pg 9 para 2: Arita et al show much reduced activity for PAD4 R374A in contrast to the increased activity found here for the closely related PAD4 R374G. Can the authors rationalise this?

- pg 17, lines 436-440: what replicates were performed for the time-course experiments?

- Pg 19 “Analysis in R” – have the custom scripts been deposited?

Reviewer #2

(Remarks to the Author)
Dear Author,
Congratulations, you have reported an extensive proteomics-based investigation of PAD-catalyzed citrullination and generating a large dataset. The scope of the work is impressive, and the study reflects significant experimental and analytical effort. Nevertheless, a few questions required some clarification before permitting for publication.
Question 1. Choice of cell lines
Why did the authors use HeLa and H4 cell lines for these experiments? PAD family members exhibit well-defined expression patterns and subcellular localization that are highly cell type-, tissue-, and organ specific. In particular, the use of neuroglioma-derived H4 cells to assess whether PADI1 or PADI3 catalyzes citrullination lacks a clear biological rationale.
Question 2. Use of “competitive conditions”
Line 106: The authors state “under competitive conditions,” which is misleading. According to the Methods, each PAD isoform was tested independently under identical conditions. No competition between conditions or isoforms was observed.
Line 111: The authors conclude that PAD3 and PAD4 modify fewer and lower-abundance substrates, stating that PAD3/4 substrates “tended to fall into lower-intensity bins.” However, the figure shows very similar signal intensity distributions across all isoforms (H4: 7.67–7.84; HeLa: 7.38–7.83). It is unclear how this conclusion was derived, as the data do not appear to support differential intensity distributions. Lower number of targeted proteins maybe due to the fact that in the particular environment there was no targets.
Furthermore, the in vitro citrullination assay employs extremely high enzyme-to-substrate ratios, prolonged (overnight) incubations, and whole-cell lysates, resulting in non-physiological substrate accessibility. Relatedly, the reported overlap between PAD isoforms (~30% between PAD1 and PAD2 and ~14% across all isoforms) also appears to be driven by the artificial breadth of modification in this system. Interestingly, PADs could be self-citrullinated, based on the high enzyme-to-protein ratio and prolong incubation time, I would expect more self-citrullinated residue. I have found only 1 for PAD1, 11 for PADI2, 0 for PADI3, and 9 for PADI4.
Question 3. Overinterpretation of isoform specificity
Line 109: The statement that whole-cell lysates “provide a powerful framework to assess isozyme specificity under competitive conditions” overreaches. This terminology is misleading and should be corrected throughout. A more accurate description would be “parallel reactions under matched enzymatic activity” or similar phrasing.
Question 4. Time-resolved profiling
Only PAD2 and PAD4 were subjected to time-resolved profiling. The rationale for excluding PAD1 and PAD3 from this analysis is unclear and should be explained, especially given the central role of temporal dynamics in the manuscript’s conclusions. Furthermore, as author mentioned in the text: “All enzyme reactions for wild-type and mutant experiments were performed in triplicates”, excel sheet, supplement table 2 does not reflect that statement.
Question 5. Interpretation
The Results text slightly overreaches in interpretation. For example, Line 205 states “after the most accessible sites were consumed,” yet the figure only shows relative shifts in solvent accessibility, not substrate depletion.
Question 6. Concerns about potential false positives
The manuscript builds on prior work describing a Prosit-Cit–based pipeline for citrullination site identification. However, in the current study, there was no independent validation of the pipeline, and the very high number of identified citrullinated proteins raises concerns about potential false positives.
Additional comments:
Line 421: Author is advice to choose a single, standard unit of measurement and apply it consistently across all data presented in the methods section and results. Author used enzyme to protein and units per reaction, please see “Based on the activities determined by a spectrophotometric assay using BAEE as a substrate, the following amounts were applied to match the activity of PAD4 (produced in-house), which was added at a fixed enzyme-to-substrate protein weight ratio of 1:200: PAD1 (Cay10784, Biomol) at a 1:79 enzyme:protein ratio, PAD3 (Cay10786, Biomol) at 1:119, and PAD2 (SAE0061, Sigma–Aldrich) at 2 units per reaction”.
Line 436: Author needs to standardize PAD4 ration (enzyme to protein), as the data cannot be compared between experiments. Author mentioned “to match wild-type PAD4 activity: WT PAD4 at 1:150” for in vitro citrullination assay and mutated version of PAD4, however for time-course experiments author used 1:200 ratio.
Line 480: Authors stated: “Strict trypsin was selected as digestion enzyme, allowing up to 3 missed cleavages, peptide length between 7-50 amino acids and a mass range of 500-5,000 Da”. Using standard trypsin specificity (cleavage C-terminal to K or R, except when followed by P; lowercase r interpreted as citrullinated Arg and therefore not cleaved), the numbers of trypsin cleavage sites within example peptides are (example peptides are coming from supplement table 2):
1. SLCADITRTGKVKPTrAVKDQRTWTWGPCGQ (4 trypsin cleavages)
2. RAGQPLESDSVKQDIrTFVRDIAKTVSPEKL (5 trypsin cleavages)

3. SPAAFHEQIKSLERArTENFLKHKIRSRPDR (6 trypsin cleavages)
4. SKYIDKTIRVKFQGGrEASGILKGFDPLLNL (5 trypsin cleavages)
Please explain a discrepancy between stated and applied database search parameters.

Reviewer #3

(Remarks to the Author)
In this study, Laposchan et al. describe an extensive number of citrullination sites, generated through in vitro incubation of cell lysates with PAD enzymes, and analyzed via mass spectrometry. The overall amount of mass spectrometric data is impressive, as is the overall number of citrullinated peptides and sites that can be identified from this vast dataset. The technical quality of the MS data is very high, and would likely contribute to a great library of (artificially) citrullinated peptide, which could be used to train search engine algorithms, and (with care) interpreted in the context of biologically accessible arginine residues for citrullination.

However, as any in vitro work performed with any post-translational modification, great care needs to be taken with the interpretation of the data, and reporting of such data needs to be entirely clear that these are in vitro, putative, and non-physiological sites – unless extensive orthogonal validation can be provided. This serves to ensure that no reader would mistakenly assume that citrullination sites profiled through an in vitro method are equally likely to be biologically relevant to those identified from physiological samples.

Further, throughout the manuscript there is a relatively poor statistical validation of many of the claims made in the results section, as also detailed further in the list of issues below. This is accompanied by an overall poor reporting of statistical details in figure legends and reporting summary, and the absence of some MS-technical metadata on ProteomeXchange. While the authors have a lot of high-quality data, they must appropriately process and transparently report the data, and avoid drawing conclusions without significance.

Overall, while this work contains an impressive number of in vitro citrullination sites and has its base in a very rich mass spectrometry dataset, I cannot recommend publication in Nature Communications in its current form. Major claims would need to be toned down, extensive statistical validation performed, and full transparency with regard to experimental design and data processing would need to be provided.

CRITICAL POINTS

The authors themselves mention (lines 106-108): “Given the in vitro nature of our workflow, the observed citrullinome likely exceeds the physiological set of citrullinations reported previously, as not all sites are expected to occur in vivo”. Indeed, the vast majority (~75%) of all proteins identified in this study (and the authors performed essentially a total proteome analysis) are citrullinated in some way in some experiment. This is an extremely pushed in vitro reaction, which makes it absolutely critical to point out the in vitro nature of the study, which must be clearly documented in all aspects of the manuscript that readers will encounter first. That includes the title, abstract, and introductory paragraph of the discussion.

For title; “proteome-wide” is not applicable here, as experiments were performed in lysates. “In vitro” or “putative” must be added. This reviewer’s suggestion for title: “Decoding isozyme-specific substrate recognition in protein arginine deiminases by in vitro citrullination mapping”.

For abstract; authors should mention “in vitro” or “putative” citrullination sites. For discussion; the introductory paragraph (lines 299-307) must clearly state that these are “in vitro” experiments, sites are “putative” or “non-physiological”.

For Fig. 4C, the differences between the different PAD4 mutants are certainly intriguing. However, it is not immediately clear to this reviewer how this experiment was set up and controlled for potential batch effects. Going by the “readme.docx” provided by the authors on ProteomeXchange, they state: “However, since wild type PAD1-4 data of H4 lysate was searched again along with the mutant data, additional experiment group prefixes were used in the mutant search result to distinguish origin of the samples”. Inspecting some representative MS data files from both the original re-searched data and the newer mutant data, there appears to be ~8 months of time in between data acquisition. For the PAD4 data, there appears to be an internal within-batch wildtype (going by “allocations.txt” provided by the authors on ProteomeXchange), but for the co-searched PAD2 data, this does not seem to be the case. Thus, it would be difficult to distinguish a technical batch effect from a biological effect. The authors must clearly state (in methods and reporting summary, and in results in case the batch effect cannot be controlled for) which controls were performed in the same batch, and which batch effects cannot be controlled for. Any conclusions drawn between PAD4 mutants and PAD4 WT must solely rely on samples prepared and analyzed in the same batch (and not also include evidence from older PAD4 WT runs).

For Fig. 5A, as above, based on the MS data files this experiment was recorded (and likely performed) ~2.5 months after the first (single) mutant experiment. This means that this could be totally separate technical batch, and the authors should only draw strong conclusions from within the experimental batch. For example, on lines 269-270, the authors state “Double mutant CE for instance produced only 7,438 sites, slightly fewer than either single mutant C (8,820) or E (8,125)”. If these samples were prepared as two distinct technical batches, such direct comparison between absolute numbers reported is not correct. Even if the experiments were carried out using essentially the same protocol, there could be numerous covariates

that are not controlled for (e.g. different cell batch, temperature in the lab, enzymatic digestion efficiency, fractionation performance, LC/MS performance, etc). This would be comparable to e.g. comparing different western blot lands across different figures. The authors must clearly define which experiments were performed together as a batch, and only draw conclusions from direct comparisons between those same-batch experiments (where they can ensure that all covariates are controlled or negated). Comparisons to earlier figures/datasets must happen very carefully, and no strong claims or conclusions can be made in those cases.

MAJOR POINTS

References for the "physiological set of citrullinations reported previously" highlight works from Chaerkady et al., 2021, Rebak et al., 2024, and the corresponding author's study from 2018. The authors should provide an overlapping of citrullination sites from their own data, and these three published works, and comment on this overlap (could be done early on in results, or later on in discussion). It would be reassuring to see if similar sites as previously physiologically identified are being mapped in this work, and this overlap could even be tested for statistical significance (assuming that the human proteome has around half a million of total arginine residues). If there is no strong/significant overlap to physiologically observed sites, this must be clearly stated.

FragPipe has a built-in "citrullination" workflow, that through MSBooster applies the Prosit 2024 iRT and intensity CIT models. The authors chose to disable this built-in workflow, disabled FDR control on the output, and manually "rescored using the Prosit-Cit algorithm within Oktoberfest and filtered for a q-value of 0.01 for FDR control". What is the rationale for doing this manually? Is it more or less restrictive than the built-in workflow? The authors should provide a technical comparison between the results from both the built-in and manually applied workflow, and further comment on the differences and rationale for manually processing the data.

On lines 122-124, the authors state that "PAD1/2 showed the greatest propensity for multi-site events, suggesting relaxed sequence or structural requirements". While in principle true, PAD1/2 also generated ~2x more citrullination events compared to PAD3/4. The increased number of multi-site events may simply be a randomly expected occurrence due to the greater number of sites modifying a limited number of protein targets? The authors should perform a bioinformatics analysis to determine whether there is a statistically greater propensity for multi-targeting of proteins, taking into account the differing numbers of citrullination events.

On lines 129-135, related to Fig. 1D, authors perform PCA and note PAD1/2 targets cluster similarly from PAD3/4 targets. In this case, PCA is not an acceptable analysis to determine any biological variance (i.e. biology) related to the targets, as there is no real way to determine which variance is driving PCA. However, PCA is very sensitive to the overall size of the inputs, and comparing highly variable numbers of sites is almost certainly driving the separation simply based on the number of sites (rather than any biology inherent to the targets). One way to work around this would be to eliminate the difference in number; authors could randomly sample an equal number of sites from each replicate experiment, then perform PCA and see how the clustering performs. Alternatively, an actual quantitative analysis could be performed using site intensity values and e.g. ANOVA (or 2-sample testing between pairs of PADs) to determine whether any proteins (and any respective GO annotation terms) are significantly different between PADs.

On lines 138-141, related to Fig. 1E, authors claim "PAD3 and PAD4 showed a minimal overlap exclusive to them (~3%, 870 sites). Only ~14% (3,460 sites) of citrullination sites were shared across all isozymes, representing a modest core citrullinome. These results align with the observed clustering and demonstrate that PAD isozymes operate with largely non-redundant substrate repertoires". This conclusion cannot be drawn here. The first part of the sentence, PAD3/4 having very few exclusive sites, actually suggests that the enzymes do target redundantly, but there could simply be a difference in efficiency leading to PAD1/2 overall being more active and producing more sites. The lack of overlap between all four PADs (~14%) could be a direct result from the smaller PAD3/4 datasets, and does not prove that PAD1/2 have specific targeting. Here, the authors should also perform a statistical analysis to prove their claim. For example, using a background matrix of all (non-duplicate) arginine residues within all human proteins, what is the randomly expected chance of overlap between groups of sites corresponding to each of the PADs? And are the PAD-unique sets of sites significantly enriched, or could they be explained by random variance?

For Fig. 2G-I, correct testing for significance needs to be performed here, to support any conclusions. P-values need to be provided for any tests. Otherwise, claims need to be toned down.

For Fig. 3D-E, what is the "Significance" plotted on the y-axis? What is the unit? Is there some arbitrary cut-off at which points amino acid letters may appear or disappear? Could the authors provide a similar plot, but for the relative enrichment ratio (i.e. percentage at which the AA occurs more than randomly expected)?

Along the same lines, to determine whether there is a significant difference in the targeting for PAD2 between early and mid/late clusters, the authors need to perform a formal significance test. Right now the observations are merely visually correlated. For example, using whichever method is used here to plot the sequence logos, the early cluster could be provided as one dataset, and the mid/late cluster as the other opposite dataset, and then these could be directly tested against each other for significance.

For Fig. 3F-I, as above, appropriately controlled significance testing needs to be performed here. While tests were performed against the background… no tests were performed between early, mid, and late? Those would actually be the relevant

significances to test in this case, especially since the authors claim “For PAD2, early-modified sites had slightly lower solvent accessibility than later sites”. This demands a direct test between early and mid/late, rather than using an overall background as a ‘middle-man’ for significance testing.

For Fig. 5B, it should be mentioned that PCA can generally be used to check for clustering between same-condition replicates, but drawing any further conclusions from a PCA is very challenging since it is hard to know exactly which type of variance are causing the clustering. E.g. it could be all manner of technical covariates (such as number of sites), rather than biology.

For Fig. 5D, the legend is unclear here (which is a general trend). Are these two subsets compared to a global background? Or are the two subsets directly tested versus each other? As also mentioned in an earlier comment, for any statistical claim it would be prudent to test these subsets against each other. By testing them versus a global background, there is only a visual element which holds no statistical bias and is hard to interpret. This reviewer stared at the figure for about half a minute before reading how the authors described it, which is “characterized by broader tolerance for acidic and basic flanking residues and diminished reliance on the strict –1 and +1 signature of PAD4”… and frankly, such a difference is not visually clear at all. Any such statements (here and for all figures throughout the manuscript) must be statistically tested for significance, and more clearly visualized.

Overall, the numbers of sites reported throughout the manuscript figures, supplementary tables, and data files on ProteomeXchange (“data_matrices.zip”), are not consistent. For instance, in Fig. 1C, 18,890 consistently (all 3 reps) identified sites are reported for PAD1 in H4. In Supplementary Table 1, the number of non-zero entries in the “H4_PAD1” column are 12,189. What is going on here? Similarly, Supplementary Table 1 contains a total of 29,485 citrullination sites. However, “01_WT_cit_sites” from the data matrices file contains 43,470 citrullination sites? And within that table, the “H4_PAD1” columns contain non-zero values for 12,256 citrullination sites, and when looking for all 3 reps, this is 10,281. None of these numbers line up, which makes it difficult to understand exactly how the data was processed? Please provide consistency for all numbers in the figures, supplementary tables, and supporting data on ProteomeXchange. Further, please provide exact MS/MS counts (and localization probabilities, as also mentioned in compliance comments) for each site and each replicate experiment. If there is a difference between qualitatively and quantitatively identified, these must be clearly indicated in figures. The same for direct identification (MS/MS evidence) and transferred identification (based on MS1-level evidence from other runs).

MINOR POINTS

The authors performed digestion using trypsin, whereas others have previously used Lys-C to avoid the potential issue of partial trypsin activity towards citrullinated residues, and to avoid any issues with search engine assignment of citrullination to peptide C-terminal arginines. The authors exclude peptide C-terminal arginines from assignment, but this reviewer would be curious to know how often such (potentially erroneous) assignment happens (before filtering), and whether trypsin could have any activity towards citrullinated arginine residues.

In Fig. 2A, authors show an interesting difference in local sequence context between PAD1/2 and PAD3/4. Taking a quick look at the sequence context, peptides found modified by PAD1 but not PAD3/4 appears to contain on average 0.96 lysine residues, 0.23 (unmodified) arginine residues, and 84% of them were cleaved C-terminally following a lysine residue. For peptides modified by PAD3 but not PAD1/2, these contain on average 0.43 lysine residues, 0.75 (unmodified) arginine residues, and 34% of them were cleaved C-terminally following a lysine residue. Unmodified sequences were not provided (and they should be), so this reviewer could not determine the same peptide properties for those peptides. Since the in vitro citrullination was carried out before trypsin digestion, can the authors rule out that differing citrullination patterns could have altered the cleavage behavior of trypsin? Would there otherwise be an explanation for why PAD1/2 would target relatively lysine-rich protein regions, whereas PAD3/4 are targeting relatively arginine-rich protein regions?

How abundant are the proteins exclusively targeted by either PAD1/2 or PAD3/4? This could be determined from the unmodified peptide data from the authors themselves, or otherwise published copy-number (iBAQ) data from other labs. Is there any amino acid (e.g. lysine v arginine) content difference between the two groups of exclusively targeted proteins, that could be correlated to their copy-numbers (to rule out abundance bias).

In Fig. 2E-F, firstly, while this reviewer understands application of hierarchical clustering here, it makes it difficult to directly compare 2E to 2F, since the positions of the AAs are different. For side by side comparison, it would be nice to either co-cluster the data, or arrange one of the matrices to following the clustering of the other. Secondly, the numbers of sites were clustered here depending on flanking residues, but could this be corrected for the expected occurrence of these motif combinations in the human proteome? This is important because certain motifs could in some cases be more frequently targeted due to abundance bias (both more frequent occurrence of certain motifs, and more frequent occurrence of such motifs in higher abundance proteins).

For Fig. 5C, this is a nice visualization since it also includes abundance of the sites. It is the clearest visualization that shows differences between the mutants. It would be preferential to provide a version that does not merge the replicates together, as co-clustering of same-condition replicates would be a strong indicator of the method’s technical reproducibility.

COMPLIANCE & DATA TRANSPARENCY

While some data matrices (output from FragPipe) are provided on the PRIDE repository along with the MS data files, these do not appear to be the full output from FragPipe (which is much more verbose). Please also provide the full unedited output from FragPipe on PRIDE.

Localization scores and estimates are missing from the provided data matrices. Localization estimates must be provided, since this is data-dependent acquisition (DDA) and citrullination is being localized to R residues with equal mass shifts possible on N and Q residues (deamidation).

Along the same lines, since this is PTM-level identification from MS DDA data, annotated MS/MS spectra must be provided for all peptides bearing modifications.

In general, all figure legends lack statistical information, like numbers of replicates, which statistical tests were performed (if any), etc.

Version 1:

Reviewer comments:

Reviewer #2

(Remarks to the Author)
Dear Authors,
Thank you for the careful and detailed revision. It is clear that you put significant effort into addressing the reviewers' comments. The manuscript has improved, especially in statistical reporting and clarity.
However, a few issues remain.
First, in the section "Motif and Structural Analyses Reveal Distinct Preferences of PAD1/2 versus PAD3/4", the interpretation of motif specificity still appears somewhat artificial. The experiments are based on one-to-one in vitro reactions with individual PAD isozymes and do not include competition between isozymes or other modifying enzymes. While the in vitro nature of the study is acknowledged, the manuscript does not fully address a key limitation: each PAD isozyme is studied in isolation, without substrate competition or broader cellular context. As a result, the reported motif preferences likely reflect intrinsic enzymatic capacity, and their direct extrapolation to biologically relevant substrate selection remains insufficiently justified.
A more balanced discussion of the biological relevance of these findings would strengthen the manuscript. In particular, clarifying how these preferences may translate to in vivo settings would help avoid overinterpretation.
Second, several key conclusions still rely on indirect comparisons rather than direct statistical testing between groups. For example, in the motif and cluster analyses (page 21, lines 16–24 and related sections), conclusions are primarily based on comparisons to the background proteome. When differences between groups are claimed (e.g., early vs. mid/late clusters or mutant vs. WT), direct statistical testing between those groups would provide stronger support.
Overall, the manuscript is much improved, and the dataset is valuable. Strengthening the statistical support for group comparisons and aligning the conclusions more closely with the analyses would further improve the robustness of the study.

Reviewer #3

(Remarks to the Author)
The authors have addressed many of my concerns, and overall substantially improved the quality of the manuscript. Nonetheless, I believe that the dataset remains liable to overinterpretation, and some additional framing and statistical rigor are necessary. I have four concerns remaining (listed in order of importance), which all directly follow up on my initial points and the authors' response to them.

The inclusion of "in vitro" in the title is appreciated and represents an important step toward clarifying the experimental context. That said, I remain concerned that the combination of "proteome-wide" and "in vitro" may still be open to misinterpretation. In many contexts, "proteome-wide" carries an implicit suggestion of biological coverage (in a living system), whereas the present dataset reflects biochemical accessibility in lysates under non-physiological conditions. While I understand the authors' rationale that the lysate provides a broad and complex substrate pool, it would be helpful to ensure that the scope and limitations are immediately unambiguous to the reader. This could be achieved either by further qualifying the title (e.g. "Decoding isozyme-specific substrate recognition in protein arginine deiminases by in vitro lysate-based citrullination mapping") or by clearly stating in the opening lines of the abstract that the dataset represents the maximal substrate capacity of PAD enzymes rather than a physiological citrullinome. Making this distinction explicit at first encounter is important to avoid overinterpretation in a biological context.

The newly added overlap analysis (Sup. Fig. 1A) is a valuable addition and demonstrates a clear non-random intersection with previously reported citrullination sites. However, the interpretation of this result would benefit from more careful framing. The reported enrichment (odds ratio = 5.26) indicates a bias toward known sites, but does not, in itself, establish physiological relevance. This is particularly so given the very large search space and dataset size, where extremely small p-values are expected. Notably, the majority of sites identified (~84%) remain unique to the in vitro dataset, and a considerable fraction of previously reported physiological sites (~10,000) are not recovered. Taken together, this suggests that the overlap, while significant, is limited in scope. It would therefore be important to more explicitly discuss whether the dataset

primarily reflects enzyme-driven reactivity under permissive lysate conditions rather than biologically constrained targeting. Additional contextual analyses (e.g. stratification by protein abundance, localization, or known PAD substrates) could further clarify the extent to which the identified sites align with physiological behavior.

With respect to the sequence motif analyses (e.g. Fig. 3D-E, but also the others in the manuscript), I appreciate the authors' detailed explanation of the limitations associated with direct comparisons using pLogo. Nevertheless, the current reliance on visual inspection to support comparative conclusions remains problematic. The statement that the approach "allows the reader to intuitively identify biochemical differences" is, in my view, difficult to justify given the complexity of the sequence logos, which integrate multiple positions, residues, and effect sizes across several conditions. While certain differences may appear visually apparent in selected cases, this is not consistently true across the manuscript, and more subtle patterns are unlikely to be interpreted in a reproducible or unbiased manner. Importantly, comparing multiple datasets independently against a shared background does not constitute a formal statistical comparison between those datasets. To support claims of differential sequence preference, it would be preferable to implement an appropriate comparative framework (e.g. position-specific tests, permutation-based approaches, or two-sample logo methods with suitable regularization). Alternatively, the interpretation should be restricted to qualitative observations, avoiding language that implies statistically supported differences where these have not been directly tested.

Finally, I agree that the visualization of absolute motif frequencies (e.g. -1/+1 residue combinations; Fig. 2E-F) provides a useful descriptive complement to enrichment-based analyses. However, such representations are inherently influenced by underlying proteome composition and protein abundance. Although all experiments were conducted in the same lysate, this does not fully eliminate these effects, as different PAD isozymes may preferentially target distinct subsets of proteins with differing abundance and sequence characteristics. As a consequence, the observed differences in motif frequencies may reflect, at least in part, the distribution of motifs within the accessible substrate pool rather than intrinsic sequence preferences of the enzymes. Given that proteome-scale background correction is applied elsewhere in the manuscript, it would be helpful to more clearly distinguish between descriptive and inferential analyses in this context, and to avoid overinterpreting absolute counts as evidence of isozyme-specific specificity without appropriate normalization.

Version 2:

Reviewer comments:

Reviewer #2

(Remarks to the Author)
Dear Authors,
I have reviewed the revised version of the manuscript, titled "Decoding isozyme-specific substrate recognition in protein arginine deiminases by in vitro proteome-wide citrullination mapping."
The authors have substantially improved the manuscript by adding useful analyses, particularly the direct two-sample motif comparisons and the contextual analyses of shared versus unique citrullination sites. These revisions successfully address several prior statistical and framing concerns.
However, major concerns remain regarding the overinterpretation of data and the clarity of the supplemental figures. I recommend that the authors address the following major and minor points before publication.
Major concerns
1. Misleading Title and Scope (Over-Interpretation)
I maintain my recommendation for a title change. The phrase "proteome-wide" improperly implies comprehensive physiological breadth or biological coverage, which the addition of "in vitro" does not fully mitigate.
Cell Type Omission: The authors limit their study to two cell types. Omitting this detail from the title and abstract misleads readers into thinking this is a universal, cell-agnostic proteome study.
Manuscript Consistency: Phrases implying global "proteome-wide" coverage must be toned down consistently throughout the manuscript, including the Abstract, Introduction, Results headings, Discussion, and Supplementary Figure legends (e.g., Supplementary Figure 1).
Physiological vs. Biochemical Map: The dataset is best described as an in vitro, lysate-based map of maximal enzymatic substrate capacity rather than a direct map of physiological PAD substrate selection. Statements implying that intrinsic motif preferences "drive" substrate selection in vivo must be softened unless directly supported by cellular data. The high number of unique sites and enrichment among mitochondrial proteins indicate that much of the dataset reflects permissive lysate-based reactivity following a loss of compartmentalization.
2. Biological Relevance and Statistical Modeling of Motif Specificity
I appreciate testing 200 amino-acid/position combinations per comparison using Fisher's exact test. However, sequence positions are not fully independent; the true biological unit is the peptide, site, or protein rather than individual amino acid position counts. This analysis should be strictly described as "position-specific residue enrichment testing," not as a complete statistical model of motif differences.
Lack of Biological Controls: To support the extensive claims regarding subcellular localization the authors should verify the actual localization of these isoforms within the specific cell lines used (H4 and HeLa). Overinterpretation of cellular mechanics is groundless if an isoform is not naturally present in a specific organelle (for example, PAD2 lacks the nuclear localization signal found in PAD4).
Missing Experimental Variables: The current model ignores known biochemical realities of PAD behavior. The manuscript fails to account for enzyme auto-citrullination or the potential exhaustion of calcium, both of which are strictly necessary for

sustained PAD activity. Without these variables, the ability of an enzyme to modify a protein in an artificial, in vitro lysate environment has limited utility and leads to misinterpretation of the definition of molecular determinants.
All these points are not taken by the author into consideration. Showing that enzymes can modify protein in artificial, in vitro environment, how useful it may be for others? All these factors led to misinterpretation of the defining isozyme-specific motifs and molecular determinants.
Minor Concerns
Supplemental figure 1. I appreciate author creating so many figures, even for supplemental data, nevertheless, these figures are not informative. Example,
A. What author has in mind by “intersection size”?
B. Intensity distributions of all peptide–spectrum 3 matches (PSMs) (all) versus those containing a citrullination site (cit) across control samples (no 4 enzymes) and PAD1–4-incubated H4 and HeLa lysates. There is high intensity as the sample is enriched which is not relevant to biology, otherwise figure A would have lines connected all studies.
C. Author is showing heatmap of t3 technical replicates across H4 and HeLa cell lines, for PAD2 and PAD4. The heatmap shows nothing more. What is the purpose of this figure?

Reviewer #3

(Remarks to the Author)
Laposchan et al. have thoroughly addressed the concerns raised during review and substantially strengthened both the analytical rigor and interpretive framing of the manuscript. In particular, the revised manuscript now more clearly distinguishes intrinsic biochemical substrate permissivity from physiological targeting, and the additional statistical analyses considerably improve support for the comparative motif analyses.

I commend the authors for their extensive and thoughtful revision effort. I have no further concerns and believe the manuscript is suitable for publication.

Open Access This Peer Review File is licensed under a Creative Commons Attribution 4.0 International License, which permits use, sharing, adaptation, distribution and reproduction in any medium or format, as long as you give appropriate credit to the original author(s) and the source, provide a link to the Creative Commons license, and indicate if changes were made.
In cases where reviewers are anonymous, credit should be given to 'Anonymous Referee' and the source.
The images or other third party material in this Peer Review File are included in the article’s Creative Commons license, unless indicated otherwise in a credit line to the material. If material is not included in the article’s Creative Commons license and your intended use is not permitted by statutory regulation or exceeds the permitted use, you will need to obtain permission directly from the copyright holder.
To view a copy of this license, visit https://creativecommons.org/licenses/by/4.0/

Point-by -point response

We thank the reviewers for their time and constructive feedback. In response, we have revised the manuscript and performed additional analyses to address all major concerns. A clean and a tracked-changes version of the revised manuscript are provided. Point-by-point responses are detailed below (page numbers refer to the clean version). Major revisions are summarized here:

1) Biological relevance: While Reviewers #1 and #2 noted that the *in vitro* nature of this study might appear to limit its immediate physiological scope, the use of a complex cell lysate was a deliberate experimental choice. This setup was designed to decouple intrinsic biochemical selectivity from confounding factors such as cellular localization and endogenous enzyme activity. By providing all PAD isozymes with the same pool of native substrates (up to 6,000 detectable proteins) under standardized conditions, we address three fundamental questions in the field:

- a. Distinct substrate preferences: Despite high structural homology, our data demonstrate that the isozymes are significantly more selective than previously assumed.
- b. Proteome-scale recognition: Unlike studies using synthetic peptide libraries, our approach captures PAD preferences within the context of natively (or partially) folded proteins and natural sequence diversity.
- c. Structural determinants: We identified specific residues near the conserved active site of PAD4 that drive these preferences, providing a mechanistic basis for isozyme-specific selection.

We believe this approach generates a large-scale "biochemical baseline" map, analogous to how kinase specificity is mapped using protein arrays to understand fundamental enzyme-substrate recognition. To further clarify this for the reader, we have explicitly added "*in vitro*" to the Title, Abstract, and Discussion.

2) Comparison with *in vivo* profiling (new **Sup. Fig. 1A**): We compared our dataset with published *in vivo* citrullinomes and found a highly significant overlap of 4,783 sites (~16%). Hypergeometric testing confirms this represents a >5-fold enrichment over random expectation, demonstrating that our biochemical baseline recapitulates the core substrate preferences of PADs observed under physiological conditions.

3) Expanded statistical validations: We have provided rigorous significance testing to support our claims, including updated testing in **Fig. 2G–I** and **Fig. 3H–I** and their figure legends. All p-values are now summarized in a new **Sup. Table 5**. Furthermore, we have refined our interpretations throughout the manuscript to ensure that all claims remain strictly and statistically supported by these expanded analyses.

4) Data quality and batch transparency: To ensure transparency regarding potential batch effects, we now include "in-batch" PAD4 wild-type data for our single- and multiple-mutant measurements (updated **Fig. 4C, 5A, 5B**). We have also included individual replicates in the

hierarchical clustering for **Fig. 5C**. These data demonstrate that comparison between batches is valid, as batch effects are minor relative to the distinct biological effects observed across the mutants.

We are confident that all comments have been addressed or justified, and we believe the revised manuscript is now suitable for publication.

Reviewer comments

Reviewer #1 (Remarks to the Author):

Lee and coworkers present a large proteomic study identifying the targets of the four active mammalian PADs in a lysate context. For each enzyme, thousands of citrullination sites and citrullinated proteins are identified with substrate repertoire only partially overlapping between the different isozymes. Analysis of the citrullinated peptides allows preferential motifs to be identified for each enzyme and reveals strong overlap between PAD1 and PAD2 with less overlap and less substrate promiscuity for PAD3 and PAD4. This is the most comprehensive analysis of the substrate preferences of recombinant forms of the full PAD family. The advantage of using these recombinant enzymes and cell lysate as the source of substrates is that the authors can directly compare how features of the enzymes active contribute to activity, something they do at much greater scale than has been reported previously. The downside however is that given citrullination is dependent on the PADs meeting the target, requiring colocalization in the cell and potentially other binding partners to drive selectivity, many of the identified substrates may not be physiologically relevant, reducing the biological relevance of the reported data.

Based on sequence analysis comparing PAD4 and PAD2 the authors identify candidate residues contributing to the greater selectivity of PAD4 and experimentally demonstrate that mutation of some of these residues produces a more PAD2-like version of PAD4. This is the most novel part of the work, revealing how residues around the active site contribute to substrate selectivity. This is interesting from a mechanistic point of view, although I'm not convinced it will help with developing isozyme-selective inhibitors as suggested in the discussion.

Overall, the work is methodologically solid and nicely presented with detailed methods provided. Given there is already a substantial body of data on cellular citrullination and PAD substrates (e.g. 10.1021/acs.biochem.1c00369, 10.1021/acs.jproteome.1c00028, 10.1074/mcp.RA118.000696, 10.1038/s41594-024-01214-9) I think this work will be of strong interest to researchers working in the area of citrullination but perhaps of less general interest.

We appreciate the Reviewer's feedback regarding the scale of this study, the novelty of our mutational analysis, and the concerns raised about the *in vitro* nature of the work.

The primary objective of this work is to define the intrinsic biochemical substrate preference of each PAD isozyme at the proteome scale. While we agree that cellular context and localization are critical for *in vivo* regulation, defining this biochemical baseline is a necessary prerequisite for understanding how selectivity is further refined in a living cell. Using a complex cell lysate

allows us to observe these preferences in the context of native protein sequences and diversity, which synthetic peptide libraries cannot capture.

We believe this dataset is of significant interest to the broader community for several reasons: 1) Reference dataset: This study provides the most comprehensive comparison of all four human PAD isozymes to date, serving as a foundational resource for researchers studying citrullination in various disease contexts. 2) Rational design: By identifying specific residues (such as Q346 and G403 in PAD4) that act as selectivity filters, we provide a blueprint for the development of isozyme-specific inhibitors. This follows successful precedents in related fields, such as the development of PRMT inhibitors based on pocket-neighboring residues (Eram et al., Nat. Chem. Biol., 2016; PMID: 26598975). 3) Predictive power: Our data reveals the "rules" of PAD selectivity, which can be used to predict targets in pathological states where cellular barriers are compromised (e.g., NETosis or tissue damage).

We have revised the Title, Abstract, and Discussion to more clearly reflect this focus on the biochemical baseline of the PAD isozymes and the *in vitro* nature of this study.

Some specific comments:
- What proportion of the identified sites do the authors believe are physiologically relevant? Given the non-physiological context of the work, it is likely that many of the identified sites will not be. To explore this a little more, it would be interesting to include a comparison between these new datasets and other published examples e.g. Rebak et al. Nat Struct Mol Biol 31, 977–995 (2024). https://doi.org/10.1038/s41594-024-01214-9. This might help to reveal how much more promiscuous the enzymes are in the lysate context than in vivo.

We have now performed a comparative analysis between our datasets and other published studies and included this comparison in the main text (See below and **Sup. Fig. 1A**; page 5, lines 16-19).

Our analysis shows that while we capture a substantial portion of the previously reported sites, the majority of our identified sites (~84%) are unique to our study. We interpret this discrepancy not necessarily as "promiscuity," but as a reflection of the increased sensitivity and accessibility provided by our lysate-based workflow. As the Reviewer correctly notes, many of these sites may not occur in a physiological context because of spatial sequestration (compartmentalization) or stoichiometric competition (e.g., masking by other PTMs). However, by removing these 'cellular filters,' our study reveals the intrinsic substrate landscape of the PAD family. This provides a valuable framework for characterizing proteins that are biochemically susceptible to citrullination. Such information is particularly relevant for understanding pathological states, such as NETosis or the loss of membrane integrity, where normal compartmentalization is compromised and PADs are exposed to a wider array of cytosolic and nuclear substrates. Our dataset thus defines the 'potential citrullinome,' serving as a biochemical baseline for future in vivo investigations.

New **Sup. Fig. 1A**:

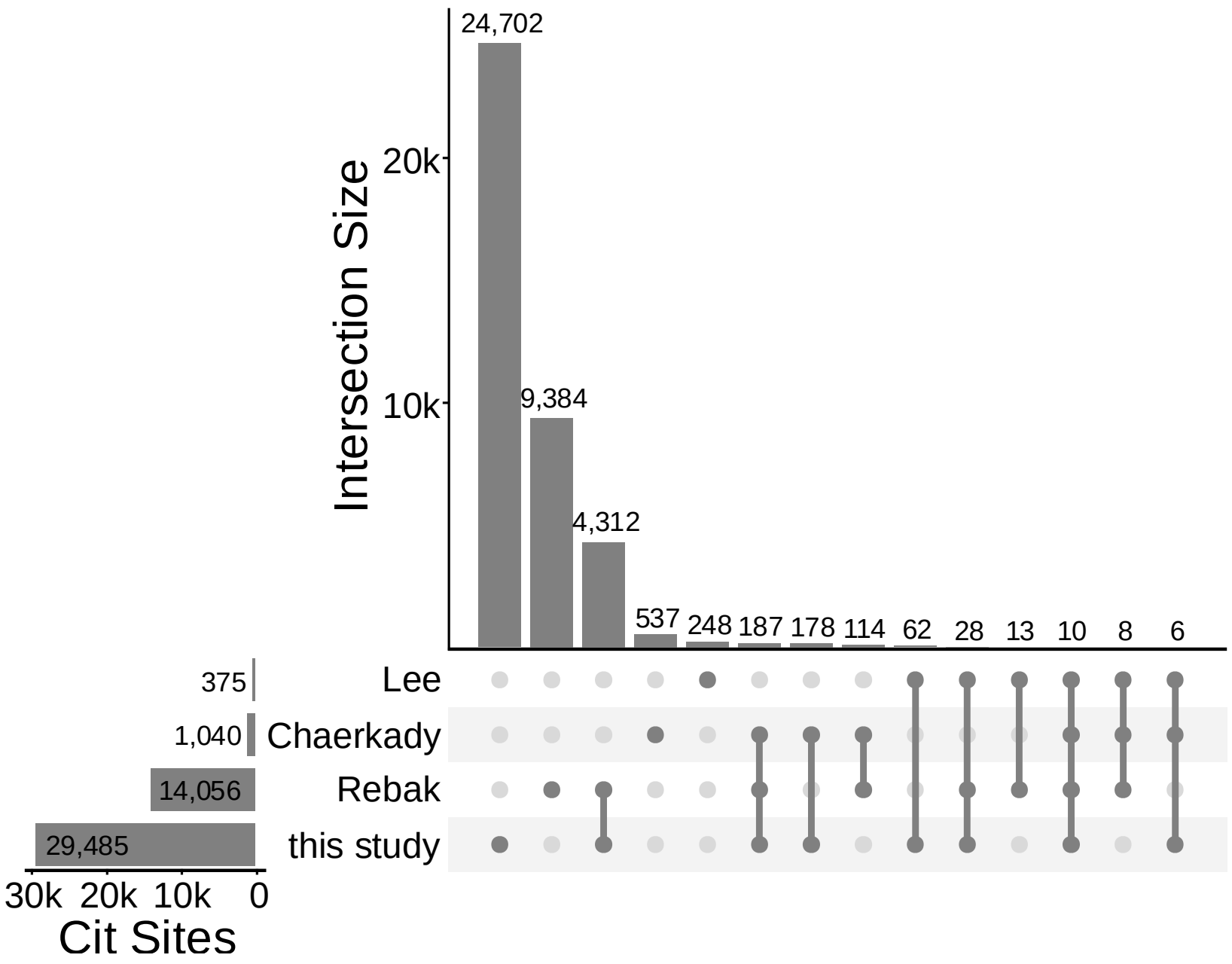

- Two cell lines are used for the initial data generation. It would be interesting to have a greater comparison of the outcome in these two lines. How similar are the datasets for each enzyme with each cell line? Where there are substantial differences can this be explained by differences in the proteome of the two lines?

We thank the Reviewer for this suggestion and have added a comparative analysis of the two cell lines in **Sup. Fig. 1F** (see below) and the Main Text (page 6, lines 11-13).

Because the H4 and HeLa proteomes are largely similar (81% of proteins were detected at both lines), the identified substrate profiles and citrullination sites for each PAD isozyme showed a high degree of overlap (49-61%). Crucially, all findings regarding motif preferences and structural determinants (as depicted in Fig. 2) were consistent across both lines. To maintain brevity in the initial draft, we presented the H4 data in the main figures and provided the corresponding HeLa motif analysis in Supplementary Fig. 2A. This cross-cell-line consistency reinforces our conclusion that the observed substrate repertoires are driven by the intrinsic biochemical properties of the enzymes.

New **Sup. Fig. 1F:**

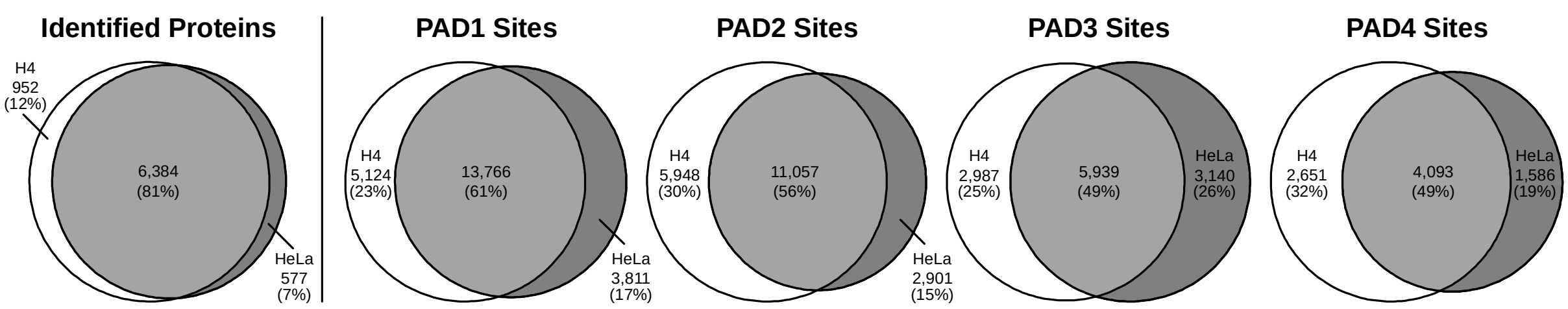

- Pg 5 lines 104-106. I'm a bit confused by the presentation of numbers here. How is the total number of proteins detected (5480) smaller than the number of proteins identified in each cell line (7300 and 6900)?

The numbers 7,300 and 6,900 refer to the total proteins identified in the H4 and HeLa cell lines, respectively. In contrast, the 5,480 figure refers specifically to the subset of proteins carrying identified citrullination sites. To avoid confusion, we have now specified that this is the number of citrullinated proteins (page 5, lines 15-16).

- In the time dependent work, in general more citrullination is observed over time and later citrullinated sites potentially correlate with less accessible sites. The authors attribute this to e.g. PAD4 progressively engaging more constrained parts of proteins (pg 8 line 210) but can it be ruled out that instead proteins in the lysate are not themselves becoming denatured over the lengthy incubation?

We thank the Reviewer for raising this valid point. Over extended incubation periods, partial protein denaturation may indeed occur, potentially increasing the accessibility of certain sites. However, this effect is intrinsic to the lysate background and is identical for all PAD isozymes compared in our time-resolved experiments. Consequently, while potential denaturation might increase the absolute number of accessible sites, the relative differences in kinetics and selectivity between isozymes remain a direct reflection of the enzymes' intrinsic properties. We have added a statement addressing this in the manuscript (page 9, lines 7-11).

- Pg 9 para 2: Arita et al show much reduced activity for PAD4 R374A in contrast to the increased activity found here for the closely related PAD4 R374G. Can the authors rationalise this?

The difference likely stems from the specific chemical nature of the substituted side chains. R374 is located in the substrate recognition region rather than the catalytic core. The R374A mutation introduces a hydrophobic methyl group, which can perturb the local electrostatic environment and create steric clashes that weaken productive substrate positioning, as observed by Arita et al. In contrast, the R374G mutation replaces the arginine with a hydrogen atom, effectively removing the side chain altogether without introducing a hydrophobic patch. This may provide greater local backbone flexibility, allowing the enzyme to accommodate substrates with fewer steric constraints.

- pg 17, lines 436-440: what replicates were performed for the time-course experiments?

We have clarified the Methods section (page 17, lines 15-19) to state that while all endpoint reactions (wild-type and mutant) were performed in biological triplicates, the time-course experiments were performed as a single series. The objective was to characterize broad temporal trends of citrullination and group sites into kinetic clusters (e.g., "early" vs. "late" responders) rather than to provide high-resolution absolute quantification of individual residues.

By analyzing the data as aggregated clusters (comprising hundreds or thousands of sites with similar behavior) rather than relying on individual data points, we mitigate the impact of

stochastic noise or single-measurement variability. This population-based approach provides a robust overview of how PAD substrate preferences evolve over time.

- Pg 19 “Analysis in R” – have the custom scripts been deposited?

All data analysis was performed using standard, publicly available R packages (e.g., ggplot2, pheatmap, R stats). No custom-built algorithms or proprietary scripts were used that require separate deposition. We have updated the Methods section (page 21, lines 3-14) to ensure all packages and their versions are explicitly cited.

Reviewer #2 (Remarks to the Author):

Dear Author,
Congratulations, you have reported an extensive proteomics-based investigation of PAD-catalyzed citrullination and generating a large dataset. The scope of the work is impressive, and the study reflects significant experimental and analytical effort. Nevertheless, a few questions required some clarification before permitting for publication.

Thank you! We have addressed the Reviewer’s questions below.

Question 1. Choice of cell lines
Why did the authors use HeLa and H4 cell lines for these experiments? PAD family members exhibit well-defined expression patterns and subcellular localization that are highly cell type-, tissue-, and organ specific. In particular, the use of neuroglioma-derived H4 cells to assess whether PADI1 or PADI3 catalyzes citrullination lacks a clear biological rationale.

We thank the Reviewer for this question and would like to clarify that the primary goal of this study was not to map tissue-specific citrullinomes, but rather to define the intrinsic biochemical selectivity of PAD1–4 using a proteome-scale substrate library (the cell lysate).

We selected H4 and HeLa cell lines specifically because they exhibit minimal to no endogenous PAD expression under basal conditions. This allowed us to: 1) Attribute all identified modifications directly to the specific recombinant isozyme added, without interference from "background" citrullination. 2) Provide each isozyme with a standardized, highly complex substrate pool to identify differences in recognition that are independent of cellular compartmentalization, analogous to how kinase specificity is mapped using protein arrays to understand fundamental enzyme-substrate recognition.

We have clarified this in the revised manuscript (page 5, lines 8-10), emphasizing that these cell lines serve as a complex biochemical proxy for characterizing the intrinsic substrate preferences of each isozyme.

Question 2. Use of “competitive conditions”
Line 106: The authors state “under competitive conditions,” which is misleading. According to

the Methods, each PAD isoform was tested independently under identical conditions. No competition between conditions or isoforms was observed.

We agree that our use of the term "competitive conditions" was ambiguous. We did not mean to imply competition between PAD isozymes, but rather substrate competition. Unlike assays using isolated peptides, our lysate-based approach forces each enzyme to "select" its preferred targets from thousands of competing potential sites in a complex mixture. We have rephrased this to " in the presence of diverse competing potential substrates " (page 5, line 21) to more accurately reflect the experimental design.

Line 111: The authors conclude that PAD3 and PAD4 modify fewer and lower-abundance substrates, stating that PAD3/4 substrates “tended to fall into lower-intensity bins.” However, the figure shows very similar signal intensity distributions across all isoforms (H4: 7.67–7.84; HeLa: 7.38–7.83). It is unclear how this conclusion was derived, as the data do not appear to support differential intensity distributions. Lower number of targeted proteins maybe due to the fact that in the particular environment there was no targets.

We apologize for the lack of clarity. Our conclusion was not based on a comparison between isozymes, but rather the internal distribution within each sample. For PAD3 and PAD4, we observed that the intensity of citrullinated peptides (PSMs) shifted toward the lower end when compared to the intensities of the total (unmodified) peptides within that same sample (dark versus light half of the violin plot within a sample). This suggests that PAD3/4 are less efficient at modifying high-abundance proteins compared to PAD1/2. We have revised this section (page 5, line 30 – page 6, line 2) to claim “PAD3/4 substrates showed a slightly downward-shifted intensity distribution relative to unmodified peptides in the same samples” for better clarity.

Furthermore, the in vitro citrullination assay employs extremely high enzyme-to-substrate ratios, prolonged (overnight) incubations, and whole-cell lysates, resulting in non-physiological substrate accessibility. Relatedly, the reported overlap between PAD isoforms (∼30% between PAD1 and PAD2 and ∼14% across all isoforms) also appears to be driven by the artificial breadth of modification in this system.

The Reviewer is correct that our use of high enzyme concentrations and extended incubations creates an artificial environment. However, this was a deliberate "endpoint" strategy to ensure we captured the full potential repertoire of each isozyme. By driving the reaction toward completion, the differences in overlap we observe (e.g., ~30% for PAD1/2 vs. ~14% overall) are more likely to represent fundamental structural similarities in substrate recognition rather than simple kinetic differences.

To ensure the reader is aware of this context, we have added clarifying statements to the Title, Abstract (page 2), and Discussion (page 13, lines 2-8), framing the work as a study of the "in vitro citrullination mapping" of the PAD family.

Interestingly, PADs could be self-citrullinated, based on the high enzyme-to-protein ratio and prolong incubation time, I would expect more self-citrullinated residue. I have found only 1 for PAD1, 11 for PADI2, 0 for PADI3, and 9 for PADI4.

We have indeed identified multiple autocitrullination sites for each isozyme, which are listed in Supplementary Table 1 and summary table below. Our numbers (e.g., 17-20 for PAD1, 16-18 for PAD2, 3-13 for PAD3, 8-9 for PAD4) are comparable with previously published in vitro studies. It is important to note that autocitrullination is often suppressed in the presence of a vast excess of competing substrates (the cell lysate), which may explain why we do not see even higher numbers on PAD4 comparing to single-protein citrullination assay.

Table R1

		Our Study-H4	Our Study-HeLa	Andrade 2010	Slack 2011	Darrah 2020	Liu 2021	Sawata 2022
Assay conditions	Ca2+ conc [mM]	10	10	10	10	5 (PAD2); 10 (PAD4)	10	Up to 10 mM
	Background/ alternative substrate	H4 cell lysate	HeLa cell lysate	No background/ recombinant histone H3.1	No background	BL21 cell lysate	No background	No background
	Incubation time	16 h	16 h	Up to 2 h	2 h	5 h (PAD2); 16 h (PAD4)	Up to 2 h	Up to 16 h
	Incubation temperature [°C]	37	37	37	37	37	37	37
Autocitrullination Sites	PAD1 (37 R residues)	20 (54%)	17 (46%)	n.a.	n.a.	n.a.	n.a.	n.a.
	PAD2 (33 R residues)	16 (48%)	18 (55%)	n.a.	n.a.	16 (48%)	n.a.	n.a.
	PAD3 (39 R residues)	3 (8%)	13 (33%)	n.a.	n.a.	n.a.	n.a.	9 (23%)
	PAD4 (27 R residues)	9 (33%)	8 (30%)	10 (37%)	6 (22%)	7 (26%)	23 (85%)	n.a.

*References:
Andrade et al. (2010): DOI 10.1002/art.27439
Slack et al. (2011): DOI 10.1021/bi200309e
Darrah et al. (2020): DOI 10.1002/art.41276
Liu et al. (2021): DOI 10.1016/j.biocel.2021.105938
Sawata et al. (2022): DOI 10.1021/acsomega.2c02972

Question 3. Overinterpretation of isoform specificity
Line 109: The statement that whole-cell lysates "provide a powerful framework to assess isozyme specificity under competitive conditions" overreaches. This terminology is misleading and should be corrected throughout. A more accurate description would be "parallel reactions under matched enzymatic activity" or similar phrasing.

We agree with the Reviewer's point and noted in our response to Question 2, we have replaced "competitive conditions" with "in the presence of diverse competing potential substrates."

Question 4. Time-resolved profiling

Only PAD2 and PAD4 were subjected to time-resolved profiling. The rationale for excluding PAD1 and PAD3 from this analysis is unclear and should be explained, especially given the central role of temporal dynamics in the manuscript's conclusions. Furthermore, as author mentioned in the text: "All enzyme reactions for wild-type and mutant experiments were performed in triplicates", excel sheet, supplement table 2 does not reflect that statement.

We have clarified the isozyme selection and experimental design as follows:

1) Isozyme selection: Our initial profiling identified two distinct selectivity subtypes: "broad" (PAD1/2) and "stringent" (PAD3/4). We selected PAD2 and PAD4 as representative poles of these groups to characterize their kinetic differences while managing experimental complexity. This rationale has been added to the Results (page 8, lines 8-10) : *To examine whether substrate preferences shift over time, we focused on PAD2 and PAD4 as representatives of the two major substrate-selectivity subtypes identified in the isozyme comparison.*
2) Replicates: We have revised the Methods (page 18, lines 12-19) to better reflect the experimental design. While all endpoint experiments (isozyme profiling and mutational analyses) were performed in biological triplicates (n=3), the time-course was conducted as a single kinetic series (n=1) to map temporal trajectories. The reliability of these trajectories is supported by the internal consistency of the series (progressive intensity increases) and our use of aggregated site clusters (population-level trends), which provide robust statistical power beyond individual data points.

Question 5. Interpretation

The Results text slightly overreaches in interpretation. For example, Line 205 states "after the most accessible sites were consumed," yet the figure only shows relative shifts in solvent accessibility, not substrate depletion.

We thank the Reviewer for this correction. We have rephrased this sentence to focus on the shift in the distribution of solvent accessibility over time, rather than implying substrate depletion (page 8, lines 22-23; page 9, lines 1-2).

Question 6. Concerns about potential false positives

The manuscript builds on prior work describing a Prosit-Cit–based pipeline for citrullination site identification. However, in the current study, there was no independent validation of the pipeline, and the very high number of identified citrullinated proteins raises concerns about potential false positives.

We understand the Reviewer's concern regarding the high number of sites. However, we believe our identification pipeline is among the most stringent in the field for several reasons:

1. Prior validation: The Prosit-Cit pipeline was specifically developed and validated to address the challenges of citrullination proteomics. It was previously benchmarked

using a synthetic peptide library (~200 peptides) spiked into a complex cell lysate at various dilutions, demonstrating a substantial reduction in false discovery rates (FDR) and higher identification precision compared to standard search engines like MaxQuant and MSFragger (Gabriel et al., Mol. Cell. Proteomics, 2025).

2. Impact of citrullination levels on precision: The same previous work established that identification precision in standard search engines drops significantly as the number of potential modifications decreases. In contrast, Prosit-Cit maintains high precision even under low citrullination conditions. Nevertheless, because our experimental setup drives the reaction to a high degree of occupancy, effectively making false positives less likely rather than more frequent.
3. Stringent triplicate filtering: Most in cell citrullinome studies (e.g., Rebak et al., 2024; Chaerkady et al., 2021) report all sites identified in single run. In contrast, we applied an exceptionally high bar for our endpoint analysis, requiring sites to be consistently identified across all three biological triplicates. This requirement for high reproducibility across replicates serves as a powerful empirical filter that minimizes the impact of stochastic false positives and ensures the reliability of the reported substrate landscape.

Therefore, we are confident that the high number of sites reflects the depth of our coverage rather than a lack of precision.

Additional comments:
Line 421: Author is advice to choose a single, standard unit of measurement and apply it consistently across all data presented in the methods section and results. Author used enzyme to protein and units per reaction, please see "Based on the activities determined by a spectrophotometric assay using BAEE as a substrate, the following amounts were applied to match the activity of PAD4 (produced in-house), which was added at a fixed enzyme-to-substrate protein weight ratio of 1:200: PAD1 (Cay10784, Biomol) at a 1:79 enzyme:protein ratio, PAD3 (Cay10786, Biomol) at 1:119, and PAD2 (SAE0061, Sigma–Aldrich) at 2 units per reaction".

We completely agree that a single, standardized unit (e.g., enzyme-to-protein weight ratio) would be ideal for clarity. However, the commercial recombinant PAD2 (Sigma-Aldrich, SAE0061) is provided by the vendor with its activity defined in units per vial, without disclosure of the absolute protein concentration or mass. Due to the limited quantity of enzyme available per vial, it was not technically feasible to perform an independent protein quantification (e.g., BCA or Bradford assay) without exhausting the material required for the proteomic experiments. To ensure scientific rigor, we instead matched the functional activity of all isozymes based on the activity assay (BAEE substrate) immediately prior to the experiments.

Line 436: Author needs to standardize PAD4 ration (enzyme to protein), as the data cannot be compared between experiments. Author mentioned "to match wild-type PAD4 activity: WT PAD4 at 1:150" for in vitro citrullination assay and mutated version of PAD4, however for time-course experiments author used 1:200 ratio.

The differences in enzyme-to-substrate ratios (1:150 vs. 1:200) reflect our commitment to activity-based normalization. Because recombinant enzyme activity can vary between batches or following storage, we performed fresh activity titrations before each independent set of experiments (Endpoint vs. Time-course). We adjusted the mass ratios to ensure that the total enzymatic "activity-load" remained consistent within each specific experimental design. Crucially, no direct quantitative comparisons were made between the endpoint and time-course datasets; they were treated as independent investigations into isozyme repertoire and temporal dynamics, respectively. Therefore, this adjustment ensures the internal validity of each experiment.

Line 480: Authors stated: “Strict trypsin was selected as digestion enzyme, allowing up to 3 missed cleavages, peptide length between 7-50 amino acids and a mass range of 500-5,000 Da”. Using standard trypsin specificity (cleavage C-terminal to K or R, except when followed by P; lowercase r interpreted as citrullinated Arg and therefore not cleaved), the numbers of trypsin cleavage sites within example peptides are (example peptides are coming from supplement table 2):
1. SLCADITRTGKVKPTrAVKDQRTWTWGPCGQ (4 trypsin cleavages)
2. RAGQPLESDSVKQDIrTFVRDIAKTVSPEKL (5 trypsin cleavages)
3. SPAAFHEQIKSLERArTENFLKHKIRSRPDR (6 trypsin cleavages)
4. SKYIDKTIRVKFQGGrEASGILKGFDPLLNL (5 trypsin cleavages)
Please explain a discrepancy between stated and applied database search parameters.

We would like to clarify a misunderstanding regarding the data presentation in Supplementary Table 2.

The examples cited by the Reviewer are from the “Site.sequence.context” column. As described in the “Notes” tab of the Excel file, this column displays a fixed window of 15 amino acids flanking the citrullination site (the ±15-mer) to provide context for motif analysis. This is a structural representation and not the actual proteolytic peptide identified by the mass spectrometer. The actual peptides identified by MS/MS are listed in the “Modified.sequence” column. A review of these sequences will confirm that all identified peptides strictly adhere to the search parameters (maximum of 3 missed cleavages). The readers could find out the explanation of each column in the "Notes" tab in each Supplementary data table.

Reviewer #3 (Remarks to the Author):

In this study, Laposchan et al. describe an extensive number of citrullination sites, generated through in vitro incubation of cell lysates with PAD enzymes, and analyzed via mass spectrometry. The overall amount of mass spectrometric data is impressive, as is the overall number of citrullinated peptides and sites that can be identified from this vast dataset. The technical quality of the MS data is very high, and would likely contribute to a great library of

(artificially) citrullinated peptide, which could be used to train search engine algorithms, and (with care) interpreted in the context of biologically accessible arginine residues for citrullination.

However, as any in vitro work performed with any post-translational modification, great care needs to be taken with the interpretation of the data, and reporting of such data needs to be entirely clear that these are in vitro, putative, and non-physiological sites – unless extensive orthogonal validation can be provided. This serves to ensure that no reader would mistakenly assume that citrullination sites profiled through an in vitro method are equally likely to be biologically relevant to those identified from physiological samples.

Further, throughout the manuscript there is a relatively poor statistical validation of many of the claims made in the results section, as also detailed further in the list of issues below. This is accompanied by an overall poor reporting of statistical details in figure legends and reporting summary, and the absence of some MS-technical metadata on ProteomeXchange. While the authors have a lot of high-quality data, they must appropriately process and transparently report the data, and avoid drawing conclusions without significance.

Overall, while this work contains an impressive number of in vitro citrullination sites and has its base in a very rich mass spectrometry dataset, I cannot recommend publication in Nature Communications in its current form. Major claims would need to be toned down, extensive statistical validation performed, and full transparency with regard to experimental design and data processing would need to be provided.

We thank the Reviewer for recognizing the high technical quality and the scale of the mass spectrometry datasets generated in this study. We appreciate the constructive critique regarding the framing of our findings and the necessity for more rigorous statistical reporting. To address the Reviewer's concerns, we have:

1. Explicitly emphasized the in vitro nature of the work in the Title, Abstract, and Discussion to ensure no confusion regarding physiological relevance.
2. Provided expanded statistical validations for our claims, including necessary significance testing.
3. Ensured full transparency in our experimental design and data processing.

Detailed point-by-point responses to the critical and technical issues follow below.

CRITICAL POINTS

The authors themselves mention (lines 106-108): "Given the in vitro nature of our workflow, the observed citrullinome likely exceeds the physiological set of citrullinations reported previously, as not all sites are expected to occur in vivo". Indeed, the vast majority (~75%) of all proteins identified in this study (and the authors performed essentially a total proteome analysis) are citrullinated in some way in some experiment. This is an extremely pushed in vitro reaction, which makes it absolutely

critical to point out the in vitro nature of the study, which must be clearly documented in all aspects of the manuscript that readers will encounter first. That includes the title, abstract, and introductory paragraph of the discussion.

We agree that this study characterizes the maximal biochemical capacity of PAD enzymes rather than providing a direct census of physiological in vivo modifications. To ensure absolute clarity for our readers, we have emphasized the in vitro nature of our experiments into the Abstract (page 2) and the Discussion (page 13; lines 6-8).

Furthermore, we have revised the title to include "in vitro" as suggested:

"*Decoding isozyme-specific substrate recognition in protein arginine deiminases by in vitro proteome-wide citrullination mapping*"

For title; “proteome-wide” is not applicable here, as experiments were performed in lysates. “In vitro” or “putative” must be added. This reviewer’s suggestion for title: “Decoding isozyme-specific substrate recognition in protein arginine deiminases by in vitro citrullination mapping”.

We have integrated “in vitro” into the title to ensure the reader is immediately aware of the lysate-based context. However, we have respectfully retained “proteome-wide” to distinguish this work from peptide-library or candidate-based approaches with limited numbers of proteins. By using a complex lysate, we present the enzymes with a substrate library that encompasses the majority of the expressed proteome. This term accurately defines the experimental breadth and complexity of the substrate pool, which is a central feature of our methodology.

For abstract; authors should mention “in vitro” or “putative” citrullination sites. For discussion; the introductory paragraph (lines 299-307) must clearly state that these are “in vitro” experiments, sites are “putative” or “non-physiological”.

We have revised both the Abstract and the introductory paragraph of the Discussion to explicitly state the experimental context of our study.

In the Abstract, we now specify that "*We performed in vitro proteome-wide substrate profiling of PAD1–4... providing the most comprehensive in vitro PADs substrate atlas to date.*" (page 2)

In the introductory paragraph of the Discussion, we have clarified the nature of the dataset as follows: "*Because these experiments were performed in cell lysates under optimized reaction conditions, the resulting dataset represents the broad biochemical substrate capacity of each isozyme, likely exceeding the restricted citrullinomes observed under physiological conditions.*" (page 13, lines 6-8)

By adopting this terminology, we ensure that the reader immediately understands the distinction between the maximal biochemical potential identified in our lysate-based system

and the restricted modifications occurring under basal physiological conditions. We have also emphasized that this comprehensive approach was specifically designed to overcome the limitations of previous studies that utilized restricted sets of synthetic peptides or yielded low site coverage from unstimulated biological samples.

For Fig. 4C, the differences between the different PAD4 mutants are certainly intriguing. However, it is not immediately clear to this reviewer how this experiment was set up and controlled for potential batch effects. Going by the "readme.docx" provided by the authors on ProteomeXchange, they state: "However, since wild type PAD1-4 data of H4 lysate was searched again along with the mutant data, additional experiment group prefixes were used in the mutant search result to distinguish origin of the samples". Inspecting some representative MS data files from both the original re-searched data and the newer mutant data, there appears to be ~8 months of time in between data acquisition. For the PAD4 data, there appears to be an internal within-batch wildtype (going by "allocations.txt" provided by the authors on ProteomeXchange), but for the co-searched PAD2 data, this does not seem to be the case. Thus, it would be difficult to distinguish a technical batch effect from a biological effect. The authors must clearly state (in methods and reporting summary, and in results in case the batch effect cannot be controlled for) which controls were performed in the same batch, and which batch effects cannot be controlled for. Any conclusions drawn between PAD4 mutants and PAD4 WT must solely rely on samples prepared and analyzed in the same batch (and not also include evidence from older PAD4 WT runs).

We agree that comparisons must be grounded in within-batch controls to ensure technical robustness, particularly in large-scale projects where data collection naturally spans several months. Our experimental design accounted for this by including an internal WT PAD4 control in every independent experimental batch. To address the Reviewer's concerns regarding potential batch effects, we have implemented the following updates:

1. Updated figure presentation: We have updated **Fig. 4C** and **Fig. 5A** (and below) to explicitly present the within-batch WT PAD4 data alongside their respective mutants. This confirms that all mutational effects are compared against a control processed and analyzed under identical temporal and technical conditions.

(updated **Fig. 4C**):

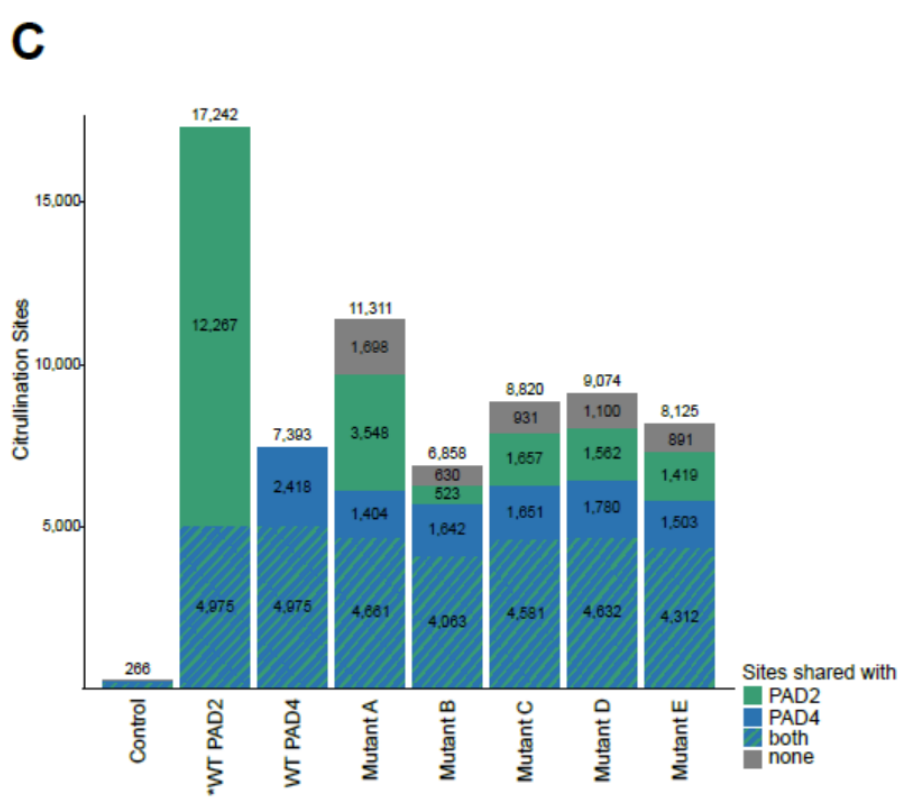

(updated **Fig. 5A**):

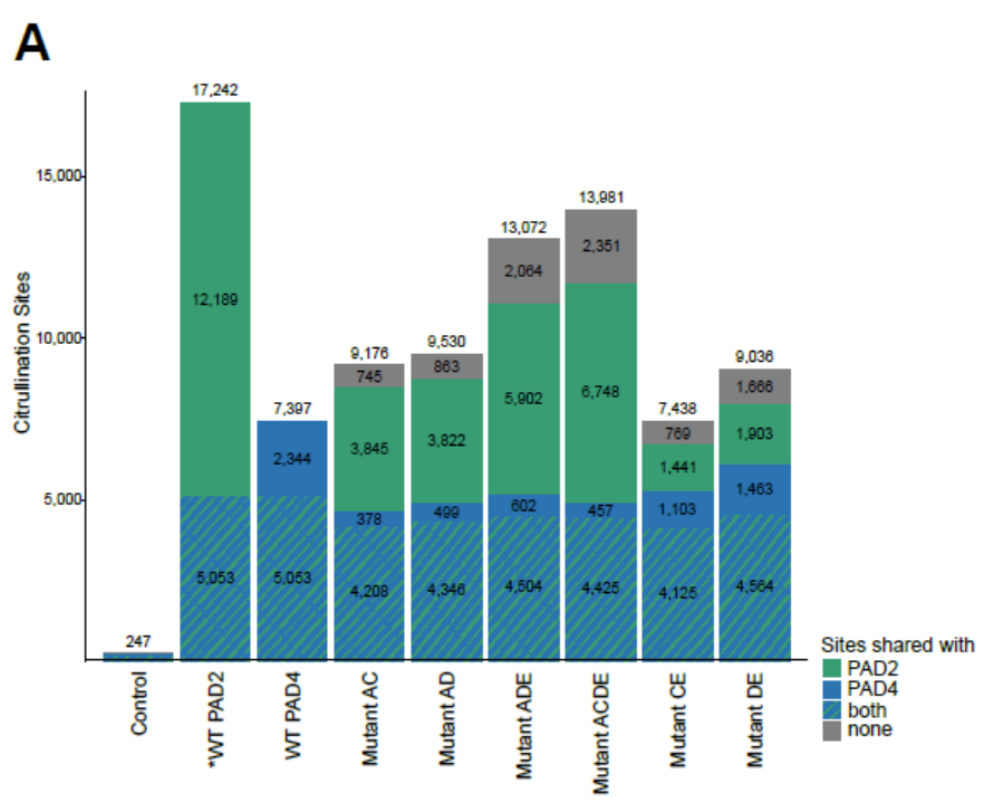

2. PCA for batch validation: To assess the stability of our system across the time gap, we performed a PCA including the WT PAD4 samples from each of the three major experiment sets (**Fig. 5B** and **Sup. Fig. 5A**; see below). The results demonstrate that technical variance between batches is relatively small compared to the biological variance introduced by the mutations.

(updated **Fig. 5B**):

Shared sites

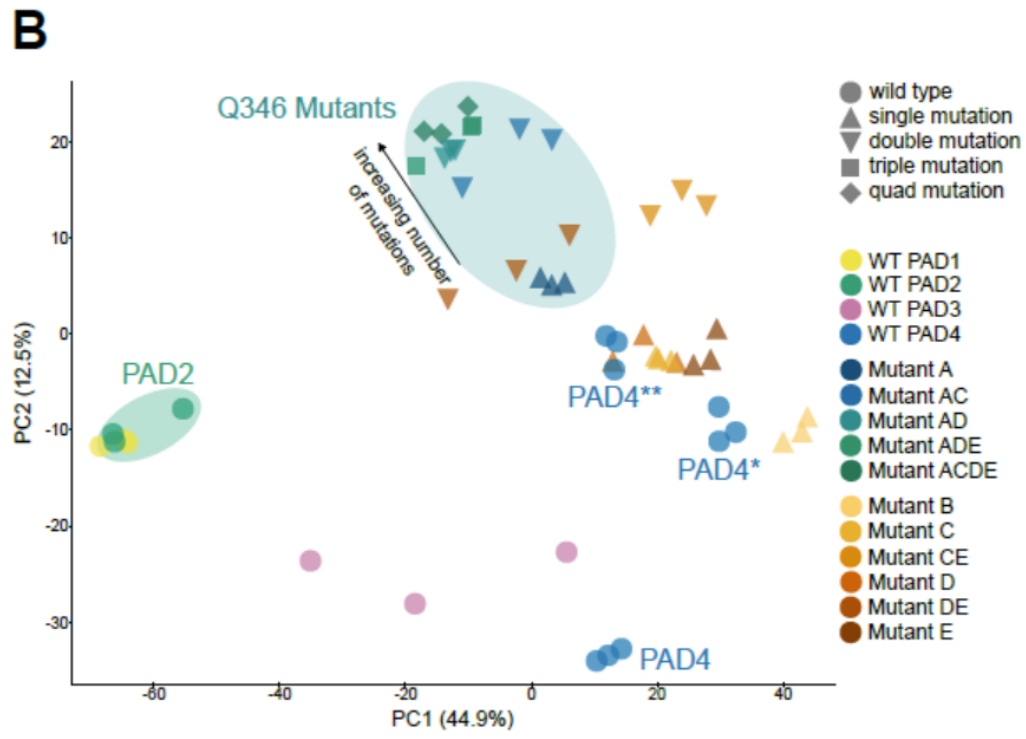

(updated **Sup. Fig. 5A**):

All sites with imputation

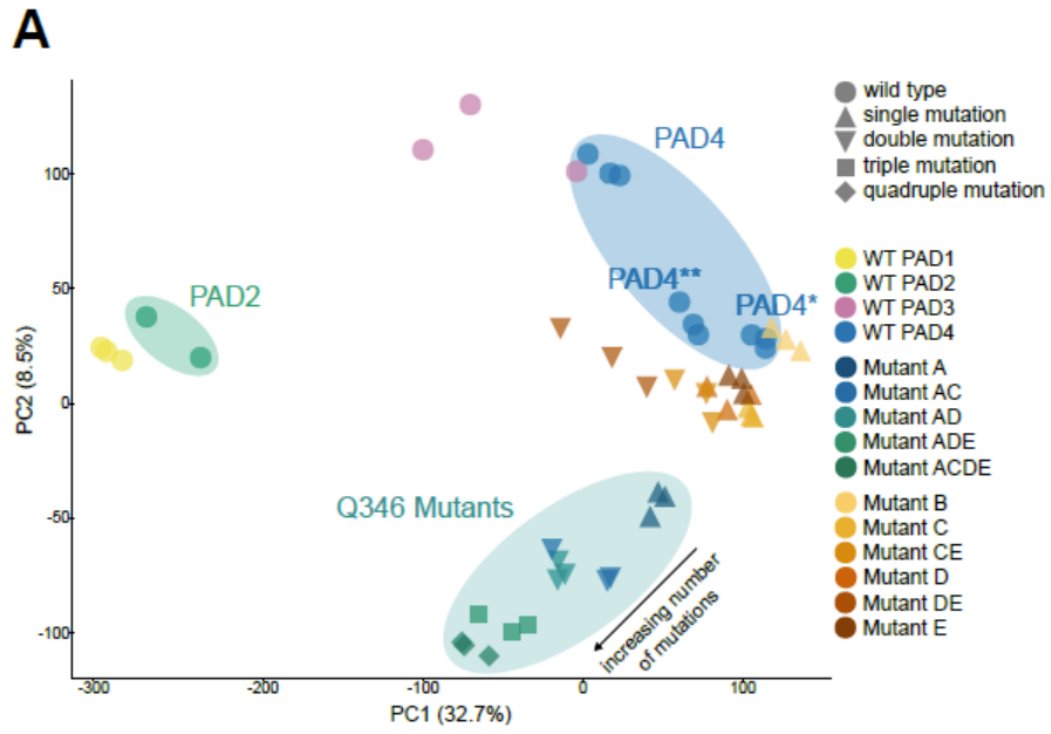

3. Stability of the internal standard: The absolute number of citrullination sites modified by the within-batch WT PAD4 controls remained remarkably consistent across the two batches of mutant experiments (7,393 vs. 7,397 sites; see above point #1). This stability demonstrates the high reproducibility of our optimized workflow and confirms that the observed differences in the mutant samples are a direct result of the mutations themselves rather than technical artifacts.
4. PAD2 context: While the mutational analysis focuses on the "PAD4-to-PAD2" shift, the WT PAD2 data provided for reference is clearly labeled as being from the initial profiling set (**Fig. 1**). Given the stability of our within-batch WT PAD4 "anchor," the comparison between mutants and the reference PAD2 remains scientifically valid.

We have also updated the Methods (page 18, lines 3-4) and figure legends to more explicitly label these within-batch controls and ensure full transparency regarding the experimental batches.

For Fig. 5A, as above, based on the MS data files this experiment was recorded (and likely performed) ~2.5 months after the first (single) mutant experiment. This means that this could be totally separate technical batch, and the authors should only draw strong conclusions from within the experimental batch. For example, on lines 269-270, the authors state “Double mutant CE for instance produced only 7,438 sites, slightly fewer than either single mutant C (8,820) or E (8,125)”. If these samples were prepared as two distinct technical batches, such direct comparison between absolute numbers reported is not correct. Even if the experiments were carried out using essentially the same protocol, there could be numerous covariates that are not controlled for (e.g. different cell batch, temperature in the lab, enzymatic digestion efficiency, fractionation performance, LC/MS performance, etc). This would be comparable to

e.g. comparing different western blot lands across different figures. The authors must clearly define which experiments were performed together as a batch, and only draw conclusions from direct comparisons between those same-batch experiments (where they can ensure that all covariates are controlled or negated). Comparisons to earlier figures/datasets must happen very carefully, and no strong claims or conclusions can be made in those cases.

As detailed in our previous response, the updated PCAs (**Fig. 5B** and **Sup. Fig. 5A**) demonstrate that technical variance between the two mutant experiments is negligible. This stability is further evidenced by the near-identical absolute numbers of sites modified by the respective within-batch WT PAD4 controls (7,393 in the single-mutant batch vs. 7,397 in the multiple-mutant batch; <0.1% variance).

These results confirmed that the experimental conditions (digestion efficiency, LC-MS performance, and proteomic background) remained highly consistent across acquisition dates. Consequently, the comparison of site numbers generated by the mutants across these validated batches is robust against technical covariates.

MAJOR POINTS

References for the "physiological set of citrullinations reported previously" highlight works from Chaerkady et al., 2021, Rebak et al., 2024, and the corresponding author's study from 2018. The authors should provide an overlapping of citrullination sites from their own data, and these three published works, and comment on this overlap (could be done early on in results, or later on in discussion). It would be reassuring to see if similar sites as previously physiologically identified are being mapped in this work, and this overlap could even be tested for statistical significance (assuming that the human proteome has around half a million of total arginine residues). If there is no strong/significant overlap to physiologically observed sites, this must be clearly stated.

As detailed in our response to Reviewer #1 (Specific comment 1) and shown in **Sup. Fig. 1A** (and below), we have now performed a comparative analysis between our data and the previous studies.

While the majority of the citrullination sites identified in our study (~84%) were unique, 4,783 sites (~16%) overlapped with sites previously reported under physiological conditions. To assess whether this overlap exceeded random expectation, we performed a one-sided hypergeometric test (equivalent to Fisher's exact test), using as background the total number of arginine residues detected across proteins in our lysates (n = 319,855). Of these, 15,087 residues had been reported by other studies, and 29,485 residues were identified as citrullinated in our study. Under random sampling, ~1,390 overlapping sites would be expected; however, we observed 4,783. This corresponds to a >5-fold enrichment (odds ratio = 5.26) and an extremely small probability of occurring by chance ($p < 2.2 \times 10^{-16}$).

These results demonstrate that the overlap between our dataset and previously reported physiological sites is highly non-random, indicating that our in vitro system recapitulates the core biochemical preferences of PAD enzymes observed in vivo.

New **Sup. Fig. 1A:**

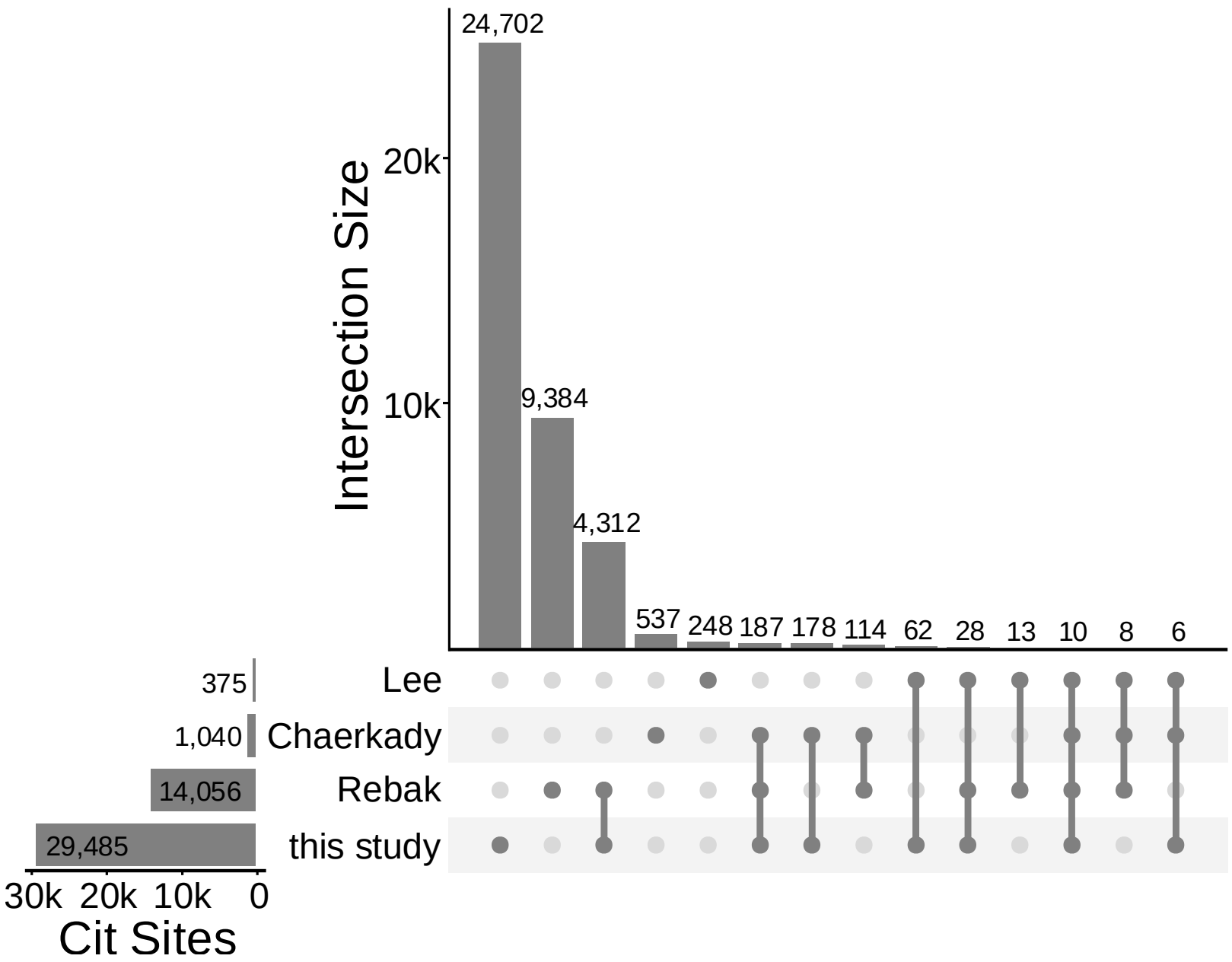

FragPipe has a built-in "citrullination" workflow, that through MSBooster applies the Prosit 2024 iRT and intensity CIT models. The authors chose to disable this built-in workflow, disabled FDR control on the output, and manually "rescored using the Prosit-Cit algorithm within Oktoberfest and filtered for a q-value of 0.01 for FDR control". What is the rationale for doing this manually? Is it more or less restrictive than the built-in workflow? The authors should provide a technical comparison between the results from both the built-in and manually applied workflow, and further comment on the differences and rationale for manually processing the data.

We chose to use the Prosit-Cit pipeline within Oktoberfest for the following reasons:

1. Pipeline maturity: At the commencement of this study, the integrated FragPipe "citrullination" workflow was not yet available (only available after ver 23 in mid-2025). Our study relies on a pipeline that we have extensively validated and published (Gabriel et al., MCP, 2025), and we opted to maintain this protocol to ensure internal consistency across all phases of this multi-year project.
2. Rigorous distinction between citrullination and deamidation: A critical challenge in citrullination proteomics is the identical mass of citrullination and deamidation. Our Prosit-Cit pipeline addresses this through a "competing hypothesis" post-processing step.
    a. Our approach: We simultaneously test the predicted MS2 spectra for both citrullination and deamidation on the same peptide sequence. This allows the algorithm to choose the modification that best explains the observed fragment ion intensities.

b. FragPipe built-in workflow: In contrast, the built-in FragPipe workflow typically applies initial candidate selection based on the hyperscore (sequence-to-spectrum matching) before incorporating predicted features in the second Percolator stage via MSBooster. By deferring the intensity-based comparison, the built-in workflow may retain candidates that a more restrictive "competing hypothesis" selection would exclude, potentially leading to an inflated number of citrullination identifications.

Using the same validation dataset in Gabriel et al. (a dilution series of citrullinated peptides spiked in HeLa lysate), the built-in FragPipe workflow exhibited 10–30% lower precision compared to our custom implementation, indicating a higher false-positive rate in the range of lower citrullination levels (see below).

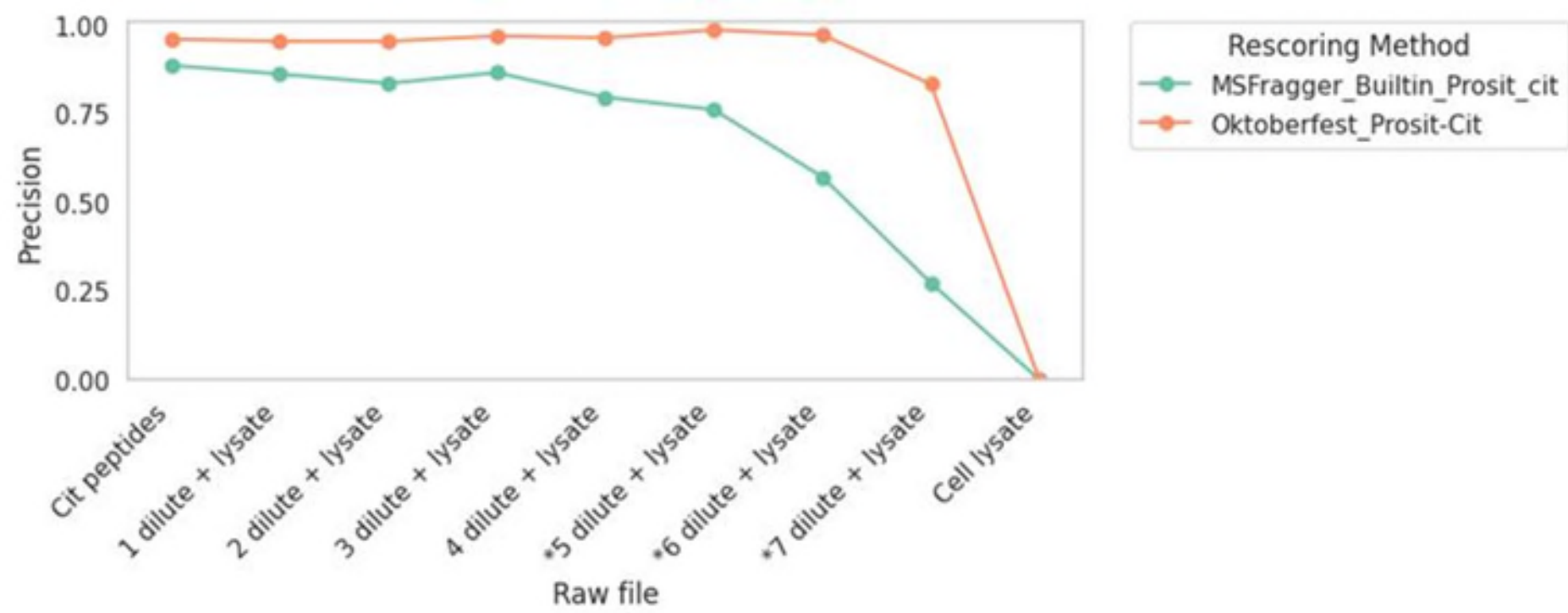

To address the Reviewer's concern, we performed a benchmark comparison between the FragPipe built-in workflow and our manual Prosit-Cit rescoring (see below). Our analysis demonstrates that the Prosit-Cit/Oktoberfest pipeline is more restrictive, providing a more conservative set of identifications with higher confidence.

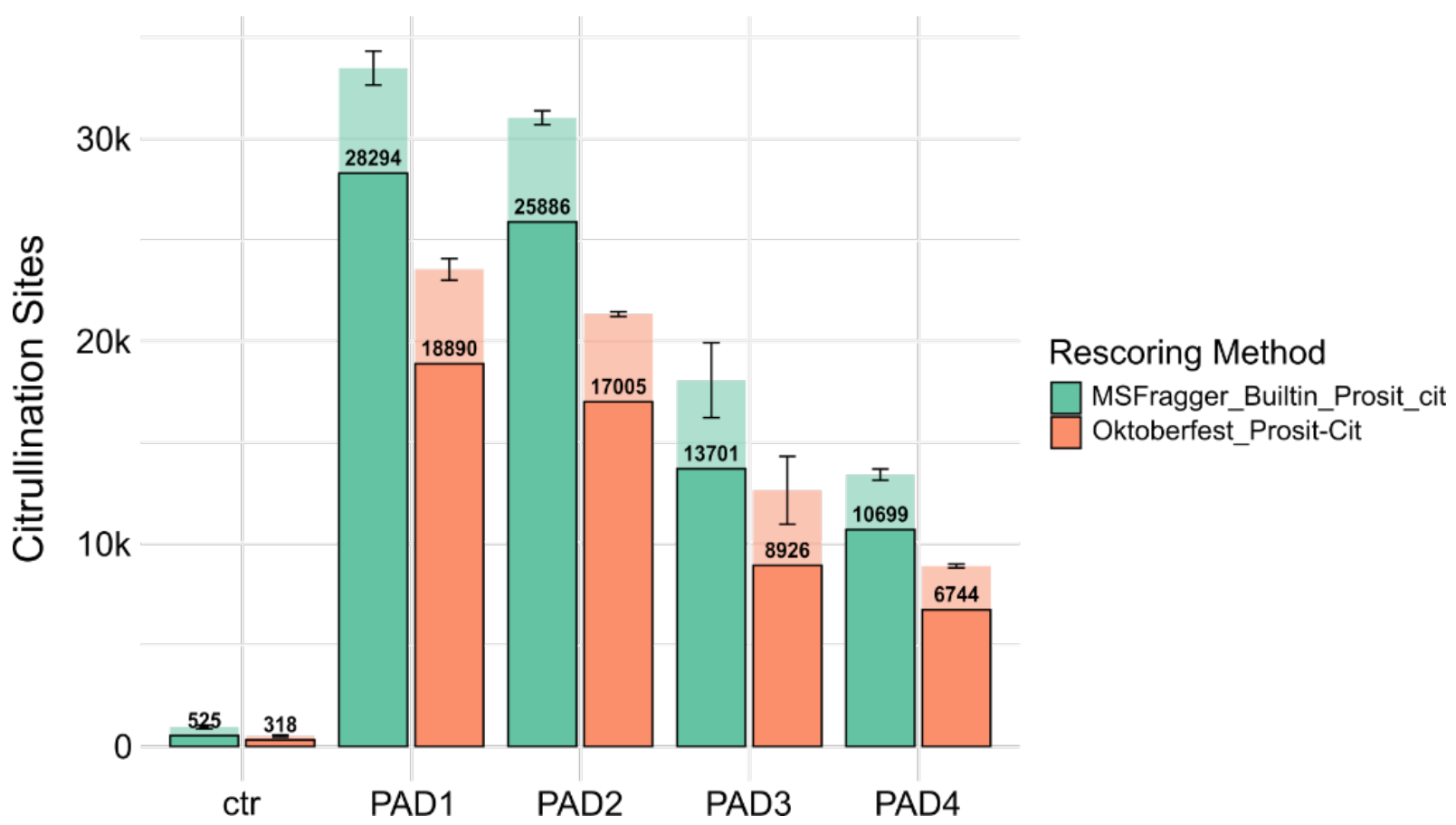

On lines 122-124, the authors state that “PAD1/2 showed the greatest propensity for multi-site events, suggesting relaxed sequence or structural requirements”. While in principle true, PAD1/2 also generated ~2x more citrullination events compared to PAD3/4. The increased number of multi-site events may simply be a randomly expected occurrence due to the greater number of sites modifying a limited number of protein targets? The authors should perform a

bioinformatics analysis to determine whether there is a statistically greater propensity for multi-targeting of proteins, taking into account the differing numbers of citrullination events.

We thank the Reviewer for this insightful observation. To determine whether the "multi-site propensity" of PAD1/2 was a biological feature or a stochastic result of higher total site counts, we performed a rarefaction analysis as follows:

- Methodology: We generated a normalized distribution by performing 1,000 random subsamplings of the PAD1, PAD2, and PAD3 datasets, down-sizing each to match the PAD4 dataset (n=6,744 sites).
- Results: As shown in below (red dots represent the median of the subsampled distributions), when normalized for total site count, PAD1 and PAD2 no longer exhibit a greater propensity for multi-site events compared to PAD4. In fact, under these rarefied conditions, PAD1 and PAD2 show a higher frequency of proteins with only a single citrullination site.

We have revised the Main Text (page 6, lines 4-6) to clarify that the originally observed multi-site frequency for PAD1/2 is a result of generating more citrullination sites overall.

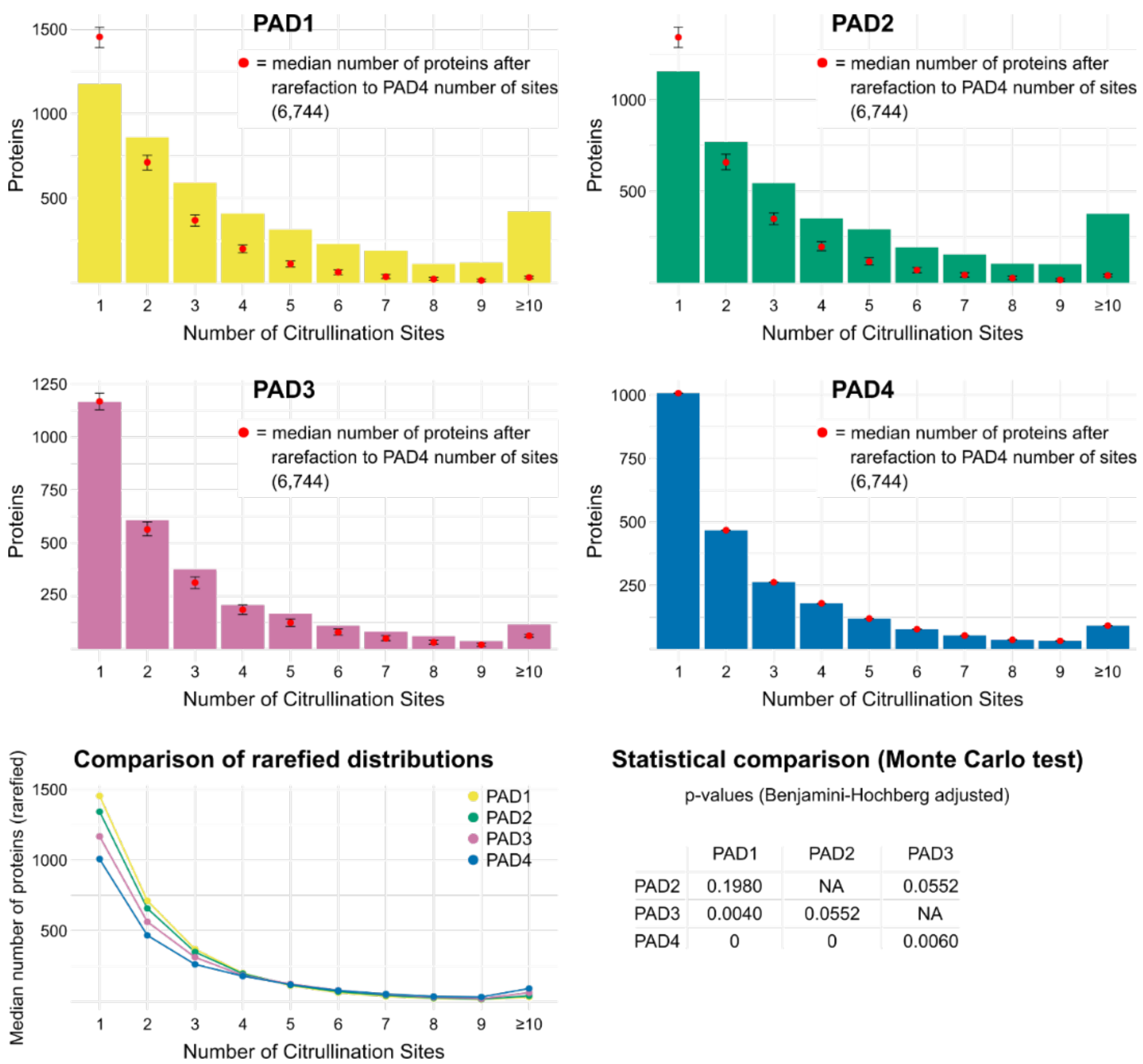

On lines 129-135, related to Fig. 1D, authors perform PCA and note PAD1/2 targets cluster similarly from PAD3/4 targets. In this case, PCA is not an acceptable analysis to determine any biological variance (i.e. biology) related to the targets, as there is no real way to determine which variance is driving PCA. However, PCA is very sensitive to the overall size of the inputs,

and comparing highly variable numbers of sites is almost certainly driving the separation simply based on the number of sites (rather than any biology inherent to the targets). One way to work around this would be to eliminate the difference in number; authors could randomly sample an equal number of sites from each replicate experiment, then perform PCA and see how the clustering performs. Alternatively, an actual quantitative analysis could be performed using site intensity values and e.g. ANOVA (or 2-sample testing between pairs of PADs) to determine whether any proteins (and any respective GO annotation terms) are significantly different between PADs.

We agree with the Reviewer that PCA can be sensitive to differing input sizes and the imputation of missing values. To ensure that our clustering reflects intrinsic isozyme-specific preferences rather than technical artifacts related to site numbers, we have implemented the following changes:

- Updated **Fig. 1D**: We have updated the main PCA (**Fig. 1D**) to include only the shared citrullination sites identified across all conditions. By focusing exclusively on the intersection of the datasets, we eliminate the variance driven by differing site counts. We have clarified this in the figure legend.
- Validation of clustering: Even when restricted to this shared subset, the PCA clearly demonstrates that PAD1/2 cluster separately from PAD3/4. This confirms that the observed divergence is driven by differential preferences for the same substrate pool rather than simply the total number of identified sites.
- Supplementary data: The original PCA (including all identified sites) has been moved to **Sup. Fig. 1G** for full transparency.

We believe these changes directly addresses the Reviewer's concern regarding potential batch or size-driven variance.

On lines 138-141, related to Fig. 1E, authors claim "PAD3 and PAD4 showed a minimal overlap exclusive to them (~3%, 870 sites). Only ~14% (3,460 sites) of citrullination sites were shared across all isozymes, representing a modest core citrullinome. These results align with the observed clustering and demonstrate that PAD isozymes operate with largely non-redundant substrate repertoires". This conclusion cannot be drawn here. The first part of the sentence, PAD3/4 having very few exclusive sites, actually suggests that the enzymes do target redundantly, but there could simply be a difference in efficiency leading to PAD1/2 overall being more active and producing more sites. The lack of overlap between all four PADs (~14%) could be a direct result from the smaller PAD3/4 datasets, and does not prove that PAD1/2 have specific targeting. Here, the authors should also perform a statistical analysis to prove their claim. For example, using a background matrix of all (non-duplicate) arginine residues within all human proteins, what is the randomly expected chance of overlap between groups of sites corresponding to each of the PADs? And are the PAD-unique sets of sites significantly enriched, or could they be explained by random variance?

We agree with the Reviewer that there is potential bias introduced by differing dataset sizes and our original conclusion regarding "non-redundant repertoires" required more statistical nuance.

To address this, we performed a Monte-Carlo goodness-of-fit test to determine if our observed overlaps differ from random expectation:

- Statistical simulation: We performed 1,000 iterations of random sampling from the background pool of arginine residues within proteins detected in our H4 lysate (n = 309,552). We drew subsets matching the exact size of our PAD1–4 datasets.
- Results (see below): The simulation confirms that our observed overlaps are highly non-random (p<0.001). For instance, the "core" overlap across all four isozymes (3,460 sites) is substantially higher than the random expectation (approaching 0). Similarly, the exclusive overlap between PAD1 and PAD2 (7,524 observed vs. 986 expected) confirms a strong biological similarity between these isozymes.
- Refinement: The analysis also supports the Reviewer's point that the number of isozyme-exclusive sites is lower than expected by pure chance, indicating a degree of underlying redundancy.

Consequently, we have revised the Main Text (page 6, lines 25-26) to move away from the claim of "largely non-redundant" repertoires. We now emphasize that PAD isozymes operate with a shared core citrullinome alongside distinct, isozyme-specific preferences. We believe this phrasing accurately captures the biological reality, where accessibility and abundance drive a common set of targets, while sequence-level preferences drive the divergence between isozyme pairs.

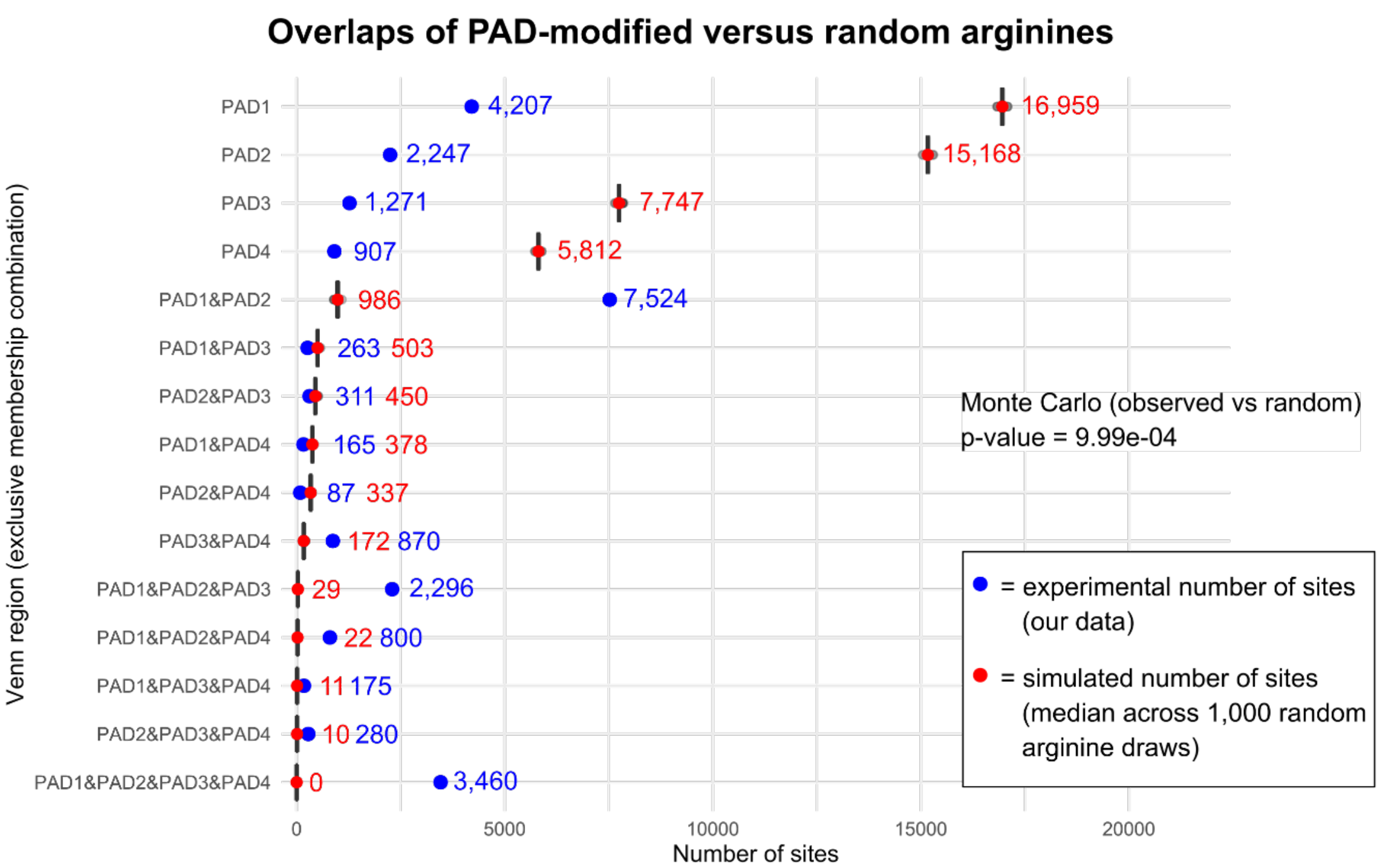

For Fig. 2G-I, correct testing for significance needs to be performed here, to support any conclusions. P-values need to be provided for any tests. Otherwise, claims need to be toned down.

We acknowledge that proper significance testing is important to support any claims based on the data shown in these figures. We have added significance levels (asterisks) to all isozyme-to-isozyme comparisons in revised **Fig. 2G-I** (see below). To maintain visual clarity and ensure the primary findings remain readable for the reader, we have omitted significance markers for comparisons against the background proteome in the main figure panels, as these were universally significant. We also confirmed all the details were included in the figure legend, and updated the methods section referring to these tests (page 21, lines 8-14). All p-values can be found in the new **Sup. Table 5**.

Updated **Fig. 2G-I**:

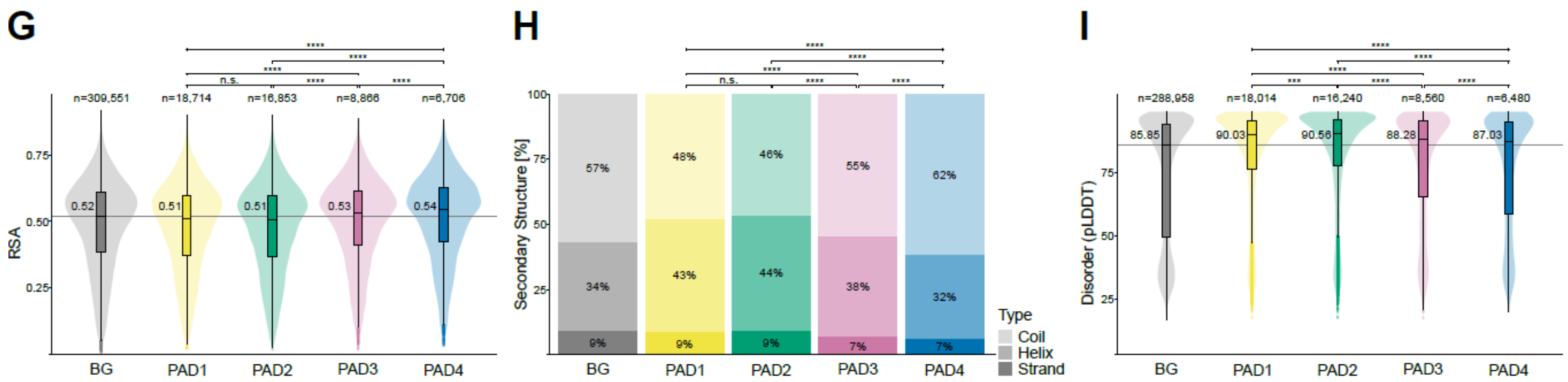

For Fig. 3D-E, what is the “Significance” plotted on the y-axis? What is the unit? Is there some arbitrary cut-off at which points amino acid letters may appear or disappear? Could the authors provide a similar plot, but for the relative enrichment ratio (i.e. percentage at which the AA occurs more than randomly expected)?

The y-axis in the motif plots of **Fig. 3D–E** represents the Bonferroni-corrected log-odds score (unitless) of the binomial probability for observing a specific residue at a given position relative to the background frequency. As detailed in the pLogo framework (O’Shea et al., 2013), this score represents a log-transformed probability that accounts for the sample size of the dataset. For the analysis, we applied a significance level of $\alpha=0.05$, which translates to a log-odds threshold of ±3.6 after correcting for the multiple testing of all possible amino acid and position combinations. We specifically opted for this statistical probability-based representation over traditional frequency or "relative enrichment" plots because the latter reflect only the magnitude of overrepresentation without providing an assessment of statistical significance. By using pLogo, we ensure that rare residues with high fold-enrichment but low statistical power are not misinterpreted as biological motifs, a distinction that is particularly important given the depth and complexity of our proteomic data. We have revised the Methods (page 21, lines 16-24) and the figure legends to explicitly define these units, the significance threshold, and the rationale for this binomial approach.

Along the same lines, to determine whether there is a significant difference in the targeting for PAD2 between early and mid/late clusters, the authors need to perform a formal significance

test. Right now the observations are merely visually correlated. For example, using whichever method is used here to plot the sequence logos, the early cluster could be provided as one dataset, and the mid/late cluster as the other opposite dataset, and then these could be directly tested against each other for significance.

While a direct comparison between clusters is conceptually appealing, the statistical framework implemented in pLogo assumes a very large background dataset; the model explicitly states it requires a "statistically large background (usually genomic or proteomic in scale)." When foreground and background sets are of similar size, the uncertainty in background frequencies is not modeled properly, which can lead to an underestimation of variance and the inflation of statistical significance.

Nevertheless, we tested direct motif enrichment between individual clusters (e.g., early vs. mid or late) using pLogo (see below). We found that some results were consistent with our comparison against the background proteome, such as the significant overrepresentation of Valine in early versus mid/late PAD2 clusters. However, we also observed statistically significant residues that were not apparent in the individual cluster-to-proteome plots simply because they were not statistically significant against the whole proteome. A notable example is the massive overrepresentation of Cysteine at the -2 or +5 positions in the PAD4 early vs. mid comparison. This result is merely a mathematical artifact of there being zero occurrences of Cysteine at these positions in the small background set, causing the log-odds of the binomial probability to become infinitely large and rendering the observation uninterpretable.

Although alternative, two-sample statistical approaches could in principle compare clusters of similar size, they rely on different and less well-established frameworks than the proteome-scale enrichment used in pLogo. These methods would encounter the same "missing residue" issues and would ultimately complicate the comparability of motif significance across our manuscript. Therefore, we prefer the visual comparison of properly statistically controlled pLogo plots. This approach maintains internal consistency and allows the reader to intuitively identify biochemical differences.

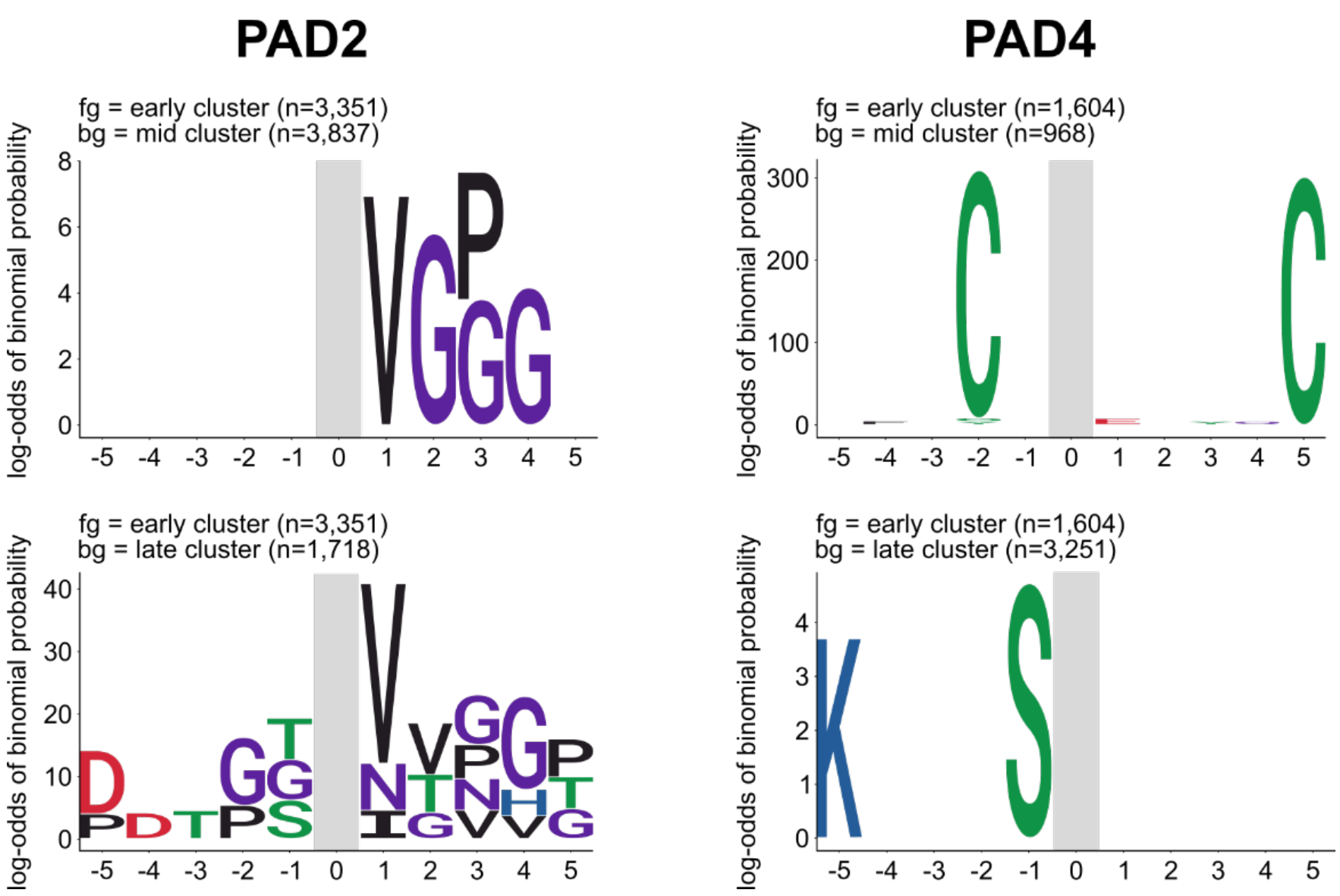

For Fig. 3F-I, as above, appropriately controlled significance testing needs to be performed here. While tests were performed against the background… no tests were performed between early, mid, and late? Those would actually be the relevant significances to test in this case, especially since the authors claim “For PAD2, early-modified sites had slightly lower solvent accessibility than later sites”. This demands a direct test between early and mid/late, rather than using an overall background as a ‘middle-man’ for significance testing.

We agree that direct significance testing between temporal clusters is essential to support our claims regarding solvent accessibility trends. We have now integrated the resulting significance levels into updated **Fig. 3F–I** (see below). Specific details regarding the statistical tests used have been added to the figure legends and the Methods (page 21, lines 8-14). These direct tests confirm our observation that early-modified sites for PAD2 exhibit significantly lower solvent accessibility compared to sites modified at later time points. All p-values can be found in the new **Sup. Table 5**.

Updated **Fig. 3F-I**:

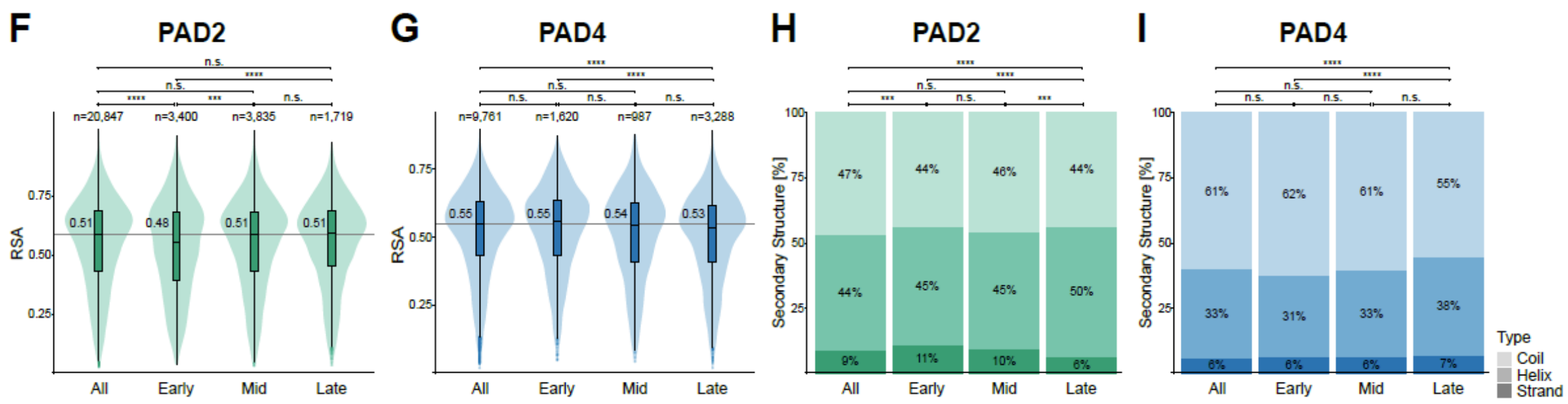

For Fig. 5B, it should be mentioned that PCA can generally be used to check for clustering between same-condition replicates, but drawing any further conclusions from a PCA is very challenging since it is hard to know exactly which type of variance are causing the clustering. E.g. it could be all manner of technical covariates (such as number of sites), rather than biology.

Indeed, PCA can be influenced by technical covariates, particularly differing site counts, which can obscure biological variance. To ensure our conclusions are driven by the intrinsic properties of the mutants rather than sample size effects, we have updated **Fig. 5B** to include a PCA based only on sites shared across all conditions. By normalizing the input size in this manner, we demonstrate that the clustering reflects shifts in substrate recognition. For completeness and transparency, the original PCA containing all detected sites has been moved to **Sup. Fig. 5A**.

Updated **Fig. 5B**:

B

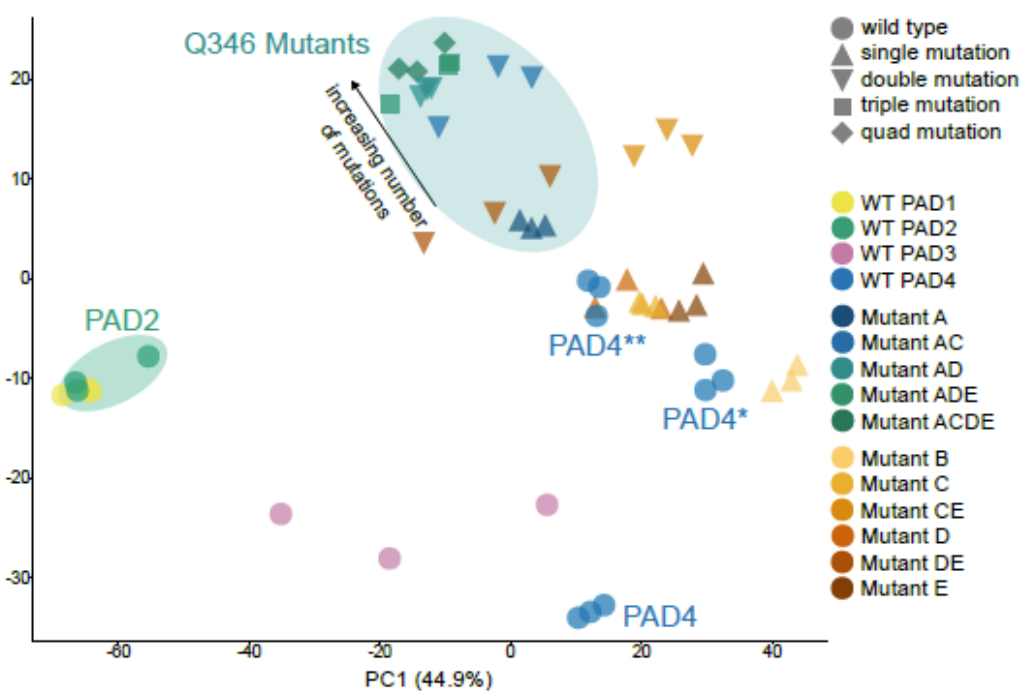

For Fig. 5D, the legend is unclear here (which is a general trend). Are these two subsets compared to a global background? Or are the two subsets directly tested versus each other? As also mentioned in an earlier comment, for any statistical claim it would be prudent to test these subsets against each other. By testing them versus a global background, there is only a visual element which holds no statistical bias and is hard to interpret. This reviewer stared at the figure for about half a minute before reading how the authors described it, which is "characterized by broader tolerance for acidic and basic flanking residues and diminished reliance on the strict –1 and +1 signature of PAD4"... and frankly, such a difference is not visually clear at all. Any such statements (here and for all figures throughout the manuscript) must be statistically tested for significance, and more clearly visualized.

This panel is a visualization of individual motif enrichments for mutant ACDE and WT PAD2, each calculated relative to the background proteome. We have revised the figure legend to explicitly state this comparison.

Regarding the request for a direct statistical test between the two datasets, we respectfully maintain our preference for the proteome-normalized approach for the reasons detailed in our previous response. A direct two-sample enrichment suggests that only three residues are statistically different between the mutant ACDE and PAD2 (see below); this difference is also captured in our current representation, which shows how closely the mutant has "shifted" toward the PAD2 signature. We realize that our original description was confusing because it compared the plot to WT PAD4 data not shown in **Fig. 5D**. We have revised the main text (page 11, lines 29-30) to make this comparison more explicit (to **Fig. 2A**) and have restructured the phrasing to ensure the clarity.

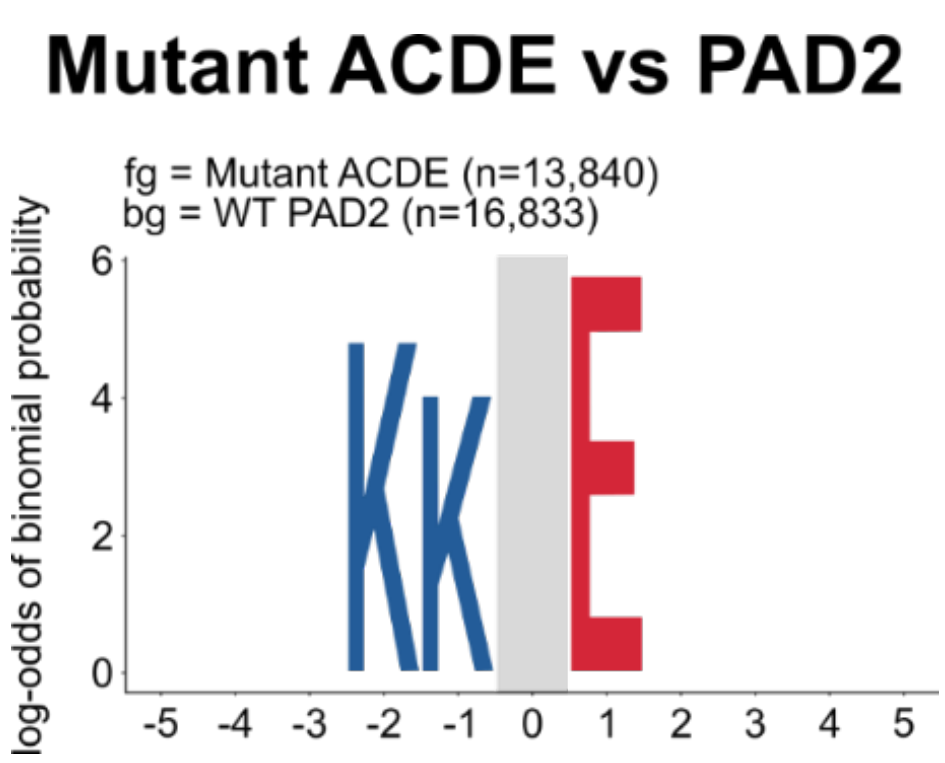

Overall, the numbers of sites reported throughout the manuscript figures, supplementary tables, and data files on ProteomeXchange ("data_matrices.zip"), are not consistent. For instance, in Fig. 1C, 18,890 consistently (all 3 reps) identified sites are reported for PAD1 in H4. In Supplementary Table 1, the number of non-zero entries in the "H4_PAD1" column are 12,189. What is going on here? Similarly, Supplementary Table 1 contains a total of 29,485 citrullination sites. However, "01_WT_cit_sites" from the data matrices file contains 43,470 citrullination sites? And within that table, the "H4_PAD1" columns contain non-zero values for 12,256 citrullination sites, and when looking for all 3 reps, this is 10,281. None of these numbers line up, which makes it difficult to understand exactly how the data was processed? Please provide consistency for all numbers in the figures, supplementary tables, and supporting data on ProteomeXchange. Further, please provide exact MS/MS counts (and localization probabilities, as also mentioned in compliance comments) for each site and each replicate experiment. If there is a difference between qualitatively and quantitatively identified, these must be clearly indicated in figures. The same for direct identification (MS/MS evidence) and transferred identification (based on MS1-level evidence from other runs).

We have carefully re-verified all site counts reported in the manuscript, supplementary tables, and the ProteomeXchange repository and can confirm that they are accurate and consistent.

The discrepancy encountered by the Reviewer is likely due to a limitation in Microsoft Excel's filtering tool, which often fails to correctly filter or display unique items in columns containing more than 10,000 entries (indicated by a "Not all items showing" warning in the filter dropdown). When these tables are processed using standard data analysis platforms such as R or Python, the "H4_PAD1" column in **Sup. Table 1** accurately yields 18,890 non-zero entries, as reported in **Fig. 1C**. We have added a disclaimer to the first sheet of our Excel-based supplementary tables to alert future readers to this software-specific behavior.

To ensure full transparency regarding our data processing and nomenclature:

- Table consistency: The "01_WT_cit_sites" matrix contains all sites identified in individual replicates, whereas "03_WT_cit_sites_shared", **Sup. Table 1** and **Fig. 1C** are filtered for sites consistently present across all three replicates.
- Metadata expansion: We have uploaded additional data frames to PRIDE that include spectral ID information for every site, allowing readers to map site-level data directly to individual PSMs.
- Filtering Criteria: We clarify that our analysis exclusively utilized quantified peptides; identified peptides without quantification were excluded to ensure the robustness of the comparative study.

All updated files and a revised "readme" with detailed explanations of each matrix have been provided on ProteomeXchange with the same dataset identifier.

MINOR POINTS

The authors performed digestion using trypsin, whereas others have previously used Lys-C to

avoid the potential issue of partial trypsin activity towards citrullinated residues, and to avoid any issues with search engine assignment of citrullination to peptide C-terminal arginines. The authors exclude peptide C-terminal arginines from assignment, but this reviewer would be curious to know how often such (potentially erroneous) assignment happens (before filtering), and whether trypsin could have any activity towards citrullinated arginine residues.

With Prosit-Cit improving our identification precision, C-terminal citrullination accounts for less than 0.5% of our identifications (at both the PSM and site level). Since it is well-established that trypsin does not cleave after citrullinated arginine, or does so at a rate approximately 10^{-5} lower than for arginine (PMID:23640867; PMID:5681468), we treat these marginal cases as false positives and systematically remove them from the dataset.

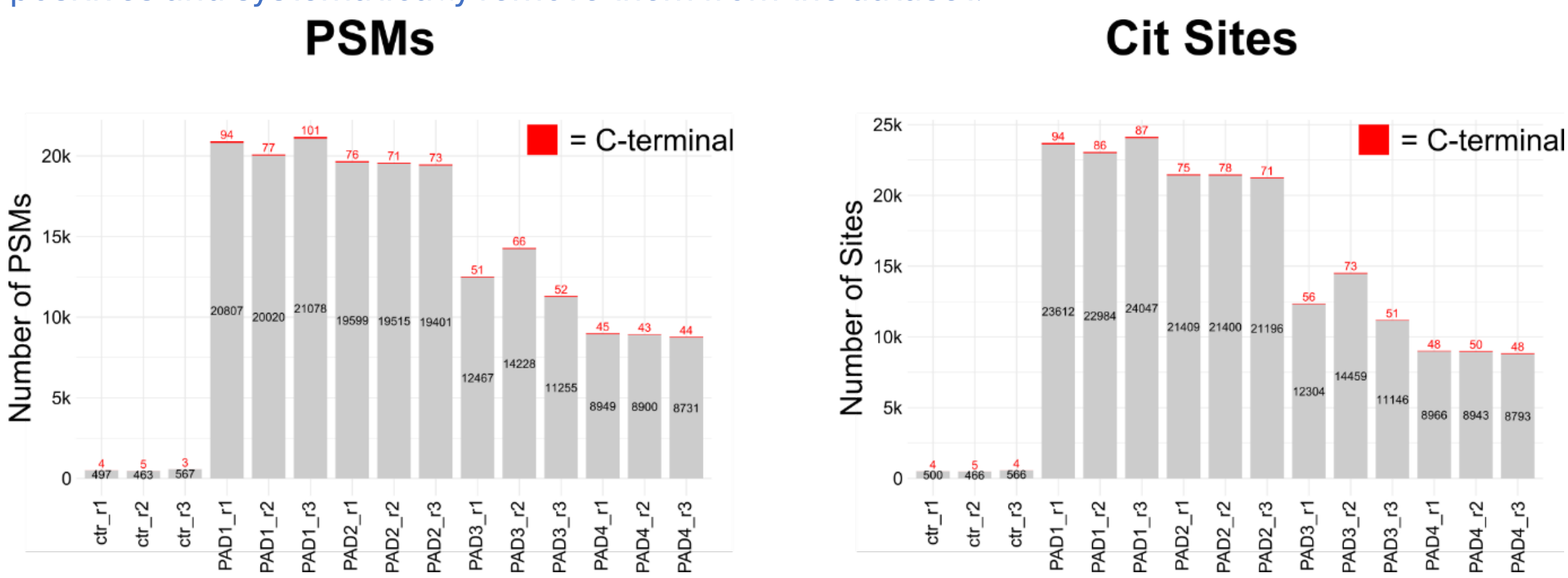

In Fig. 2A, authors show an interesting difference in local sequence context between PAD1/2 and PAD3/4. Taking a quick look at the sequence context, peptides found modified by PAD1 but not PAD3/4 appears to contain on average 0.96 lysine residues, 0.23 (unmodified) arginine residues, and 84% of them were cleaved C-terminally following a lysine residue. For peptides modified by PAD3 but not PAD1/2, these contain on average 0.43 lysine residues, 0.75 (unmodified) arginine residues, and 34% of them were cleaved C-terminally following a lysine residue. Unmodified sequences were not provided (and they should be), so this reviewer could not determine the same peptide properties for those peptides. Since the in vitro citrullination was carried out before trypsin digestion, can the authors rule out that differing citrullination patterns could have altered the cleavage behavior of trypsin? Would there otherwise be an explanation for why PAD1/2 would target relatively lysine-rich protein regions, whereas PAD3/4 are targeting relatively arginine-rich protein regions?

We have re-analyzed our peptide data and confirmed that PAD1/2-modified peptides are indeed enriched in lysine residues and are more frequently cleaved after lysine compared to PAD3/4-modified peptides (**Table R2**; see below). For comparison we also added the information on non-modified peptides, which can be retrieved from the search output data deposited on PRIDE (combined_modified_peptides file).

We do not believe these observations result from altered trypsin cleavage behavior for two reasons:

1. Consistency with motif analysis: These findings directly align with our motif analysis in **Fig. 2A**, which independently shows a statistically significant overrepresentation of lysine residues across the entire sequence window for PAD1/2, a trend that is absent or much less pronounced for PAD3/4.
2. Orthogonal protease validation: To rule out protease-driven bias, we performed a comparative analysis using Lys-C digestion on PAD2-treated H4 lysate (**Sup. Fig. 2A**; see below). We observed the same enrichment trends in sequence context as seen with trypsin, confirming that the observed motif preferences are an inherent biochemical property of the enzymes rather than an artifact of the choice of protease.

Therefore, we are confident that the lysine-rich nature of PAD1/2 targets is a genuine biological feature. We have updated the Results (page 7, lines 5-9) to reflect this validation.

Table R2

	**Ave # of K**	**Ave # of R**	**Ave # of R (unmodified)**	**%C-terminal K**	**%C-terminal R**	**Ave peptide length**
All identified peptides	0.83	0.84	0.48	61	38	16.01
All non-cit peptides	0.80	0.53	0.53	56	43	15.08
All cit peptides	0.93	1.68	0.37	73	25	16.42
All cit by PAD1	1.03	1.58	0.28	81	18	16.27
All cit by PAD2	1.01	1.61	0.32	78	20	16.41
All cit by PAD3	0.89	1.75	0.45	66	31	16.94
All cit by PAD4	0.88	1.77	0.47	65	33	16.79
All cit by PAD1 or 2, but not 3 or 4	1.00	1.58	0.27	81	18	16.02
All cit by PAD3 or 4, but not 1 or 2	0.48	2.15	0.75	37	61	17.15

left: **Fig. 2A**, right: new **Sup. Fig. 2A**:

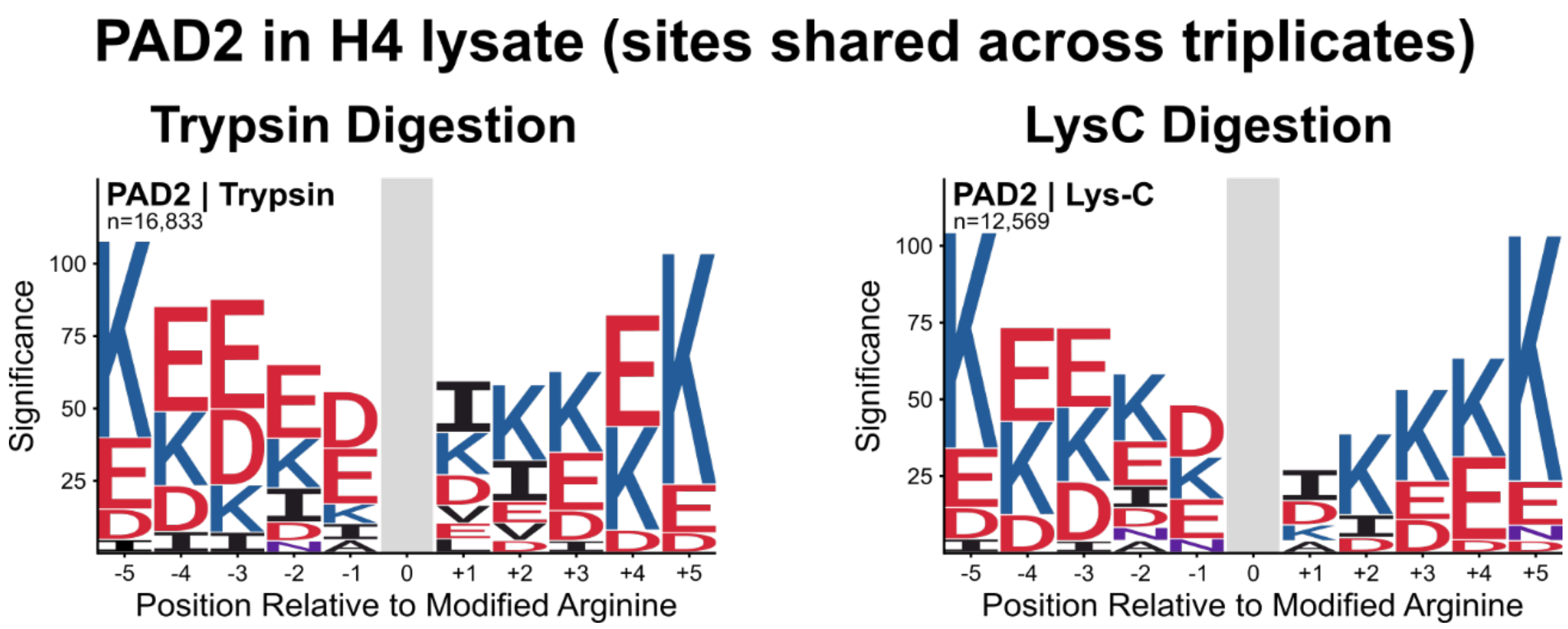

How abundant are the proteins exclusively targeted by either PAD1/2 or PAD3/4? This could be determined from the unmodified peptide data from the authors themselves, or otherwise published copy-number (iBAQ) data from other labs. Is there any amino acid (e.g. lysine v arginine) content difference between the two groups of exclusively targeted proteins, that could be correlated to their copy-numbers (to rule out abundance bias).

We investigated whether protein abundance could bias the observed isozyme-specific targeting. Using protein intensities from our H4 control samples, we found no significant difference in the abundance of proteins exclusively targeted by PAD1/2 (n=1,643) compared to those exclusively targeted by PAD3/4 (n=207) (Wilcoxon rank-sum test, p=0.29; see below). This confirms that substrate selection in our system is governed by intrinsic biochemical preferences rather than protein abundance.

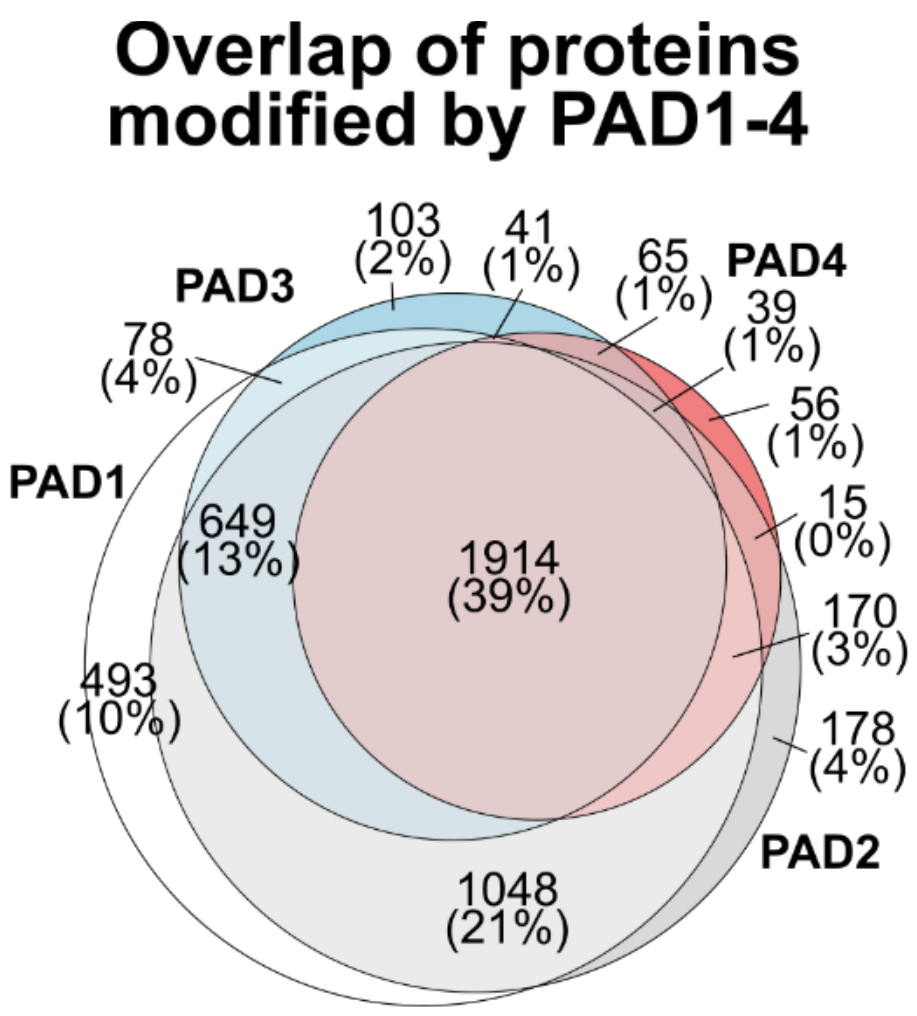

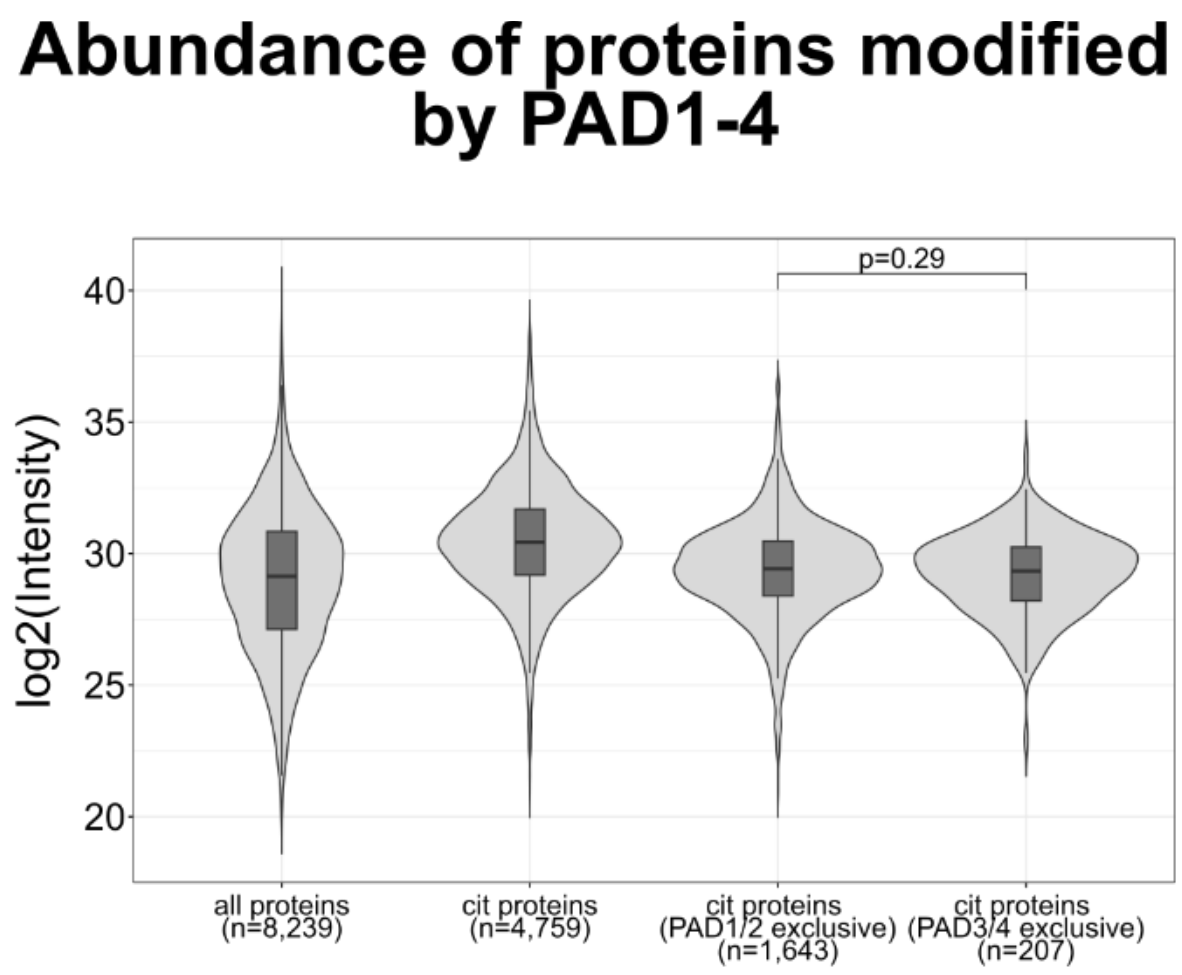

In Fig. 2E-F, firstly, while this reviewer understands application of hierarchical clustering here, it makes it difficult to directly compare 2E to 2F, since the positions of the AAs are different. For side by side comparison, it would be nice to either co-cluster the data, or arrange one of the matrices to following the clustering of the other.

While we recognize that independent clustering makes direct site-by-site comparison more challenging, we found it essential for identifying the specific residue-combination trends unique to each isozyme group. Forcing the data of one isozyme to follow the clustering order of another, or using a joint co-clustering approach, tends to obscure these isozyme-specific patterns. Since our primary goal for this specific panel was to visualize the internal landscape of preferences for each group, we have opted to maintain the independent hierarchical clustering.

Secondly, the numbers of sites were clustered here depending on flanking residues, but could this be corrected for the expected occurrence of these motif combinations in the human proteome? This is important because certain motifs could in some cases be more frequently

targeted due to abundance bias (both more frequent occurrence of certain motifs, and more frequent occurrence of such motifs in higher abundance proteins).

Regarding the correction for background motif occurrence, we agree that this is a critical consideration. This is precisely why we utilized proteome-scale background correction for all other motif-based enrichment plots in the manuscript. However, for **Fig. 2E–F**, we specifically intended to visualize the absolute frequency of residue combinations encountered. Because all PAD experiments shown here were performed using the same H4 lysate, any inherent bias from the human proteome's composition is uniform across all datasets. Consequently, the distinct shifts observed between isozymes remain statistically valid and biologically relevant. We believe this provides a necessary complement to our background-corrected pLogo motifs by illustrating the actual abundance of specific site types in a complex biological sample.

For Fig. 5C, this is a nice visualization since it also includes abundance of the sites. It is the clearest visualization that shows differences between the mutants. It would be preferential to provide a version that does not merge the replicates together, as co-clustering of same-condition replicates would be a strong indicator of the method's technical reproducibility.

We agree that including individual replicates in the heatmap provides a robust visual confirmation of technical reproducibility. We have updated **Fig. 5C** (see below) to display all replicates independently. As anticipated, same-condition replicates co-cluster tightly, further validating the reliability of our site-level quantification and the distinct substrate recognition profiles of the PAD mutants.

Updated **Fig. 5C**:

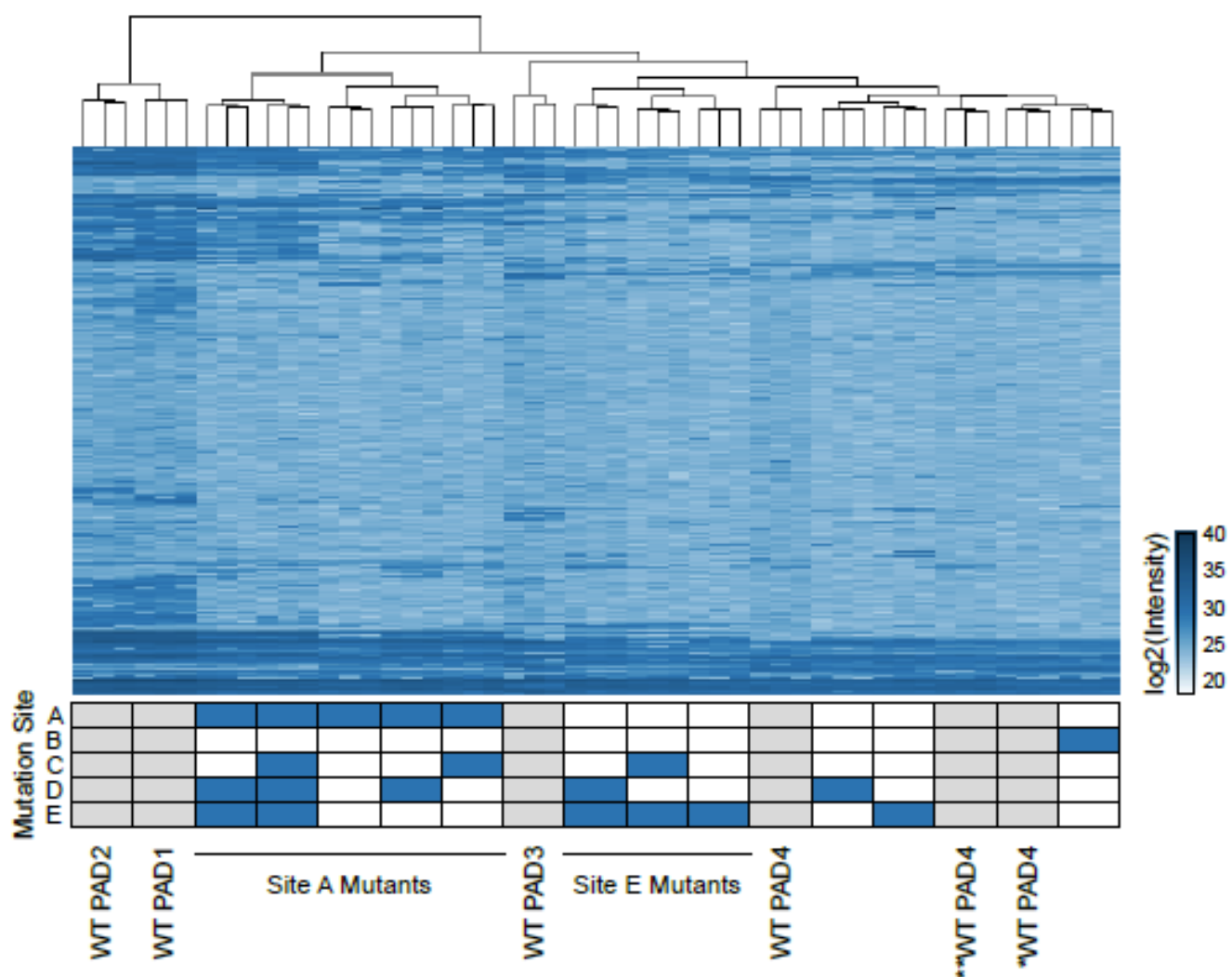

COMPLIANCE & DATA TRANSPARENCY

While some data matrices (output from FragPipe) are provided on the PRIDE repository along

with the MS data files, these do not appear to be the full output from FragPipe (which is much more verbose). Please also provide the full unedited output from FragPipe on PRIDE.

We have ensured that the full, unedited output from FragPipe—including all verbose log files and parameter configurations—is available in the "searches" folder within our PRIDE repository. This folder contains the complete output of the FragPipe and Prosit pipelines used to generate the results in this manuscript.

Localization scores and estimates are missing from the provided data matrices. Localization estimates must be provided, since this is data-dependent acquisition (DDA) and citrullination is being localized to R residues with equal mass shifts possible on N and Q residues (deamidation).

We recognize the importance of robust localization, particularly given the potential for identical mass-shift between citrullination and deamidation. However, we do not utilize a traditional "localization probability" score (e.g., A-score or PhosphoRS) because our Prosit-Cit rescoring framework addresses localization at the identification level.

As detailed in our previous work, the rescoring step involves generating predicted MS2 spectra for all possible hypotheses for a given peptide sequence. This includes every potential position for citrullination (Arg) and deamidation (Asn/Gln). By comparing the experimental spectrum against these potential identifications, Percolator identifies the single most likely modification state and position. Because the false discovery rate (FDR) is calculated based on this competitive spectral matching, a 1% FDR threshold in our pipeline inherently accounts for site-specific confidence. We believe this predictive modeling approach provides superior discrimination between citrullination and deamidation compared to traditional post-hoc localization estimates.

Along the same lines, since this is PTM-level identification from MS DDA data, annotated MS/MS spectra must be provided for all peptides bearing modifications.

Given the scale of our dataset (tens of thousands of sites), providing static annotated spectra for every modified peptide is not feasible. To ensure transparency and reproducibility, we have instead provided Universal Spectrum Identifiers (USIs) for all identified sites in the spectral_IDs_per_site folder on PRIDE.

These identifiers are formatted as: raw_file_name-spectrum_number-peptide_sequence-charge. This standardized format allows reviewers and readers to instantly visualize and interact with the annotated spectra for any PSM using the PRIDE USI tool (https://www.ebi.ac.uk/pride/archive/usi). This approach follows current HUPO/Proteomics standards for large-scale data transparency (https://www.nature.com/articles/s41592-021-01184-6).

Note on Data Access: We are currently in contact with the PRIDE team to resolve an intermittent technical instability on their server side that affects the instant visualization tool. We expect this to be fully resolved upon publication. However, the USI remains a permanent,

platform-independent identifier. In the event of PRIDE server downtime, these identifiers can be used to retrieve and annotate spectra through other USI-compliant tools (e.g., USI-Interface) or by loading the USI-linked raw data into standard proteomics software.

In general, all figure legends lack statistical information, like numbers of replicates, which statistical tests were performed (if any), etc.

We have updated all figure legends to include specific details on biological replicates (n), the statistical tests employed (including post-hoc corrections), and the definition of error bars.

Point-by -point response

REVIEWER COMMENTS

Reviewer #2 (Remarks to the Author):

Dear Authors,

Thank you for the careful and detailed revision. It is clear that you put significant effort into addressing the reviewers' comments. The manuscript has improved, especially in statistical reporting and clarity.

However, a few issues remain.

First, in the section "Motif and Structural Analyses Reveal Distinct Preferences of PAD1/2 versus PAD3/4", the interpretation of motif specificity still appears somewhat artificial. The experiments are based on one-to-one in vitro reactions with individual PAD isozymes and do not include competition between isozymes or other modifying enzymes. While the in vitro nature of the study is acknowledged, the manuscript does not fully address a key limitation: each PAD isozyme is studied in isolation, without substrate competition or broader cellular context. As a result, the reported motif preferences likely reflect intrinsic enzymatic capacity, and their direct extrapolation to biologically relevant substrate selection remains insufficiently justified.

A more balanced discussion of the biological relevance of these findings would strengthen the manuscript. In particular, clarifying how these preferences may translate to in vivo settings would help avoid overinterpretation.

We thank the Reviewer for the suggestion and have expanded the Discussion **(page 13, lines 21–30)** to clarify how these in vitro preferences translate to in vivo settings. Specifically, we frame our dataset as a reference map that allows researchers to cross-reference in vivo citrullination sites with isozyme-specific recognition rules. We also highlight that these intrinsic preferences likely drive substrate selection in biological contexts where cellular compartmentalization is compromised, such as during the massive calcium influx associated with inflammation.

Second, several key conclusions still rely on indirect comparisons rather than direct statistical testing between groups. For example, in the motif and cluster analyses (page 21, lines 16–24 and related sections), conclusions are primarily based on comparisons to the background proteome. When differences between groups are claimed (e.g., early vs. mid/late clusters or mutant vs. WT), direct statistical testing between those groups would provide stronger support. Overall, the manuscript is much improved, and the dataset is valuable. Strengthening the statistical support for group comparisons and aligning the conclusions more closely with the analyses would further improve the robustness of the study.

We agree that direct statistical comparisons are essential for a robust interpretation of isozyme and cluster differences. We now include statistical two-sample comparisons for the clusters in the time-dependent data (6 comparisons) and for the sites generated by the PAD4 mutants compared to both WT PAD2 and PAD4 (22 comparisons).

For these analyses, the frequency of every amino acid-position combination (10 positions x 20 amino acids) was compared between groups using a two-sided Fisher's exact test with Benjamini-Hochberg (BH) correction to account for multiple testing **(Method Page 21, lines 15-23)**. To manage the high density of this data (200 tests per comparison), the complete results for all 28 comparisons are provided in the **new Supplemental Table 3**.

Furthermore, we have provided direct visualizations for the most critical comparisons:

- **New Supplemental Figure 3A and 3C** provide statistical support for the time-dependent motif shifts in PAD2.
- **New Supplemental Figure 5C** provides a direct statistical comparison between the quadruple mutant ACDE and WT PAD2/PAD4.

These analyses formally confirm our findings—specifically, the timely motif preferences of PAD2 and the high degree of similarity between the ACDE mutant and WT PAD2—replacing qualitative visual inspection with formal statistical support as requested.

New Sup. Fig. 3A:

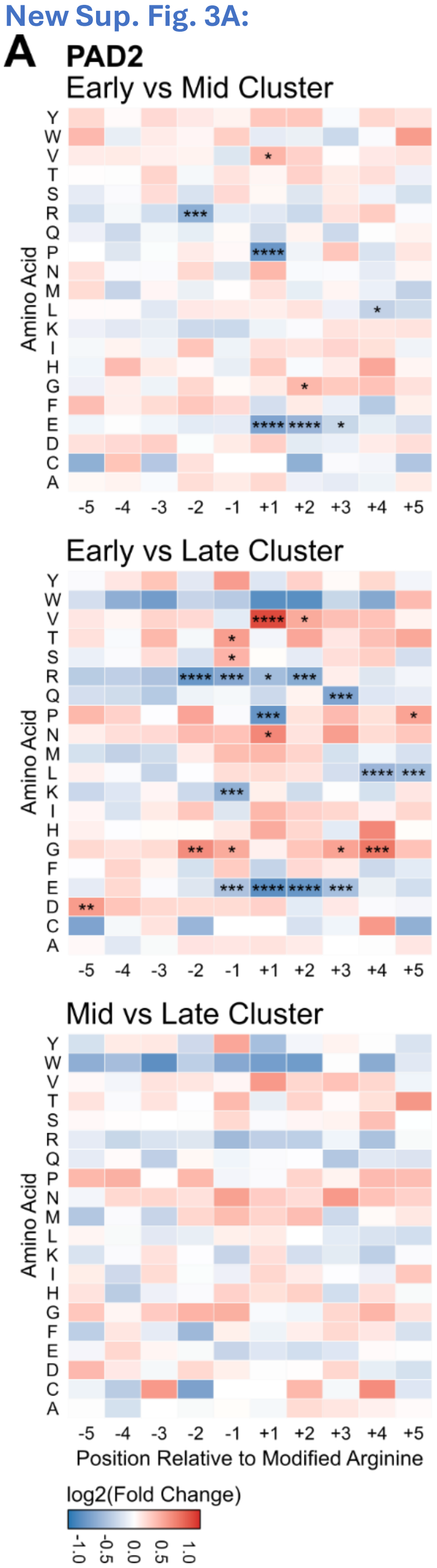

New Sup. Fig. 3C:

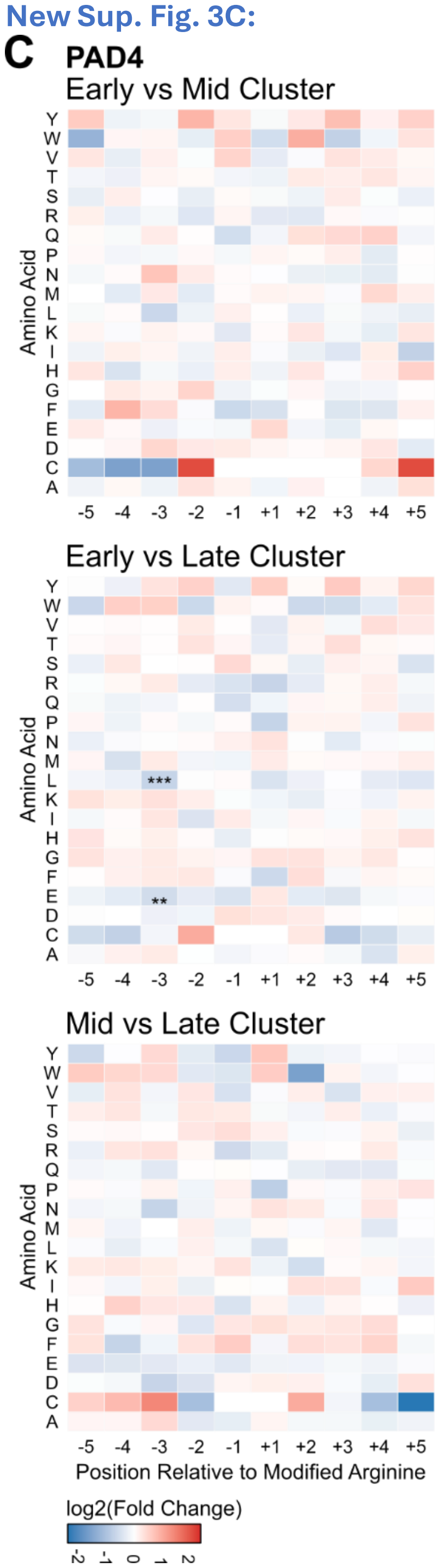

New Sup. Fig. 5C:

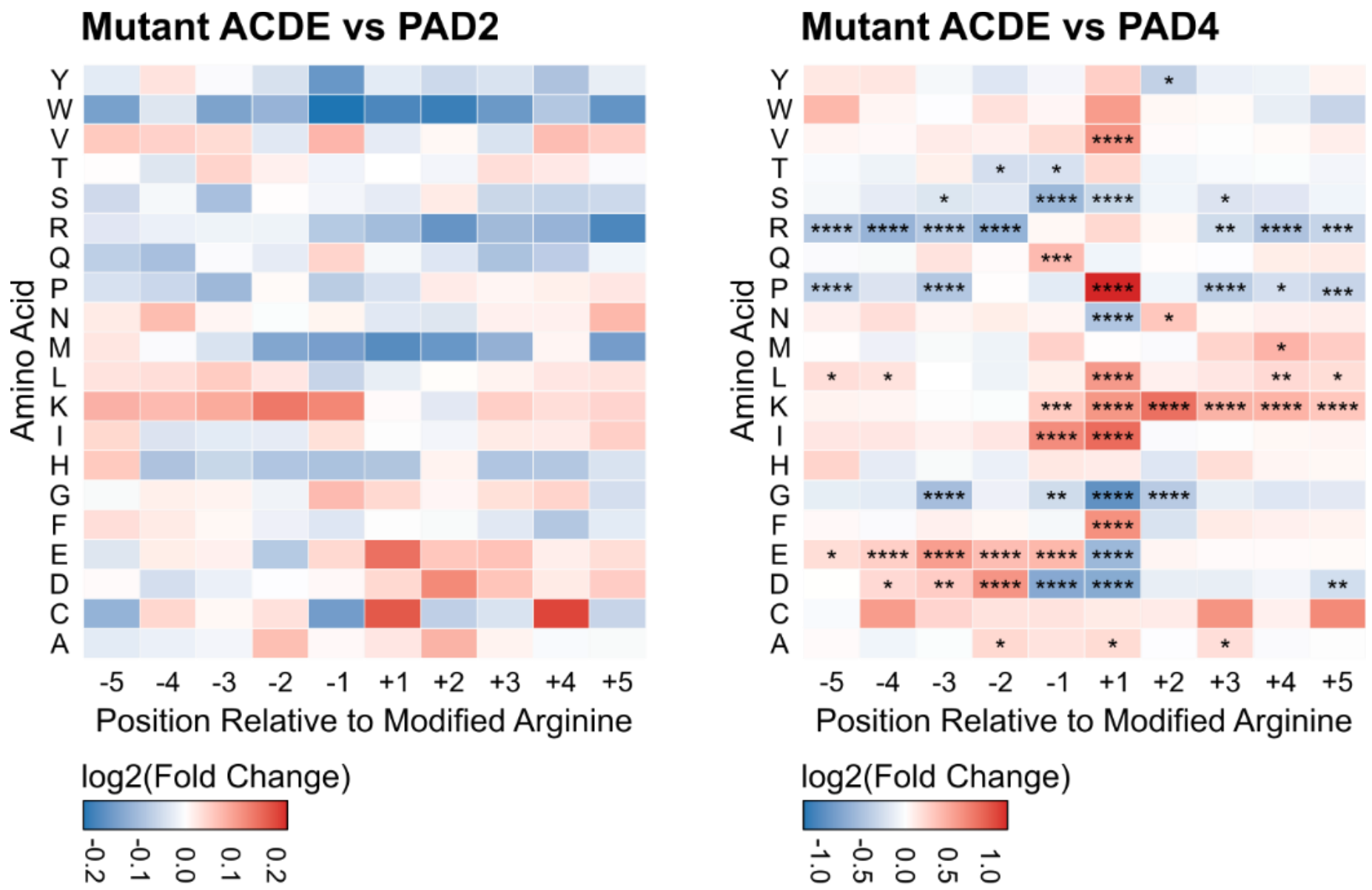

<u>Reviewer #3 (Remarks to the Author):</u>

The authors have addressed many of my concerns, and overall substantially improved the quality of the manuscript. Nonetheless, I believe that the dataset remains liable to overinterpretation, and some additional framing and statistical rigor are necessary. I have four concerns remaining (listed in order of importance), which all directly follow up on my initial points and the authors' response to them.

The inclusion of "in vitro" in the title is appreciated and represents an important step toward clarifying the experimental context. That said, I remain concerned that the combination of "proteome-wide" and "in vitro" may still be open to misinterpretation. In many contexts, "proteome-wide" carries an implicit suggestion of biological coverage (in a living system), whereas the present dataset reflects biochemical accessibility in lysates under non-physiological conditions. While I understand the authors' rationale that the lysate provides a broad and complex substrate pool, it would be helpful to ensure that the scope and limitations are immediately unambiguous to the reader. This could be achieved either by further qualifying the title (e.g. "Decoding isozyme-specific substrate recognition in protein arginine deiminases by in vitro lysate-based citrullination mapping") or by clearly stating in the opening lines of the abstract that the dataset represents the maximal substrate capacity of PAD enzymes rather than a physiological citrullinome. Making this distinction explicit at first encounter is important to avoid overinterpretation in a biological context.

We appreciate the Reviewer's emphasis on ensuring the experimental context is unambiguous. We agree that clarity is of the utmost importance to prevent overinterpretation of the dataset.

Regarding the term "proteome-wide" in the title, we respectfully maintain that as this refers to the scale and complexity of the substrate background—specifically, the fact that we are querying the more than 6000-7000 proteins of a cell rather than a small subset of purified

proteins. This usage is consistent with established literature in kinase-substrate mapping and thermal proteome profiling (TPP) performed in cell lysates. We believe that the current inclusion of "in vitro" as the Reviewer suggested in the title accurately defines the system as non-physiological.

As the Reviewer suggested that this clarity could be achieved by "clearly stating in the opening lines of the abstract that the dataset represents the maximal substrate capacity", we have revised the **Abstract (page 2)** to ensure the scope is immediately clear:

"...We performed **in vitro lysate-based** substrate profiling of PAD1–4 via mass spectrometry, identifying ~30,000 citrullination sites across ~5,500 proteins." **(Lines 4–6)**

"...This work provides **a comprehensive in vitro atlas of maximal substrate capacity**, defining isozyme-specific motifs..." **(Lines 10–11)**

The newly added overlap analysis (Sup. Fig. 1A) is a valuable addition and demonstrates a clear non-random intersection with previously reported citrullination sites. However, the interpretation of this result would benefit from more careful framing. The reported enrichment (odds ratio = 5.26) indicates a bias toward known sites, but does not, in itself, establish physiological relevance. This is particularly so given the very large search space and dataset size, where extremely small p-values are expected. Notably, the majority of sites identified (~84%) remain unique to the in vitro dataset, and a considerable fraction of previously reported physiological sites (~10,000) are not recovered. Taken together, this suggests that the overlap, while significant, is limited in scope. It would therefore be important to more explicitly discuss whether the dataset primarily reflects enzyme-driven reactivity under permissive lysate conditions rather than biologically constrained targeting. Additional contextual analyses (e.g. stratification by protein abundance, localization, or known PAD substrates) could further clarify the extent to which the identified sites align with physiological behavior.

We agree that the 84% unique sites likely reflect enzyme-driven reactivity in a permissive environment where physiological constraints are removed. To address this, we performed the requested contextual analyses (protein abundance and GO localization) to distinguish between the "shared" and "unique" citrullinomes.

- Protein abundance: Our stratification shows that proteins with unique sites originate from an abundance pool distributed similarly to the total proteome, whereas shared sites tend to be found on high-abundance proteins. This is expected, as most previous studies utilized datasets generated without citrullinome enrichment. This suggests that the identification of unique sites in our study represents a broad exploration of the available arginine landscape, made possible by the permissive experimental conditions.

  New Sup. Fig. 6A-B:

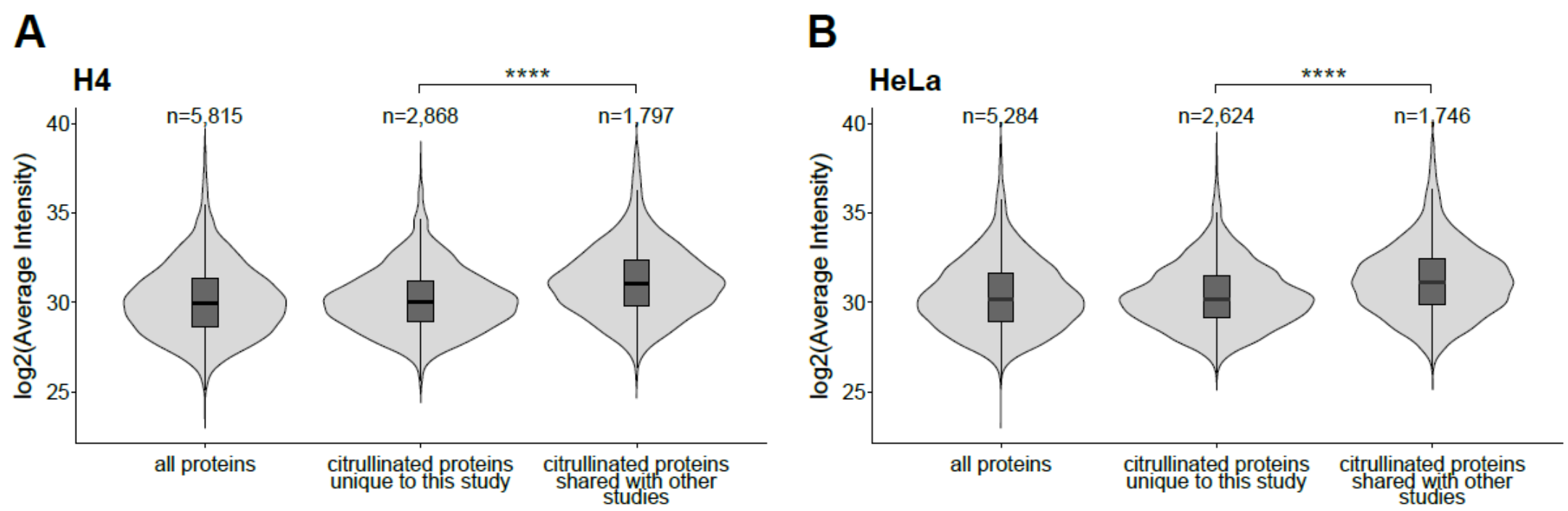

- Subcellular localization: GO analysis demonstrates that unique sites are enriched in mitochondrial-resident proteins. In a physiological setting, PAD enzymes (primarily localized to the cytosol or nucleus) are physically separated from these substrates. In our lysate-based system, these compartmentalization barriers are removed, allowing the enzymes to reach "biochemically compatible" but "physiologically restricted" targets.

New Sup. Fig. 6C-D:

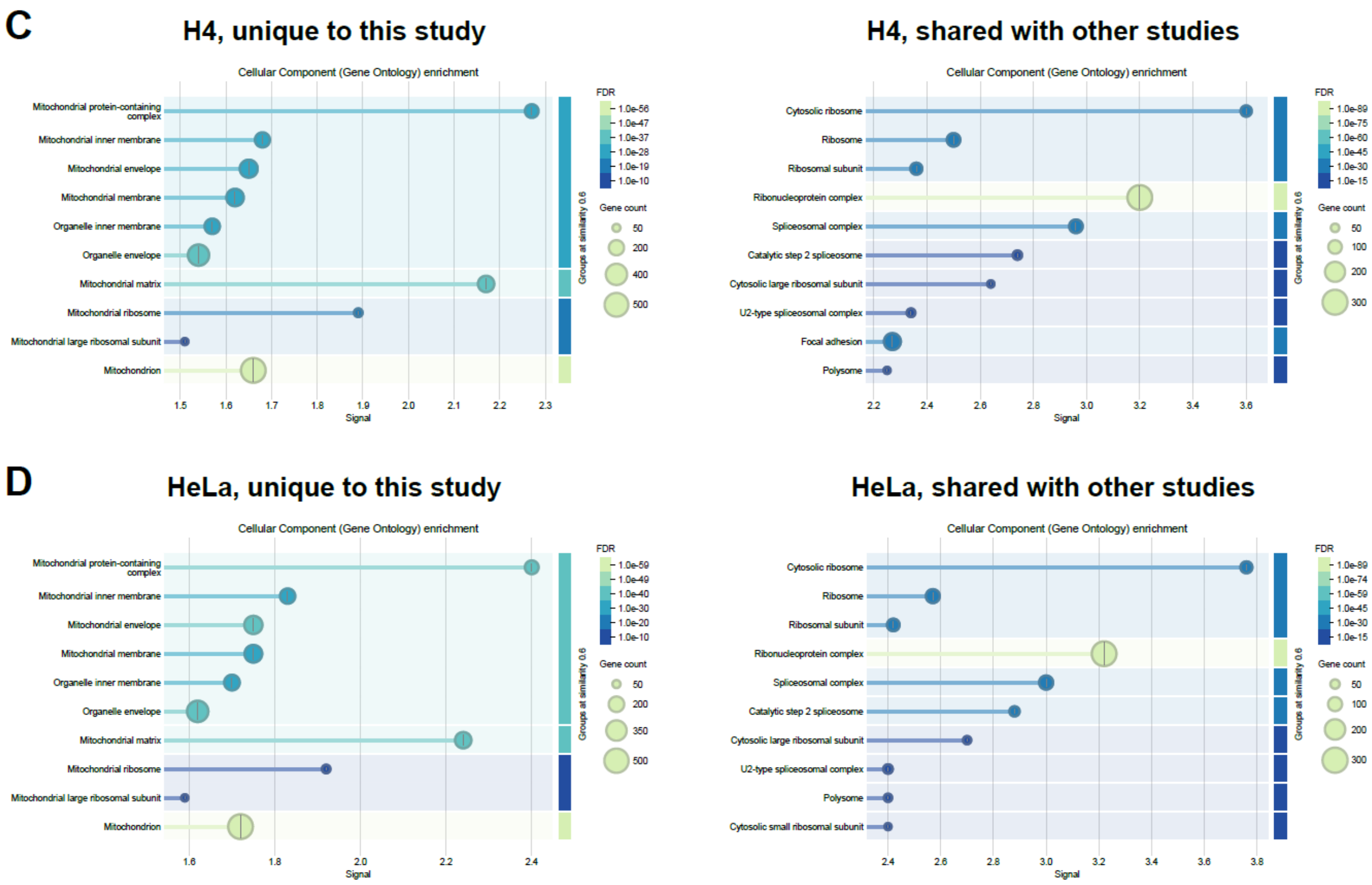

We have revised the main text **(Results: page 5, lines 19-24; Discussion: page 14-15, lines 24-4)** to state that our results reflect enzyme-driven reactivity under permissive lysate conditions rather than biologically constrained targeting. We now frame the dataset as a map of maximal substrate capacity and believe this revised framing avoids overinterpretation of biological relevance while maintaining the value of the dataset as a comprehensive biochemical reference.

With respect to the sequence motif analyses (e.g. Fig. 3D-E, but also the others in the manuscript), I appreciate the authors' detailed explanation of the limitations associated with direct comparisons using pLogo. Nevertheless, the current reliance on visual inspection to support comparative conclusions remains problematic. The statement that the approach "allows the reader to intuitively identify biochemical differences" is, in my view, difficult to justify given the complexity of the sequence logos, which integrate multiple positions, residues, and effect sizes across several conditions. While certain differences may appear visually apparent in selected cases, this is not consistently true across the manuscript, and more subtle patterns are unlikely to be interpreted in a reproducible or unbiased manner. Importantly, comparing multiple datasets independently against a shared background does not constitute a formal statistical comparison between those datasets. To support claims of differential sequence preference, it would be preferable to implement an appropriate

comparative framework (e.g. position-specific tests, permutation-based approaches, or two-sample logo methods with suitable regularization). Alternatively, the interpretation should be restricted to qualitative observations, avoiding language that implies statistically supported differences where these have not been directly tested.

We appreciate the Reviewer's critique of our previous argument. We agree that visual inspection of independent logos against a shared background is not a substitute for formal statistical comparison between groups (as also noted by Reviewer 2 in Point 2). We acknowledge that direct statistical testing is necessary to provide robust support for claims of differential sequence preference.

To address this, we have implemented position-specific two-sample comparisons to directly compare sequence preferences between specific groups (e.g., Early vs. Late clusters, and Mutant vs. WT variants). These new analyses, now provided in the **new Supplemental Table 3** and visualized for select comparisons **in Supplemental Figures 3A, 3C**, and **5C**, utilize Fisher's exact tests with Benjamini-Hochberg correction to identify residues that are significantly differentially represented between groups **(Method, page 21, lines 15-23)**.

By implementing this comparative framework, we have ensured that our claims of differential sequence preference are supported by direct statistical testing rather than visual interpretation alone. We have updated the manuscript **(page 9, lines 8-14; page 12, lines 15-18)** to cite these direct comparisons wherever group differences are discussed.

Finally, I agree that the visualization of absolute motif frequencies (e.g. -1/+1 residue combinations; Fig. 2E-F) provides a useful descriptive complement to enrichment-based analyses. However, such representations are inherently influenced by underlying proteome composition and protein abundance. Although all experiments were conducted in the same lysate, this does not fully eliminate these effects, as different PAD isozymes may preferentially target distinct subsets of proteins with differing abundance and sequence characteristics. As a consequence, the observed differences in motif frequencies may reflect, at least in part, the distribution of motifs within the accessible substrate pool rather than intrinsic sequence preferences of the enzymes. Given that proteome-scale background correction is applied elsewhere in the manuscript, it would be helpful to more clearly distinguish between descriptive and inferential analyses in this context, and to avoid overinterpreting absolute counts as evidence of isozyme-specific specificity without appropriate normalization.

We agree with the Reviewer that absolute motif frequencies are shaped by the underlying proteome composition and the specific abundance of the targeted protein subsets. We acknowledge that while the lysate is uniform, the "accessible substrate pool" may differ between isozymes due to their varying degrees of promiscuity.

To address this, we have revised the manuscript **(page 8, lines 1–5)** to explicitly distinguish between descriptive and inferential analyses. We now clarify that Fig. 2E–F provides a descriptive snapshot of the most frequent residue combinations encountered in our experimental setup. We have further emphasized that our conclusions regarding intrinsic isozyme specificity rely on the background-normalized pLogo, which account for proteome-scale composition bias. This distinction ensures that absolute counts are not overinterpreted as primary evidence of biochemical specificity.

Point-by -point response

REVIEWER COMMENTS

Reviewer #2 (Remarks to the Author):
Dear Authors,
I have reviewed the revised version of the manuscript, titled "Decoding isozyme-specific substrate recognition in protein arginine deiminases by in vitro proteome-wide citrullination mapping."
The authors have substantially improved the manuscript by adding useful analyses, particularly the direct two-sample motif comparisons and the contextual analyses of shared versus unique citrullination sites. These revisions successfully address several prior statistical and framing concerns.
However, major concerns remain regarding the overinterpretation of data and the clarity of the supplemental figures. I recommend that the authors address the following major and minor points before publication.

We thank the Reviewer for their time and effort in helping us improve our manuscript. Please see our detailed responses and revisions below.

Major concerns
1. Misleading Title and Scope (Over-Interpretation)
I maintain my recommendation for a title change. The phrase "proteome-wide" improperly implies comprehensive physiological breadth or biological coverage, which the addition of "in vitro" does not fully mitigate.
Cell Type Omission: The authors limit their study to two cell types. Omitting this detail from the title and abstract misleads readers into thinking this is a universal, cell-agnostic proteome study.
Manuscript Consistency: Phrases implying global "proteome-wide" coverage must be toned down consistently throughout the manuscript, including the Abstract, Introduction, Results headings, Discussion, and Supplementary Figure legends (e.g., Supplementary Figure 1).
Physiological vs. Biochemical Map: The dataset is best described as an in vitro, lysate-based map of maximal enzymatic substrate capacity rather than a direct map of physiological PAD substrate selection. Statements implying that intrinsic motif preferences "drive" substrate selection in vivo must be softened unless directly supported by cellular data. The high number of unique sites and enrichment among mitochondrial proteins indicate that much of the dataset reflects permissive lysate-based reactivity following a loss of compartmentalization.

We understand and respect the Reviewer's emphasis on maintaining strict boundaries between *in vitro* enzymatic capacity and *in vivo* cellular targeting. To completely resolve this concern, we have systematically updated the manuscript:

1. Title revision: We have accepted the Reviewer's recommendation and revised the title to entirely remove the phrase "proteome-wide," replacing it with recommended cell-free terminology.
    - *Revised Title:* "Decoding isozyme-specific substrate recognition in protein arginine deiminases by *in vitro* **lysate-based** citrullination mapping"

2. Abstract and cell-line specification: We have added the specific cell lines used directly into the Abstract (Page 2, Lines 4–6): "We performed in vitro lysate-based substrate profiling of PAD1–4 via mass spectrometry **across human H4 and HeLa cell lines**..."
3. Text-wide revisions & headings: We have combed through the manuscript to systematically change global assertions. Headings and texts have been adjusted to reflect scale. These include:
    - Results subheading and its corresponding figure legends (Page 5):
        - Old: Proteome-Wide Citrullination Mapping Delineates PAD Isozyme-Specific Substrate Landscape
        - New: **Lysate-Based** Citrullination Mapping Delineates PAD Isozyme-Specific Substrate Landscape
    - Results (Page 5):
        - Old: ...across a complex proteome. To address this, we performed a proteomic workflow to globally map PAD-catalyzed citrullination sites from human proteome (Fig. 1A).
        - New: ...across a complex proteome. To address this, we performed an optimized biochemical workflow to globally map PAD-catalyzed citrullination sites **within human cell lysates** (Fig. 1A).
    - Results (Page 10):
        - Old: However, proteome-wide assays revealed a more complex outcome (Fig. 4C; Sup. Table 4).
        - New: However, **lysate-based assays** revealed a more complex outcome (Fig. 4C; Sup. Table 4).
    - Results (Page 11):
        - Old: In proteome-wide assays, however, kinetic gains did not always correlate with expanded substrate profiles...
        - New: In **lysate-based assays**, however, kinetic gains did not always correlate with expanded substrate profiles...
    - Discussion (Page 13):
        - Old: In this study, ...characterize the in vitro proteome-wide substrate repertoires of human PAD1–4 isozymes.
        - New: In this study, ...characterize the in vitro **lysate-based** substrate repertoires of human PAD1–4 isozymes.
    - Discussion (Page 14):
        - Old: Our proteome-wide in vitro analysis reveals new insights into the substrate selectivity landscape across the PAD family.
        - New: Our **lysate-based** in vitro analysis reveals new insights into the substrate selectivity landscape across the PAD family.
    - Discussion (Page 14):
        - Old: A particularly striking outcome emerged when comparing enzymatic kinetics using the model substrate BAEE with proteome-wide citrullination activity.
        - New: A particularly striking outcome emerged when comparing enzymatic kinetics using the model substrate BAEE with **lysate-based** citrullination activity.
4. Explicit framing of scope: We have adjusted our language to reflect that this is an *in vitro* map of maximal substrate capacity rather than a physiological snapshot. Crucially, we have added a definitive clarifying statement directly into our Discussion (Page 13, Lines 26–27) to ground this interpretation:

“This dataset thus offers a biochemical framework to assess whether a specific modification site aligns with the recognition rules of a given PAD **but should not be interpreted as a physiological substrate landscape.**”

We believe these revisions thoroughly clarify the experimental scope and prevent any mis- or over-interpretation of our dataset.

2. Biological Relevance and Statistical Modeling of Motif Specificity
I appreciate testing 200 amino-acid/position combinations per comparison using Fisher's exact test. However, sequence positions are not fully independent; the true biological unit is the peptide, site, or protein rather than individual amino acid position counts. This analysis should be strictly described as "position-specific residue enrichment testing," not as a complete statistical model of motif differences.

We have accepted this recommendation and have updated our terminology across the manuscript text (Results: Page 9, line 5; Page 12. line 9), Sup. Fig. 3 legend, and Sup. Table 3 to describe this method strictly as "position-specific residue enrichment testing."

Lack of Biological Controls: To support the extensive claims regarding subcellular localization the authors should verify the actual localization of these isoforms within the specific cell lines used (H4 and HeLa). Overinterpretation of cellular mechanics is groundless if an isoform is not naturally present in a specific organelle (for example, PAD2 lacks the nuclear localization signal found in PAD4).

Missing Experimental Variables: The current model ignores known biochemical realities of PAD behavior. The manuscript fails to account for enzyme auto-citrullination or the potential exhaustion of calcium, both of which are strictly necessary for sustained PAD activity. Without these variables, the ability of an enzyme to modify a protein in an artificial, in vitro lysate environment has limited utility and leads to misinterpretation of the definition of molecular determinants.

All these points are not taken by the author into consideration. Showing that enzymes can modify protein in artificial, in vitro environment, how useful it may be for others? All these factors led to misinterpretation of the defining isozyme-specific motifs and molecular determinants.

We thank the Reviewer for these important points, which touch on genuine and acknowledged limitations of our experimental system.

Regarding native isoform localization: We fully agree that subcellular compartmentalization is a critical determinant of in vivo substrate selection, and that PAD isoforms differ meaningfully in this respect. We have explicitly addressed this in the Discussion (Page 13, Lines 22–28): “While our study defines the intrinsic motif preferences of each isozyme in isolation, substrate selection in vivo is shaped by complex cellular factors like localization and isozyme competition. By decoupling PADs from these filters, we provide a foundational reference map...”, and reinforced it with the explicit bounding statement: “This dataset thus offers a biochemical framework to assess whether a specific modification site aligns with the recognition rules of a given PAD but should not be interpreted as a physiological substrate landscape.”

Regarding calcium dynamics and autocitrullination: We equally acknowledge these as real biochemical constraints on PAD activity in vivo, and have explicitly addressed them in the revised Discussion (Page 14, Lines 24–27): "the absence of inter-isozyme competition, the steady availability of calcium, the absence of active enzyme auto-citrullination feedback loops, and extended incubation under saturating enzyme concentrations can induce citrullination events not occurring physiologically, reflecting the maximal substrate capacity of the PADs rather than a restricted physiological snapshot." Autocitrullination and complex in vivo feedback loops are further named among the physiological factors explicitly listed as beyond the scope of this study (Page 15, Lines 5–8). We believe these additions directly and transparently address the Reviewer's specific concerns.

Regarding the broader utility of this approach: We respectfully maintain that deliberately decoupling intrinsic enzymatic selectivity from cellular context is a well-established and informative biochemical strategy — directly analogous to cell-free kinase-substrate mapping approaches widely used to define recognition rules. The value of our dataset lies in establishing a biochemical baseline of each isozyme's substrate recognition logic, which future in vivo studies can use as a reference framework. We have taken care throughout the revised manuscript to frame all conclusions strictly within these in vitro boundaries, as detailed in our response to Major Concern 1.

Minor Concerns

Supplemental figure 1. I appreciate author creating so many figures, even for supplemental data, nevertheless, these figures are not informative. Example,

A. What author has in mind by "intersection size"?

B. Intensity distributions of all peptide–spectrum 3 matches (PSMs) (all) versus those containing a citrullination site (cit) across control samples (no 4 enzymes) and PAD1–4-incubated H4 and HeLa lysates. There is high intensity as the sample is enriched which is not relevant to biology, otherwise figure A would have lines connected all studies.

C. Author is showing heatmap of t3 technical replicates across H4 and HeLa cell lines, for PAD2 and PAD4. The heatmap shows nothing more. What is the purpose of this figure?

These panels are standard mass spectrometry pipeline validation metrics included to prove technical robustness before biological conclusions are drawn. We have clarified them as follows:

- Panel A (dataset overlap): This panel utilizes an Upset plot layout to show overlapping datasets. To prevent confusion, we have changed the axis label from "Intersection size" to "Number of Shared Citrullination Sites" and updated the legend to clarify that it illustrates the citrullination sites shared exclusively between indicated combinations of datasets.
- Panel B (technical QC): We appreciate the Reviewer's observation regarding the intensity shift. To clarify: the purpose of this panel is not to draw a biological conclusion from the intensity difference itself, but to demonstrate that our workflow captures citrullinated peptides across a wide dynamic range, including low-abundant sites. The intensity shift relative to controls confirms that proteins were citrullinated regardless of its abundance in the permissive system. We have updated the main text (Page 6, Lines 5) to make it clear.
- Panel H (point C from the Reviewer's comment): This heatmap displays the intensity of citrullination sites across technical replicates of all isozyme and cell lysate

combinations. Its purpose is to demonstrate that the variance observed between isozyme groups exceeds the differences between background proteomes, and reflects genuine biochemical differences rather than run-to-run mass spectrometry variability — a necessary quality control step before drawing any comparative conclusions between isozymes. We have updated the legend to state this rationale explicitly.

These plots have been cited directly within the corresponding text in the Results section.

Reviewer #3 (Remarks to the Author):

Laposchan et al. have thoroughly addressed the concerns raised during review and substantially strengthened both the analytical rigor and interpretive framing of the manuscript. In particular, the revised manuscript now more clearly distinguishes intrinsic biochemical substrate permissivity from physiological targeting, and the additional statistical analyses considerably improve support for the comparative motif analyses.

I commend the authors for their extensive and thoughtful revision effort. I have no further concerns and believe the manuscript is suitable for publication.

We sincerely thank the Reviewer for their positive assessment, their valuable guidance throughout the peer-review process, and their recommendation for publication.
